# Functional Mapping of Neurodevelopmental Disease Pathways to Key Neurodevelopmental Processes Represented in the Developmental Neurotoxicity In Vitro Testing Battery

**DOI:** 10.1002/advs.202519889

**Published:** 2026-04-14

**Authors:** Eliska Kuchovska, Kristina Bartmann, Georgea Raad, Mats Schade, Luiz Ladeira, Arif Dönmez, Jördis Klose, Nicolai Görts, Denis Polozij, Lynn‐Christin Saborowski, Farina Bendt, Bernard Staumont, Liesbet Geris, Katharina Koch, Ellen Fritsche

**Affiliations:** ^1^ IUF – Leibniz Research Institute for Environmental Medicine Düsseldorf Germany; ^2^ DNTOX GmbH Düsseldorf Germany; ^3^ GIGA Molecular and Computational Biology University of Liège Belgium; ^4^ Skeletal Biology and Engineering Research Center KU Leuven Belgium; ^5^ Biomechanics Section KU Leuven Belgium; ^6^ SCAHT – Swiss Centre for Applied Human Toxicology & Department of Pharmaceutical Sciences University of Basel Switzerland

**Keywords:** biological applicability domain, developmental neurotoxicity, neurosphere assay, new approach methodology, signaling pathways

## Abstract

The Developmental Neurotoxicity (DNT) in vitro battery (IVB) enables efficient and human‐relevant evaluation of chemicals for DNT potential. To expand its biological applicability domain toward human disease, this study maps neurodevelopmental disorder (NDD)‐relevant signaling pathways to key neurodevelopmental processes (KNDPs) using primary human fetal neural progenitor cells (NPCs). Using pharmacological intervention, eighteen NDD pathways are assessed for their impact on seven KNDPs, namely NPC proliferation, radial glia migration, neuronal and oligodendrocyte differentiation and migration, and neurite outgrowth. In total, modulation of sixteen pathways is associated with changes in at least one KNDP. Oligodendrocyte differentiation shows the highest sensitivity (13 pathways), followed by radial glia migration (11 pathways) and NPC proliferation (9 pathways), whereas neuronal migration remains unaffected. Perturbation of the RhoA and mitochondrial complex I pathways is associated with the broadest phenotypic responses, influencing five KNDPs each, while STAT3‐ and TrkB‐related modulation falls outside the assay's applicability domain. Pathway‐KNDP associations are integrated into an exemplary interactive physiological map of human oligodendrocyte development, linking mechanistic perturbations to human‐relevant biology. Defining which NDD pathways can be functionally probed refines the DNT IVB's biological applicability domain, increases confidence in its protective power, and supports mechanistic interpretation of new approach methodology‐based DNT assessment.

## Graphical abstract

Human‐relevant methods are essential for modern chemical safety assessment. This study helps define the capabilities and boundaries of an in vitro testing battery for developmental neurotoxicity by exploring its biological applicability domain. By linking neurodevelopmental disease‐related pathways to key neurodevelopmental processes, the work enhances confidence in non‐animal methods for regulatory use.

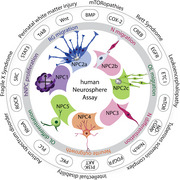

## 1 Introduction

Developmental neurotoxicity (DNT) refers to the harmful effects of substance exposure on the developing brain. Currently, DNT assessment is not mandatory for compound registration other than biocides, yet the in vivo guideline studies—such as OECD TG 426 and 443—may be triggered in the European Union within the plant protection product or the REACH (Registration, Evaluation, Authorisation and Restriction of Chemicals) legislation in case of concerns, e.g., adult neurotoxicity or endocrine disruption [1]. In the United States, the EPA OPPTS 870.630 guideline is primarily applied for pesticides, where DNT testing can be a data requirement. There are certain issues with these procedures. First, these triggers might not cover neurodevelopmental effects sufficiently [2]. Second, these studies are far too resource‐intensive to be adequate for assessing the potential DNT hazard of the human exposome [3], i.e., requiring over a year with a cost of over one million euros/dollars per chemical. Third, while these studies are valuable for identifying apical toxicity outcomes, they do not provide mechanistic information, limiting their usefulness for understanding underlying biological perturbations [4]. Fourth, the use of animals in toxicity testing is burdened with ethical issues; in one guideline DNT study, e.g. OECD TG 443, around 1000 rodent pups are usually sacrificed [5, 6, 7]. Last, DNT in vivo studies are accompanied by a large number of uncertainties [4] including limited predictivity for human risk assessment due to challenges in extrapolating animal data to humans [8]. These limitations arise from interspecies differences in brain development, including anatomy, kinetics, metabolism, lipidome, cell‐type specification, and molecular signaling [4, 9, 10, 11, 12]. For example, species‐specific expression of receptors such as the aryl hydrocarbon receptor (AhR) can alter susceptibility to toxicants [9], while the human brain exhibits a distinct and evolutionarily specialized lipidome compared to rodents and non‐human primates [10]. More broadly, human neurodevelopment is characterized by prolonged developmental trajectories, increased cellular and circuit complexity, and species‐specific gene functions [11, 12]. These issues lead to a large knowledge gap for the DNT potential of most of the at least 30 000 chemicals of our exposome including environmental chemicals (estimated based on REACH chemical registration estimates). Indeed, only approximately 150 chemicals have so far been assessed in such regulatory DNT in vivo guideline studies with publicly available data, as compiled in a dedicated repository in 2024 [13]. Therefore, an urgent need for more reliable, efficient, and better predictive human‐based new approach methodologies (NAMs) for DNT testing has been voiced by multiple stakeholders including regulatory scientists [2, 14, 15, 16].

**FIGURE 1:**
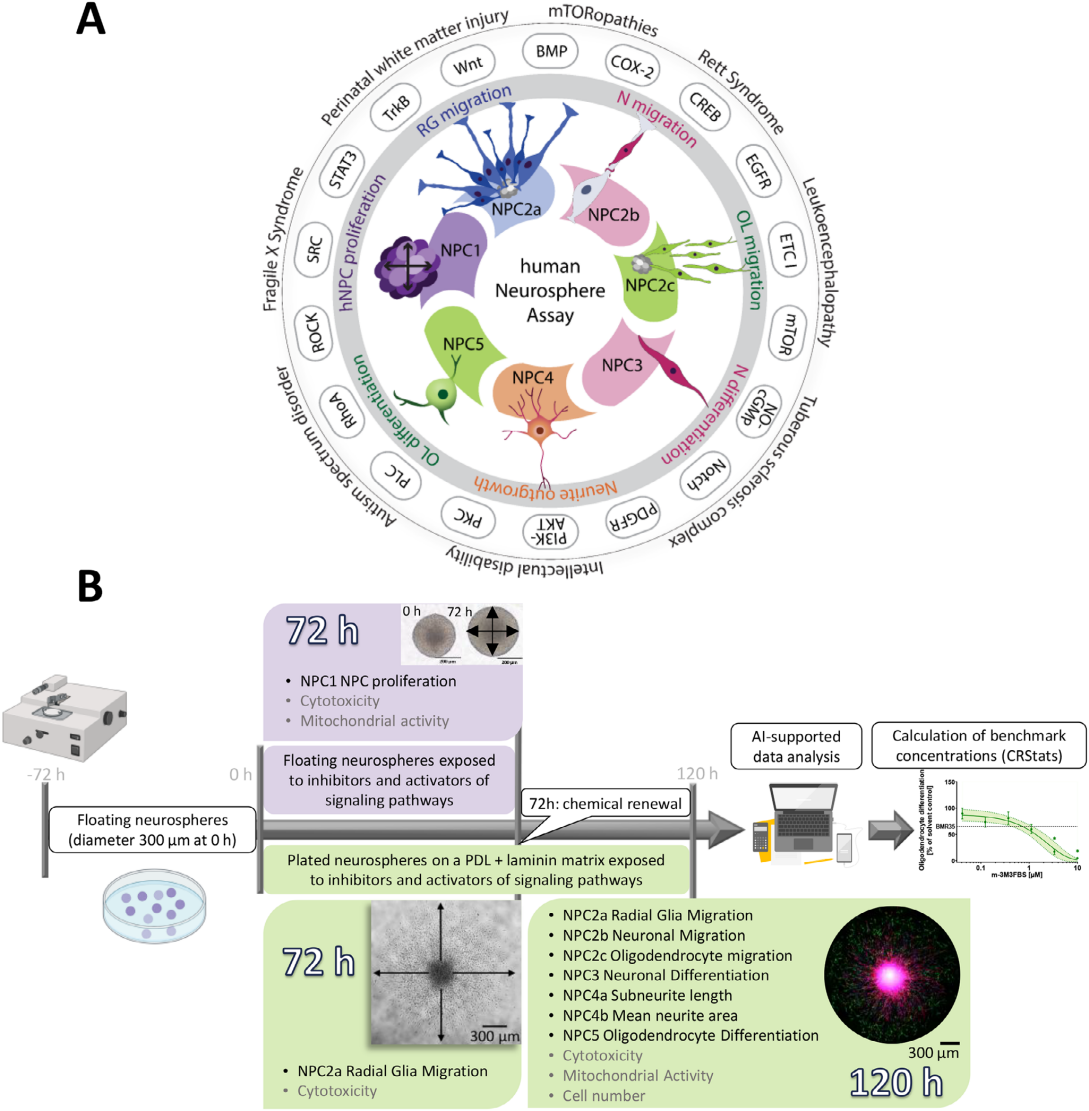
Schematic overview of the human Neurosphere Assay test methods. **A** The inner ring contains the key neurodevelopmental processes modeled in the individual NPC assays. Neurite outgrowth covers two different endpoints: neurite length and area. These key events are complemented by cell number, viability, and cytotoxicity assays. The middle ring highlight the signaling pathways investigated in this manuscript. The outer ring indicates examples of neurodevelopmental disorders with impaired key neurodevelopmental events. **B** Exposure setup of the Neurospheres Assay. Proliferating hNPC neurospheres were mechanically passaged to reach an average sphere size of 300 µm in diameter on the plating day. hNPCs were subsequently exposed for 3 days to inhibitors and activators of the selected signaling pathways as free floating neurospheres in a medium supplemented with growth factors to asses proliferation (NPC1) as shown in purple boxes. In addition, hNPC neurospheres were plated on PDL and laminin matrix and exposed to the same compounds in a differentiation medium without growth factors (green boxes). After 5 days, cells radially migrated cells out of the sphere core and immunocytochemical stainings were carried out (neurons are highlighted in red (β(III)tubulin), oligodendrocytes in green (O4), and nuclei in blue (Hoechst33258) to assess the endpoints in NPC2‐5 assays. The data were analyzed in an R‐based statistical pipeline and benchmark concentrations were calculated. **Abbreviations**: hNPC = human neuroprogenitor cells, RG = radial glia, N = neuronal, OL = oligodendrocyte, PDL = Poly‐D‐lysine, AI = artificial intelligence, BMP = bone morphogenetic protein, COX‐2 = Cyclooxygenase‐2, CREB = cAMP response element‐binding protein, EGFR = epidermal growth factor receptor, ETC I = mitochondrial electron transport chain complex 1, mTOR = mammalian target of rapamycin, NO‐cGMP = Nitric oxide–cyclic guanosine 3′,5′‐monophosphate, PDGF = Platelet‐derived growth factor, PI3K = phosphatidylinositide 3‐kinase, PKC = protein kinase C, PLC = phospholipase C, RhoA = Ras homolog family member A, ROCK = Rho‐associated protein kinase, STAT3 = Signal transducer and activator of transcription 3, TrkB = tropomyosin receptor kinase B.

To address this need, the DNT in vitro battery (IVB) for hazard identification, characterization, and risk assessment was assembled [1, 3, 17, 18, 19] building on long‐standing efforts of the academic and regulatory DNT community [20, 21, 22, 23]. These efforts culminated in the report for interpreting data from the DNT IVB, for use in the Integrated Approaches for Testing and Assessment (IATA) [3] and the OECD endorsement via the guidance document number 377 [1]. The DNT IVB comprises 17 assays to date (including 3 rat‐based assays) covering key neurodevelopmental processes (KNDPs) such as human neural progenitor cell (hNPC) proliferation and apoptosis, migration of radial glia (RG), neurons, oligodendrocytes, and neural crest cells, as well as neuronal differentiation, neurite outgrowth, synaptogenesis, oligodendrocyte differentiation, and neural network formation. The assays are complemented with viability and/or cytotoxicity assays and employ different test systems, including human neural stem cells (NSCs), NPCs, and rat primary cells [3]. Given the complexity of brain development, the use of multiple assays enhances physiological coverage across different developmental windows. For example, the Neurosphere Assay (Figure 1), which is an integral part of the DNT IVB, models several KNDPs relevant to fetal brain development using primary hNPCs [24, 25]. The assays within the battery have undergone substantial fit‐for‐purpose scientific validation [19, 24] while formal regulatory validation, such as inter‐laboratory testing, is currently ongoing.

NAMs enable human‐relevant, time‐ and cost‐efficient hazard assessment, e.g. the assessment of one chemical in the Neurosphere Assay is estimated to take up to 2 months and cost around 20 thousand euros and the savings associated with using all the DNT IVB assays were internally calculated to exceed 90% and take 5 months when compared to traditional in vivo DNT testing (Extended One‐Generation Reproductive Toxicity Study (TG 443) with DNT‐specific cohorts that has been estimated to cost up to 1.5 million euros per chemical and take 1 year) [5]. However, NAMs also introduce uncertainties into chemical testing—such as limited representation of anatomical complexity, restricted biological coverage, and lack of primary metabolism. Validation of non‐animal NAMs therefore requires, among other essential aspects, comprehensive characterization of their biological applicability domains [25, 26, 27]. While the OECD guideline documents 211 [28] and 34 [29] outline stringent validation requirements, recent proposals advocate for flexible, fit‐for‐purpose scientific validation of NAMs with a strong focus on their biological relevance and coverage [15, 30, 31, 32, 33]. At the same time, OECD acknowledges the substantial operational and financial burden of such validation efforts [34] and continues to emphasize the importance of robust approaches such as ring trials to ensure reproducibility and confidence in regulatory decision‐making [35]. One major pillar of the scientific validation approach concerns the biological relevance of the test method, i.e., for DNT, the measure of the KNDP [30]. The physiological coverage of the test method will define its biological applicability domain [36]. The biological relevance of the DNT in vitro NAMs has been addressed in in‐depth studies previously [1, 24, 37, 38]. For instance, Notch pathway inhibition in hNPCs during differentiation increases neuronal and reduces oligodendrocyte differentiation in vitro [24]. This pathway is key during brain development, as evidenced by in vivo studies [39, 40, 41, 42].

Physiological maps are valuable tools for summarizing biology as a basis for understanding the biological applicability of NAMs. These graphical, interactive, standardized, and machine‐readable representations detail organ function and homeostasis at molecular and cellular levels [43, 44], integrating expert‐curated knowledge of signaling, metabolic, and gene regulatory networks [45, 46, 47]. Experimental studies with NAMs can map these pathways to physiology [24]. Physiological maps integrate state‐of‐the‐art knowledge but also support the development of Adverse Outcome Pathways (AOP) by enhancing biological plausibility [48, 49] and serve as frameworks for predictive in silico toxicology models [50, 51]. This integration reduces uncertainty in human risk assessment. To ensure that NAMs protect human health rather than predict animal data [36], physiological maps must be human‐specific due to numerous species‐specificities in physiology and disease [4, 52, 53, 54, 55, 56, 57]. While we learned tremendously from animal studies that provided basic insights into brain development, the devil lies in the details and many molecules and pathways function differently across species. Examples include the N‐glycome in brain glycosylation linked to human neurodevelopmental disorders (NDDs) [58], the human‐specific NOTCH2NL receptor affecting neuronal output [59, 60], unique thyroid hormone signaling patterns [61], reactive oxygen species responses [62], and diseases like the Cockayne Syndrome B [63]. Species‐specificities contribute to the high attrition rates in drug development for brain‐related diseases indicating the functional relevance of molecular and cellular species‐specificities [64, 65]. Therefore, it is most important to build physiological maps for human brain development derived from human data including omics analyses of human fetal tissue [66, 67, 68], human 3D neurospheres [37, 69], BrainSpheres [70, 71], or organoids [72, 73].

In addition to the biological applicability domain supported by physiological maps, we believe that elucidating NDD pathways regulating KNDPs assessed by DNT IVB methods can greatly enhance their value in understanding how chemicals may contribute to developmental disorders of the human brain. Therefore, we compiled a non‐exhaustive summary of literature examples containing the known pathophysiological contributions in which dysregulation of selected signaling pathways has been associated with altered KNDPs in NDDs, focusing exclusively on patient and human in vitro studies excluding animal data (Table 1). In this context, we refer to the pathophysiological applicability domain as the range of neurodevelopmental disease‐relevant signaling pathways that can be functionally assessed within the Neurosphere Assay by their measurable impact on key neurodevelopmental processes (KNDPs). This complements the biological applicability domain, which defines physiological coverage of the test method. The main aim was to examine how modulation of 18 signaling pathways affects the 7 endpoints of the human Neurosphere Assay (Figure 1). A secondary aim was to create an exemplary physiological map as a proof‐of‐concept, that is centered on oligodendrocyte differentiation, a KNDP that is highly pathognomonic when disturbed [74]. Ultimately, this study aims to bolster the regulatory acceptance of DNT NAMs by increasing confidence in their human‐protective potential.

**TABLE 1:** Selected signaling pathways involved in the pathology of human neurodevelopmental disorders.

Signaling pathway	Related human neurodevelopmental disorders
**BMP**	*CASK*‐related NDDs such as ASD [236]; FXS [237]; microphthalmia with brain anomalies [238]
**COX‐2**	ASD [239]; neuropsychiatric disorders [239]; perinatal white matter injury [109]
**CREB**	Rubinstein–Taybi syndrome with intellectual disability [132]; Rett Syndrome [240]; learning function [241]; intellectual disability, ASD, FXS, schizophrenia [242]
**EGFR**	ASD [243, 244]; microcephaly [245, 246]
**ETC complex I**	Leigh Syndrome [143]; cavitating leukoencephalopathy [247]
**mTOR**	ASD [248, 249]; tuberous sclerosis complex [250, 251]; mTORopathies [252, 253]; FXS [254]; intellectual disability with megalencephaly [147]
**NO‐cGMP**	ASD [255]; Down Syndrome, FXS, Rett Syndrome, intellectual disability [242]
**Notch**	ASD, intellectual disability [256, 257]; focal cortical dysplasia [258]
**PDGF**	ASD [259, 260, 261]
**PI3K‐AKT**	ASD [262, 263, 264, 265]; brain overgrowth‐associated disorders [181]; FXS [177]; malformations of cortical development (e.g., megalencephaly) [182]; megalencephaly [174]; schizophrenia [266]
**PKC**	ASD [267]; spinocerebellar ataxia [268]; bipolar disorder [269]
**PLC**	ASD, bipolar disorder [194]; schizophrenia [270]; epilepsy [271]
**RhoA**	Intellectual disability [272]; developmental language disorder [273]
**ROCK**	Intellectual disability [272, 274]; motor disorders [275]
**SRC**	Noonan syndrome [276]
**STAT3**	Hypoxic–ischemic encephalopathy [277]
**TrkB**	Rett syndrome [278]; schizophrenia [279, 280]; bipolar disorder [280, 281]
**Wnt**	ASD [217, 282, 283]; FXS [284]; Miller‐Dieker syndrome, lissencephaly [213]

This table provides non‐exhaustive examples from peer‐reviewed literature where the listed pathways have been implicated in neurodevelopmental disorders through disruption of key neurodevelopmental processes (KNDPs). The intention is to illustrate the translational plausibility of our pathway selection and to guide the reader to relevant human‐relevant studies, such as those using patient‐derived iPSC models. The table does not aim to provide quantitative or causal assessments of disease mechanisms, nor does it capture the directionality or strength of pathway effects, which are context‐dependent and often vary across developmental stages, cell types, and genetic backgrounds.

**Abbreviations**: ASD = autism spectrum disorder, FXS = fragile X syndrome, BMP = bone morphogenetic protein, COX‐2 = Cyclooxygenase‐2, CREB = cAMP response element‐binding protein, EGFR = epidermal growth factor receptor, ETC I = mitochondrial electron transport chain complex 1, mTOR = mammalian target of rapamycin, NO‐cGMP = Nitric oxide–cyclic guanosine 3′,5′‐monophosphate, PDGF = Platelet‐derived growth factor, PI3K = phosphatidylinositide 3‐kinase, PKC = protein kinase C, PLC = phospholipase C, RhoA = Ras homolog family member A, ROCK = Rho‐associated protein kinase, STAT3 = Signal transducer and activator of transcription 3, TrkB = tropomyosin receptor kinase B.

## 2 Materials and Methods

### 2.1 Basic hNPC Neurosphere Cell Culture

Primary human fetal NPCs (hNPCs), isolated from cortices of gestational week 16–19 fetuses, were purchased from Lonza Verviers SPRL, Belgium (#PT‐2599). They were cultivated as free‐floating 3D neurospheres in proliferation medium (Table 2) at 37 °C and 5% CO_2_ in cell culture dishes coated with poly‐2‐hydroxyethyl methacrylate (poly‐Hema; #P3932, Merck, United States (US)) and fed by replacement of half of the media 3 times per week. Neurospheres were mechanically passaged to 0.2 mm edge length using a McIlwain tissue chopper (#TC752, Campden Instruments, United Kingdom).

**TABLE 2:** Composition of the proliferation and differentiation culture media used for culturing human neural progenitor cells (hNPCs) in the Neurosphere Assay.

	Concentration	Catalogue number and supplier
**Proliferation medium**		
DMEM and Ham's F12 media	2:1 ratio (v:v)	#31966‐021, Thermo Fisher, US; #31765‐027, Thermo Fisher, US
Penicillin	100 U/mL	#P06‐07100, Pan‐Biotech, Germany
Streptomycin	100 µg/mL	#P06‐07100, Pan‐Biotech, Germany
B27 supplement	2%	#17504044, Thermo Fisher, US
Epidermal growth factor (EGF)	20 ng/mL	#PHG0313, Thermo Fisher, US
Fibroblast growth factor (FGF)	20 ng/mL	#233‐FB, R&D Systems, US
**Differentiation medium**		
DMEM and Ham's F12 media	2:1 ratio (v:v)	#31966‐021, Thermo Fisher, US; #31765‐027, Thermo Fisher, US
Penicillin	100 U/mL	#P06‐07100, Pan‐Biotech, Germany
Streptomycin	100 µg/mL	#P06‐07100, Pan‐Biotech, Germany
N2 supplement	1%	#17502‐048, Thermo Fisher, US

The use of trade names is for identification purposes only and does not constitute endorsement by the authors or their institutions.

**Abbreviations**: DMEM = Dulbecco's Modified Eagle Medium, F12 = Ham's F12 Nutrient Mixture, N2 = N2 Supplement, a chemically defined supplement for serum‐free culture of neural cells, US = United States, U/mL = units per mililiter, µg/mL = micrograms per milliliter, v:v = Volume‐to‐volume ratio.

### 2.2 Neurosphere Assay

The Neurosphere Assay includes 7 different endpoints to investigate NPC proliferation (NPC1), RG migration (NPC2a), neuronal migration (NPC2b), oligodendrocyte migration (NPC2c), neuronal differentiation (NPC3), neurite outgrowth (NPC4), and oligodendrocyte differentiation (NPC5), NPC2‐5 accompanied by nuclei count (Figure 1). The assays are described in detail in the ToxTemps and DB‐ALM SOPs annexed to the OECD DNT IVB document [1]. While the NPC1 assay was conducted over 3 days, the NPC2‐5 assessments were performed as a 5‐day multiplexed evaluation. Proliferating hNPCs were continuously exposed to the mechanistic tool compounds targeting the selected pathways for the full 3‐day period. In contrast, differentiating hNPCs were treated with the relevant mechanistic tool compounds over a 5‐day culture period, which included a re‐feeding step on day 3, during which the compound was refreshed. In addition to the specific endpoints, cell viability (CellTiter‐Blue Assay, #G8081, Promega, Madison, US) and cytotoxicity (CytoTox‐ONE Homogeneous Membrane Integrity Assay; #G7891, Promega, Madison, US; lysis control: 45 min, 0.2% Triton X‐100) were additionally assessed in both testing schemes (fluorescence was measured using Tecan infinite M200 Pro reader; ex: 540 nm; em: 590 nm).

Briefly, to assess NPC proliferation (NPC1), neurospheres (300 µm diameter) were exposed to non‐cytotoxic concentrations of investigated mechanistic tool compounds (Table 3) in proliferation medium containing growth factors (Table 2) in poly‐Hema‐coated 96‐well U‐bottom plates for 3 days. Proliferation medium without growth factors was used as an endpoint‐specific positive control. The proliferation was assessed after 72 h of compound exposure by the incorporation of bromodeoxyuridine (BrdU, NPC1, #11669915001, Roche, Switzerland) into the DNA by exposing with BrdU for the last 16 h of the compound treatment [24].

**TABLE 3:** List of mechanistic tool compounds used to activate and inhibit the investigated pathways.

Reagent	Pathway	Activator/Inhibitor	Source	Catalog number	Solvent	Purity	Tested range of concentrations
**PGE2**	COX‐2	Act.	SA	P5640	H_2_O	99%	0.03–20 µM
**Celecoxib**	COX‐2	Inh.	SA	PZ0008	DMSO	≥98%	0.04–30 µM
**db‐cAMP**	CREB	Act.	SA	D0260	H_2_O	99%	13.72–10 000 µM
**KG‐501**	CREB	Inh.	SE	S8409	DMSO	99.8%	0.004–3 µM
**Rotenone**	ETC I	Inh.	SA	R8875	DMSO	96.4%	0.01–10 µM
**MHY1485**	mTOR	Act.	SE	S7811	DMSO	99.9%	0.03–20 µM
**Everolimus**	mTOR	Inh.	SA	SML2282	DMSO	98.6%	0.04–30 µM
**Reelin**	Notch	Act.	R&D	8546‐MR	PBS	>90%	0.034–24.5 nM
**DAPT**	Notch	Inh.	SA	D5942	DMSO	100%	0.01–10 µM
**hPDGF**	PDGFR	Act.	B‐T	221‐AA	HCl/H_2_O	>97%	0.0047–3.4 nM
**CP‐673451**	PDGFR	Inh.	SE	S1536	DMSO	99.7%	0.01–10 µM
**SC79**	PI3K‐Akt	Act.	ME	123871	DMSO	≥98%	0.01–10 µM
**LY294002**	PI3K‐Akt	Inh.	AB	A10547	DMSO	>98%	0.01–10 µM
**m‐3M3FBS**	PLC	Act.	SA	T5699	DMSO	99.9%	0.04–30 µM
**U73122**	PLC	Inh.	MCE	HY‐13419	DMSO	98%	0.007–5 µM
**Colivelin**	STAT3	Act.	TO	3945	EtOH/H_2_O	96%	0.1–94.5 nM
**Limonin**	STAT3	Inh.	SA	L9647	DMSO	99.9%	0.03–20 µM
**BDNF**	TrkB	Act.	TF	10908010	BSA/PBS	96%	0.02551–18.6 µM
**ANA‐12**	TrkB	Inh.	SA	SML0209	DMSO	99%	0.04–30 µM
**CHIR**	WNT	Act.	B‐T	4423	DMSO	≥98%	0.01–10 µM
**IWP2**	WNT	Inh.	B‐T	3533	DMSO	≥98%	0.007–5 µM

**Abbreviations**: Act. = activator, AB = AdooQ Bioscience, BDNF = brain‐derived neurotrophic factor, B‐T = Bio‐Techne, BMP = bone morphogenetic protein, db‐cAMP = dibutyryl cyclic adenosine monophosphate, COX‐2 = Cyclooxygenase‐2, CREB = cAMP response element‐binding protein, EGFR = epidermal growth factor receptor, ETC I = mitochondrial electron transport chain complex 1, Inh. = inhibitor, ME = Merck, MCE = MedChemExpress, mTOR = mammalian target of rapamycin, NO‐cGMP = Nitric oxide–cyclic guanosine 3′,5′‐monophosphate, PDGF = Platelet‐derived growth factor, PGE2 = prostaglandin E2, PI3K = phosphatidylinositide 3‐kinase, PKC = protein kinase C, PLC = phospholipase C, RhoA = Ras homolog family member A, ROCK = Rho‐associated protein kinase, R&D = R&D Systems, SA = Sigma Aldrich, SE = Selleckchem, STAT3 = Signal transducer and activator of transcription 3, TF = Thermo Fisher, TrkB = tropomyosin receptor kinase B, TO = Tocris, UK = United Kingdom, US = United States.

For studying migration and differentiation of hNPCs in the NPC2‐5 assays, neurospheres (300 µm diameter) were plated on 0.1 mg/mL poly‐D‐lysine (#P0899‐50MG, Merck, US) and 12.5 µg/mL laminin (#L2020‐1MG, Merck, US) coated 96‐well plates and exposed to non‐cytotoxic concentrations of mechanistic tool compounds (Table 3) in differentiation medium (Table 2) as described previously [24]. Briefly, the migration distance of RG (NPC2a) from the sphere core was assessed at two time points. After 72 h bright‐field images were taken with an automated microscope (50‐fold magnification, Cellomics ArrayScan) and analyzed manually with the Fiji Image J software [75]. After 120 h of exposure, migration analysis was multiplexed with the assessment of nuclei, neurons, and oligodendrocytes using automatic image analyses of immunocytochemically stained fluorescent images with the software Omnisphero [76]. The cells were first fixed using 4% paraformaldehyde for 30 min at 37 °C, followed by blocking with 10% goat serum (GS; in PBS, 30 min at 37 °C; #G9023‐10 mL, Merck, US). After fixation and blocking, the cells were incubated overnight at 4 °C with primary antibodies (Table 4) in a solution containing 0.01% Triton‐X and 2% GS in PBS. Following PBS washes, the cells were incubated with secondary antibodies (Table 4; 2% GS in PBS) for 60 min at 37 °C. Immunocytochemical images of neurons, oligodendrocytes, and nuclei were then captured using the High Content Analysis platform Cellomics ArrayScan (200‐fold magnification, resolution 552 × 552 pixel) and the provided HCS Studio Cellomics software (version 6.6.0; Thermo Fisher Scientific).

**TABLE 4:** Antibodies and dyes used for immunocytochemical staining in NPC2‐5 assays.

Target	Primary antibody			Secondary antibody		
	Antibody	Dilution	Source	Antibody/Dye	Dilution	Source
**Neurons**	rabbit anti‐β(III)tubulin [EP1569Y]‐Alexa Fluor 647	1:400	#ab190575, Abcam, UK	/	/	/
**Oligodendrocytes**	mouse anti‐O4 IgM	1:400	#MAB1326, R&D systems, US	goat anti‐mouse IgM‐Alexa Fluor 488	1:400	#A‐21042, Thermo Fisher, US
**Nuclei**	/	/	/	Hoechst33258	1:100	#94403‐1ML, Merck, US

**Abbreviations**: UK = United Kingdom, US = United States.

Neuronal (NPC3) and oligodendrocyte (NPC5) differentiation is determined as the percentage of all β(III)tubulin and O4 positive cells, respectively, in percent of the amount of Hoechst positive nuclei in the migration area. Neurite outgrowth (NPC4) was assessed by analyzing the neurite area (NPC4a) and length (NPC4b). Neurite length is the total length in µm of all neurons and the area is the mean area in pixel (without nuclei) of all neurons that are identified by the skeletonization algorithm in Omnisphero (https://omnisphero.com). The stained neurons and oligodendrocytes are identified using two convolutional neural networks based on the Keras architecture implemented in Python 3, which were trained by historical manually analyzed data [77]. The number of nuclei was determined using the SpotDetector (V4.1) bio‐application of the HCS Studio Cellomics software (version 6.6.0, Thermo Fisher Scientific).

Additionally, assay performance of the NPC2–NPC5 endpoints was verified using a set of endpoint‐specific positive controls applied on dedicated validation plates: 10 µM PP2 for radial glia migration (NPC2a), 20 ng/mL EGF for inhibition of neuronal differentiation (NPC3), and 100 ng/mL BMP7 for inhibition of oligodendrocyte differentiation (NPC5).

### 2.3 Mechanistic Tool Compounds Inhibiting and Activating the Investigated Signaling Pathways

All compounds used to inhibit or activate the investigated signaling pathways in the present study are summarized in Table 3 including their source, solvent, substance purity, and stock concentrations. All compounds used to inhibit or activate additional signaling pathways in our previous studies that are discussed in this publication are available in Table S1. Pathways selected for modulation were based on their established involvement in KNDPs and their relevance to DNT, as outlined in the report of the OECD/EFSA workshop on DNT [78]. Pathways primarily related to endocrine disruption, including thyroid hormone signaling, were excluded from this study because they were comprehensively addressed in a parallel study [79]. Compounds were selected for their documented ability to activate or inhibit the respective pathways, as described in peer‐reviewed literature and summarized in Table S3. The selected concentrations were empirically determined to avoid cytotoxicity while ensuring robust modulation of the targeted pathway. Importantly, the aim was not to simulate environmentally or physiologically relevant exposure scenarios, but rather to functionally characterize the responsiveness of the assay system in a pathway‐specific context.

### 2.4 Microarray Data of Human NPCs and Brain Fetal Samples

The expression of relevant proteins in the selected signaling pathways in proliferating and differentiating (60 h) human NPCs was summarized based on our previously published microarray dataset [80]. The analysis is described in the mentioned publication. These results were furthermore complemented with gene expression observed in fetal cortical tissue (postconceptional weeks (pcw) 16 and 21) that are publicly available in the BrainSpan database (https://brainspan.org/) [81]. Expression was averaged for available cerebral cortex regions and different donors (pcw 16—1 donor; pcw 21—2 donors). The BrainSpan raw data are available in Excel Tables S1–S3.

### 2.5 Data Analysis and Statistics

Results are presented as mean ± standard error of the mean (SEM) of 3–4 independent biological replicates represented by hNPCs issued from 3 different individuals (hNPC donors of 16–19 gestational weeks), except for ANA‐12, celecoxib, everolimus, CHIR, KG‐501, limonin, and LY294002 in endpoints NPC2–NPC5, where only two donors of different passages have been used. Passages 1 to 5 were used for the NPC1 experiments, while NPC2–5 experiments were limited to passages 1 to 4 due to a lower oligodendrocyte differentiation rate in higher passages. The majority of experiments were conducted using male donor cells. Both male and female donor cells were used for NPC1 experiments with BDNF, CHIR, KG‐501, limonin, and rotenone, and for NPC2–5 experiments with BDNF, colivelin, CP‐673451, DAPT, db‐cAMP, LY294002, PGE2, SC79, U73122, and rotenone. Assessing the inter‐individual variability of the donors was not practically feasible within the scope of the current experimental design. Similar constraints apply to iPSC‐based models, where variability arises not only from donor differences (e.g., sex, genotype) but also from different neural inductions.

All NPC1 experiments were performed with *N* = 3 independent experiments while for NPC2–5, most compounds were tested with *N* = 3 independent experiments, except for CP‐673451, DAPT, MHY1485, and PGE2, where *N* = 4 experiments were conducted. Each biological replicate corresponds to a median of five technical replicates being one neurosphere each. In exceptional cases, the number of technical replicates was not lower than *n* = 3. Concentration–response data were modeled using the Benchmark Concentration (BMC) approach, as recommended by the EFSA Scientific Committee and the OECD guidance for DNT in vitro testing [1, 82]. This approach defines the BMC as the concentration associated with a predefined Benchmark Response (BMR), allowing biologically meaningful effect levels to be compared across assays. BMR thresholds were selected based on established practices in DNT NAMs to reflect varying assay sensitivities and to ensure robust curve fitting across endpoint types. They are based on the inter‐experiment variability of the lowest compound concentration of the entire dataset of this study, i.e., 63 experiments for each endpoint. Thus, the following BMRs have been selected for the individual assays: BMR15: NPC1; BMR10: NPC2a; BMR30: NPC2b; BMR20: NPC2c, NPC4ab; BMR35: NPC3, NPC5. After determination of the BMC, the upper (BMCU) and lower limit (BMCL) of its 95% confidence interval are calculated using the delta method and the t‐distribution via the “predict” function within the established R package drc [83]. Table containing all BMCUs and BMCLs is available in Table S2. The effect was considered “a hit” when either both BMCL and BMCU intersected the BMR threshold or when the concentration–response curve and one of the confidence intervals intersected the BMR threshold and the change in response at a tested concentration was statistically significant using the step‐down multiple test procedure of Dunnett and Tamhane (*p* ≤ 0.05) [19]. The BMR calculations and biostatistical pipeline have been discussed in our previous publications [19, 84, 85]. and is described as well in the ToxTemps for the Neurosphere Assay published as annexes to the OECD guidance document [1]. To ensure the specificity of the effects on the studied endpoints, only mechanistic tool compounds concentrations that induced less than 10% cytotoxicity were considered (no thresholds have been applied for viability and cell number endpoints). Consequently, benchmark concentration (BMC) BMC10 was determined for the non‐specific cytotoxicity endpoint, and all exposure concentrations above this BMC10 (cytotoxicity) were excluded from the subsequent analysis of the NPC1‐5 endpoints. The specific endpoint and viability data were normalized to their respective solvent controls, while the cytotoxicity data were normalized using dynamic range normalization, with the solvent control and lysis control as references. Additionally, rotenone data, that were tested in the framework of another project in a blinded manner, were further renormalized so that the upper asymptote of the selected curve fit was at 100% [86, 87]. In the renormalization procedure, first, the initial control normalization was carried out, a regression analysis was performed, and the data were fitted. The best‐fitting model was then used to estimate the predicted response at the lowest tested concentration (renormalization coefficient). To account for potential systematic deviations at low concentrations, the control‐normalized data were then adjusted once more by dividing all data points by the renormalization coefficient. A second regression analysis was subsequently performed on the renormalized data, yielding the final concentration–response curve.

**FIGURE 2:**
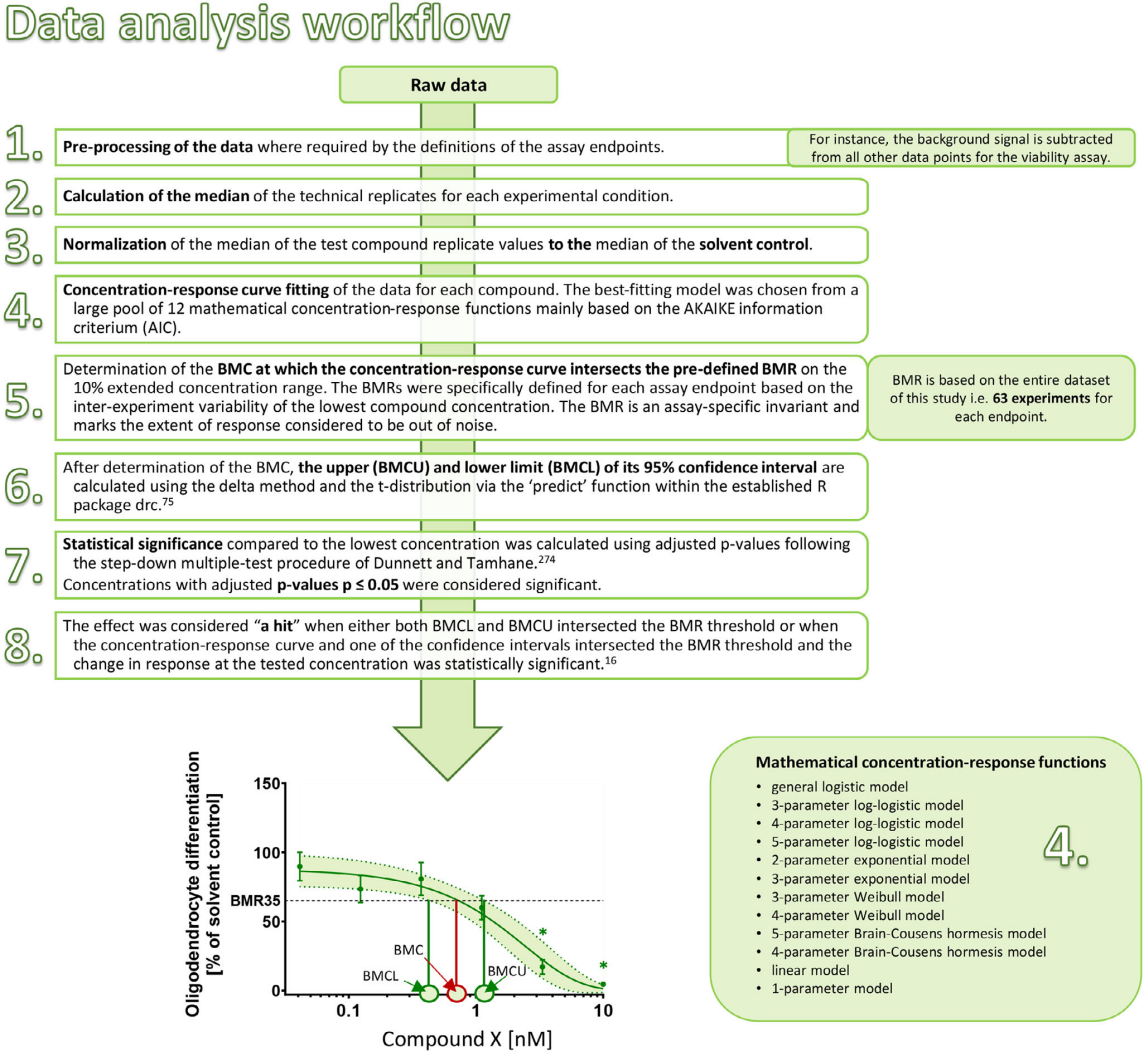
Data analysis strategy carried out in the CRStats biostatistical tool. Sources used in the figure: R package [83], procedure of Dunnet and Tamhane [285], hit classification [19]. Abbreviations: BMC = benchmark concentration, BMR = benchmark response, BMCU/L = upper and lower confidence interval of the BMC.

Concentration–response analysis from the experimental data is performed with CRStats, a biostatistical tool for concentration–response analysis (https://crstats.github.io/;github.com/iuf‐duesseldorf/koch‐lab‐CRStats). CRStats is a Shiny‐based application developed from the R package drc [83] in collaboration with biostatisticians at the IUF and Brunel University London. The tool provides an interactive environment for nonlinear regression, model comparisons, and concentration–response data management, ensuring data accessibility in line with FAIR principles. The implementation is based on the developed and established workflow [19, 38, 84] and includes the steps and outputs listed in Figure 2. In short, it includes a pre‐processing of data as required, calculation of the median of technical replicates per condition, normalization of treatment medians to solvent controls, renormalization where appropriate [86, 87], calculation of the mean of biological replicates, curve fitting using best‐fit model selection based on the Akaike information criterion (AIC), and determination of the BMC corresponding to a pre‐defined BMR as well as 95% confidence interval (BMCL, BMCU) around the BMC [88]. The graphs and overview figures were created in GraphPad Prism 10 and Adobe Illustrator 2023 software. The CRStats files as well as the raw data are available in the Biostudies repository complying with the FAIR principles of data sharing [89].

### 2.6 Physiological Map

A physiological map focused on oligodendrocyte differentiation was constructed using a bottom‐up approach, following the Disease Maps workflow [44, 46, 47]. The literature curation process for constructing the physiological map of oligodendrocyte development followed a targeted, manual, non‐systematic review of primary research articles identified via PubMed. The included publications were not quantitatively scored for relevance; rather, inclusion decisions were based on expert evaluation by the authors, guided by defined biological criteria aligned with DNT relevance. To ensure the representation of human‐relevant physiological processes, only original research publications employing non‐animal, human sample‐derived methods (human cell lines, human fetal brain tissue) were considered eligible for inclusion. Inclusion criteria required the use of healthy human cells derived from the central nervous system (CNS), specifically the brain. Studies involving spinal cord tissue, peripheral nervous system, retinal or other specialized CNS cells were excluded. Only data from non‐malignant, healthy CNS cell types were considered. To ensure developmental relevance, we included only studies focused on developmental stages beyond neural tube closure (which is addressed in a separate physiological map) [50]. and excluded adult cell types, even if these retained proliferative capacity (e.g., adult progenitors). Mechanistic relationships extracted from the curated literature were visually depicted using the Systems Biology Graphical Notation [90]. in the CellDesigner software [91]. Every entity on the map was systematically annotated with specific identifiers, including HUGO Gene Nomenclature Committee symbols for genes, RNAs, and proteins, UniProt IDs for proteins, ChEBI IDs for chemicals and metabolites, Gene Ontology IDs for phenotypes, and Cell Ontology IDs for cell types. To further enhance the map's transparency and accessibility, each entity was also linked to the respective PubMed IDs or DOIs of the literature sources where the relationships were mentioned. The completed physiological map has been made accessible and interactive through the MINERVA platform [92], providing a powerful tool for visualization and data exploration. To reduce the impact of bias in the construction of the physiological map, we employed several precautionary measures. First, the curation process was conducted by domain experts in DNT, ensuring that inclusion decisions were biologically grounded and consistent with human developmental neurobiology. Second, multiple rounds of expert review were conducted during the construction and annotation of the map, with feedback‐driven revisions enabled through the interactive and version‐controlled MINERVA platform. The map is intentionally designed as a living document, subject to continuous updates as new data emerges. This iterative approach—combined with open access and transparent annotation—helps mitigate static or outdated interpretations.

### 2.7 Use of AI Tools

Language editing support was provided using an AI‐based writing assistant (ChatGPT, Grammarly), which was used to improve clarity, shorten text passages, and refine phrasing; no content or data interpretation was generated by AI.

## 3 Results

### 3.1 Pathophysiological Applicability Domain of the Neurosphere Assay

**FIGURE 3:**
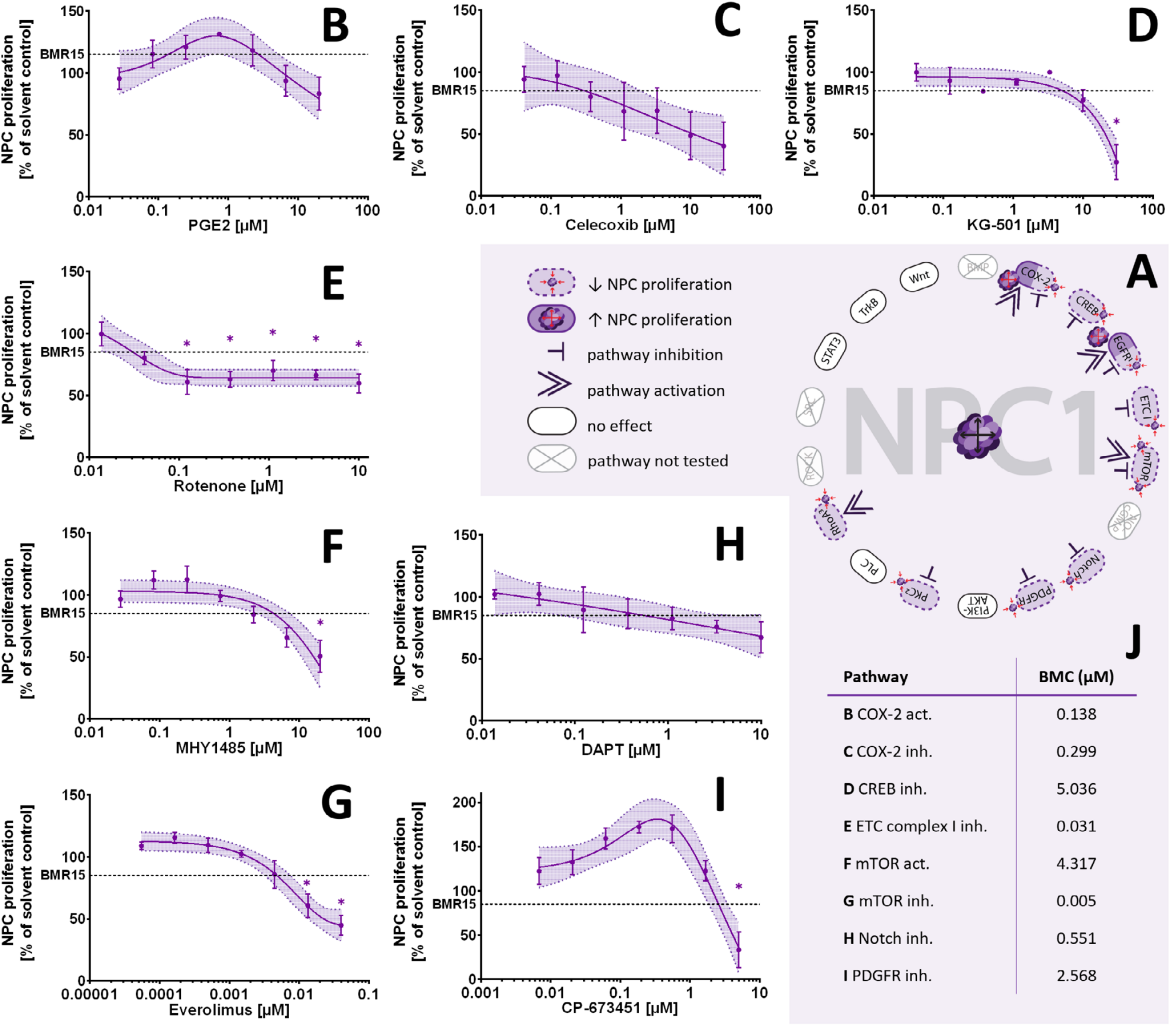
Pathway‐related mechanistic tool compounds regulate human neural progenitor cell (hNPC) proliferation. The NPC1 assay assesses NPC proliferation. Primary hNPCs were exposed for 3 days to increasing concentrations of activators and inhibitors of the selected signaling pathways in a proliferation medium containing growth factors EGF and FGF. Proliferation was assessed by BrdU incorporation into the DNA and shown as a percentage compared to the solvent control. **A** Nine signaling pathways (COX‐2, CREB, EGFR, ETC complex I, mTOR, Notch, PDGFR, PKC, and RhoA) regulated the NPC proliferation, four pathways were not assessed (BMP, NO‐cGMP, ROCK, SRC), and five (PI3K‐AKT, PLC, STAT3, TrkB, and Wnt) had no effect on the endpoint. **B** PGE2–COX‐2 activator increased NPC proliferation following a non‐monotonic U‐shaped curve compared to the solvent control. **C** Celecoxib–COX‐2 inhibitor, **D** KG‐501–CREB inhibitor, **E** Rotenone–ETC complex I, **F** MHY1485–mTOR activator, **G** Everolimus–mTOR inhibitor, **H** DAPT–Notch inhibitor, and **I** CP‐673451–PDGFR inhibitor all dose‐dependently reduced NPC proliferation compared to the solvent control except for CP‐673451 which followed a non‐monotonic U‐shaped curve. **J** Benchmark concentrations (BMCs) which caused a 15% reduction or induction in NPC proliferation according to the benchmark response (BMR15) specific for the NPC1 assay. Data are presented as mean of 3 independent experiments (*N* = 3; all experiments have been conducted in *n* = 5; in exceptional cases *n* = 3–4) ± SEM with dotted lines representing the lower and upper limit confidence intervals. BMCUs (upper) and BMCLs (lower) confidence intervals for all the compounds are listed in Table S2. *Significant (*p*‐value ≤ 0.05) compared to the respective solvent control calculated using the step‐down multiple test procedure of Dunnett and Tamhane. No‐effect results are shown in the Supporting Information File. Sources for results obtained in our previous publications for the following pathways: EGFR [24], PKC [38], RhoA [19]. Results from previously published studies are included only in the overview illustration (panel A) for completeness of pathway coverage; concentration–response graphs and BMC analyses shown in the remaining panels correspond exclusively to experiments performed in the present study. **Abbreviations**: act. = activation, inh. = inhibition, BMP = bone morphogenetic protein, COX‐2 = Cyclooxygenase‐2, CREB = cAMP response element‐binding protein, EGFR = epidermal growth factor receptor, ETC I = mitochondrial electron transport chain complex 1, mTOR = mammalian target of rapamycin, NO‐cGMP = Nitric oxide–cyclic guanosine 3′,5′‐monophosphate, PDGF = Platelet‐derived growth factor, PI3K = phosphatidylinositide 3‐kinase, PKC = protein kinase C, PLC = phospholipase C, RhoA = Ras homolog family member A, ROCK = Rho‐associated protein kinase, STAT3 = Signal transducer and activator of transcription 3, TrkB = tropomyosin receptor kinase B.

**FIGURE 4:**
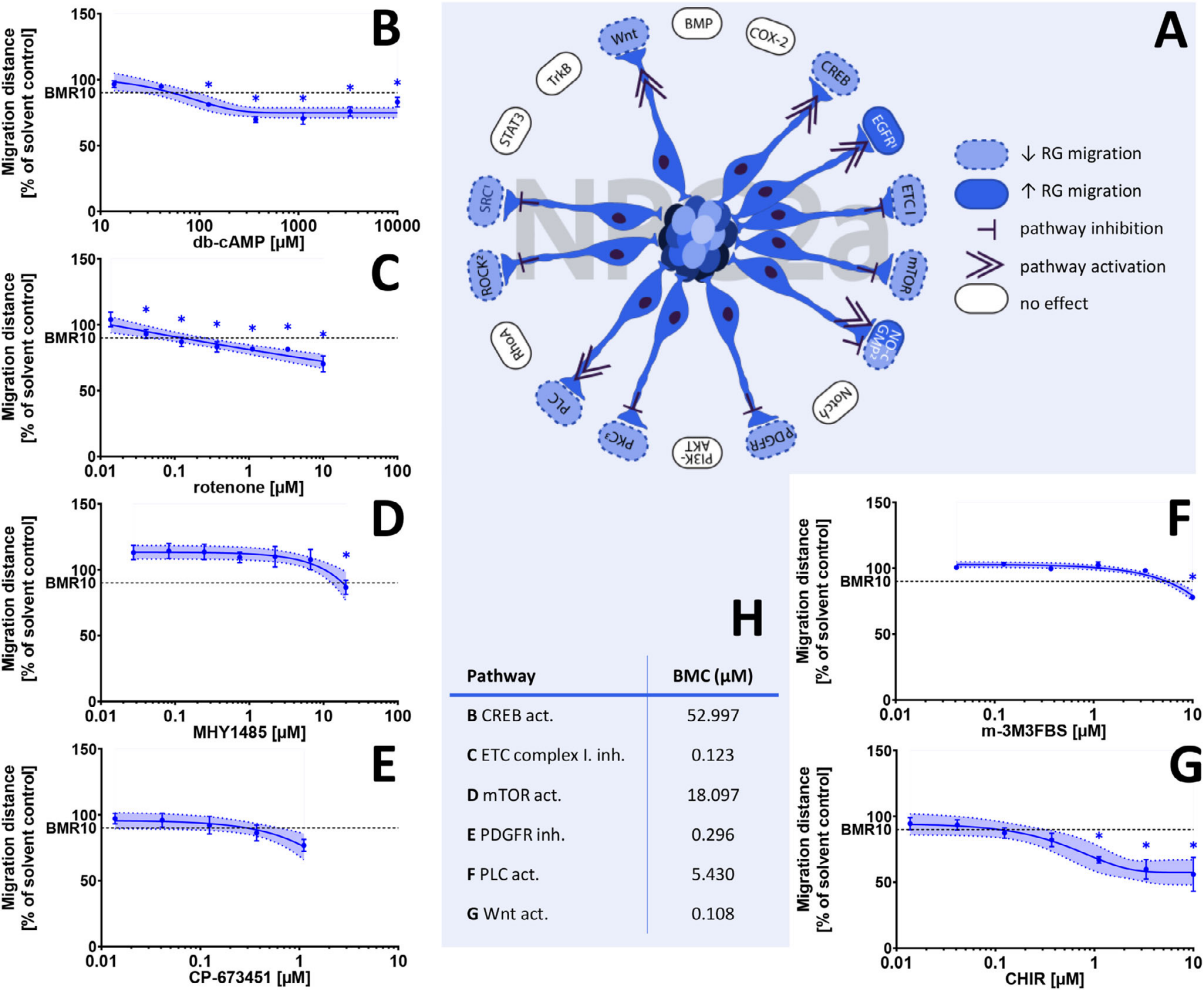
Pathway‐related mechanistic tool compounds regulate radial glia (RG) migration. The NPC2a assay assesses RG migration. Primary hNPCs were exposed for 5 days to increasing concentrations of activators and inhibitors of the selected signaling pathways in a differentiation medium without growth factors on PDL‐laminin‐coated plates. RG migration was assessed by defining the area of Hoechst33258‐stained nuclei of the radially migrated cells out of the sphere core as a percentage compared to the solvent control migration area. **A** Eleven signaling pathways (CREB, EGFR, ETC complex I, mTOR, NO‐cGMP, PDGFR, PKC, PLC, ROCK, SRC, and Wnt) regulated the RG migration and seven (BMP, COX‐2, Notch, PI3K‐AKT, RhoA, STAT3, and TrkB) had no effect on the endpoint. **B** db‐cAMP–CREB activator, **C** rotenone–ETC complex I inhibitor, **D** MHY1485–mTOR activator, **E** CP‐673451–PDGFR inhibitor, **F** m‐3M3FBS–PLC activator, and **G** CHIR99021–Wnt activator dose‐dependently decreased RG migration compared to the solvent control. **H** Benchmark concentrations (BMCs) which caused a 10% reduction in RG migration according to the benchmark response (BMR10) specific for the NPC2a assay. Data are presented as mean of 3–4 independent experiments (*N* = 3 except for *N* = 4 in the case of CP‐673451 and MHY1485; all experiments have been conducted in *n* = 5; in exceptional cases *n* = 3–4) ± SEM with dotted lines representing the lower and upper limit confidence intervals. BMCUs (upper) and BMCLs (lower) confidence intervals for all the compounds are listed in Table S2. *Significant (*p*‐value ≤ 0.05) compared to the respective solvent control calculated using the step‐down multiple test procedure of Dunnett and Tamhane. No‐effect results are shown in the Supporting Information File. Sources for results obtained in our previous publications for the following pathways: EGFR [24], NO‐cGMP [94], PKC [38], ROCK [94], and SRC [24]. Results from previously published studies are included only in the overview illustration (panel A) for completeness of pathway coverage; concentration–response graphs and BMC analyses shown in the remaining panels correspond exclusively to experiments performed in the present study. **Abbreviations**: act. = activation, inh. = inhibition, BMP = bone morphogenetic protein, COX‐2 = Cyclooxygenase‐2, CREB = cAMP response element‐binding protein, EGFR = epidermal growth factor receptor, ETC I = mitochondrial electron transport chain complex 1, mTOR = mammalian target of rapamycin, NO‐cGMP = Nitric oxide–cyclic guanosine 3′,5′‐monophosphate, PDGF = Platelet‐derived growth factor, PI3K = phosphatidylinositide 3‐kinase, PKC = protein kinase C, PLC = phospholipase C, RhoA = Ras homolog family member A, ROCK = Rho‐associated protein kinase, STAT3 = Signal transducer and activator of transcription 3, TrkB = tropomyosin receptor kinase B.

**FIGURE 5:**
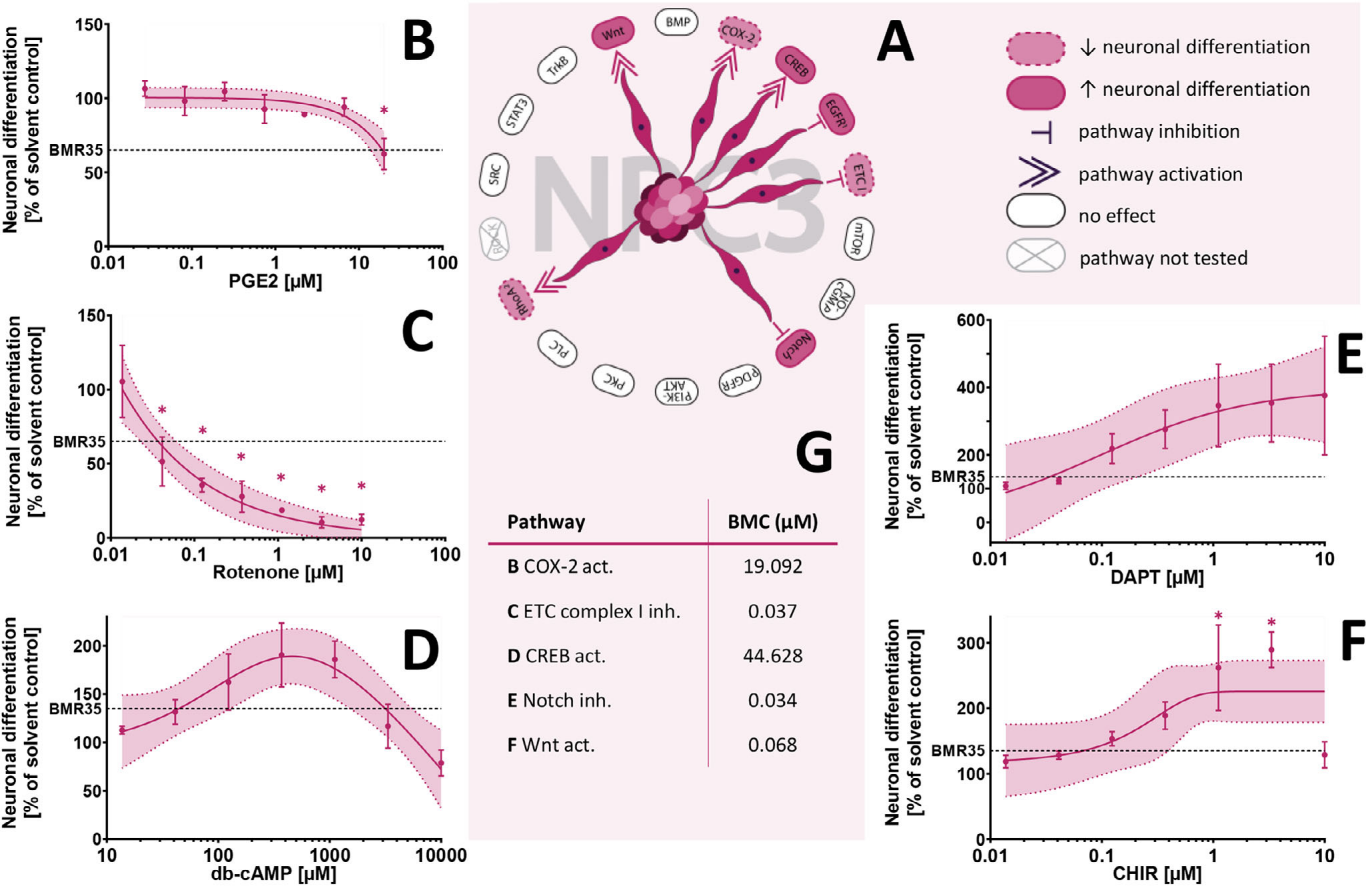
Pathway‐related mechanistic tool compounds regulate neuronal differentiation. The NPC3 assay assesses neuronal differentiation. Primary hNPCs were exposed for 5 days to increasing concentrations of activators and inhibitors of the selected signaling pathways in a differentiation medium without growth factors on PDL‐laminin‐coated plates. Neuronal differentiation was assessed as the percentage of β(III)tubulin‐positive neurons compared to the total nuclei count within the migration area. **A** Seven signaling pathways (COX‐2, CREB, EGFR, ETC complex I, Notch, RhoA, and Wnt) regulated the neuronal differentiation, one pathway was not assessed (ROCK), and nine (BMP, mTOR, NO‐cGMP, PDGFR, PI3K‐AKT, PKC, PLC, SRC, STAT3, and TrkB) had no effect on the endpoint. **B** PGE2–COX‐2 activator and **C** rotenone–ETC complex I inhibitor reduced neuronal differentiation compared to the solvent control whereas **D** db‐cAMP–CREB activator increased neuronal differentiation following a hormesis curve compared to the solvent control, and **E** DAPT–Notch inhibitor and **F** CHIR99021–Wnt activator dose‐dependently increased neuronal differentiation compared to the solvent control. **G** Benchmark concentrations (BMCs) which caused a 35% reduction or induction in neuronal differentiation according to the benchmark response (BMR35) specific for the NPC3 assay. Data are presented as mean of 3–4 independent experiments (*N* = 3 except for *N* = 4 in the case of PGE2 and DAPT; all experiments have been conducted in *n* = 5; in exceptional cases *n* = 3–4) ± SEM with dotted lines representing the lower and upper limit confidence intervals. BMCUs (upper) and BMCLs (lower) confidence intervals for all the compounds are listed in Table S2. *Significant (*p*‐value ≤ 0.05) compared to the respective solvent control calculated using the step‐down multiple test procedure of Dunnett and Tamhane. No‐effect results are shown in the Supporting Information File. Sources for results obtained in our previous publications for the following pathways: EGFR [37], RhoA [19]. Results from previously published studies are included only in the overview illustration (panel A) for completeness of pathway coverage; concentration–response graphs and BMC analyses shown in the remaining panels correspond exclusively to experiments performed in the present study. **Abbreviations**: act. = activation, inh. = inhibition, BMP = bone morphogenetic protein, COX‐2 = Cyclooxygenase‐2, CREB = cAMP response element‐binding protein, EGFR = epidermal growth factor receptor, ETC I = mitochondrial electron transport chain complex 1, mTOR = mammalian target of rapamycin, NO‐cGMP = Nitric oxide–cyclic guanosine 3′,5′‐monophosphate, PDGF = Platelet‐derived growth factor, PI3K = phosphatidylinositide 3‐kinase, PKC = protein kinase C, PLC = phospholipase C, RhoA = Ras homolog family member A, ROCK = Rho‐associated protein kinase, STAT3 = Signal transducer and activator of transcription 3, TrkB = tropomyosin receptor kinase B.

**FIGURE 6:**
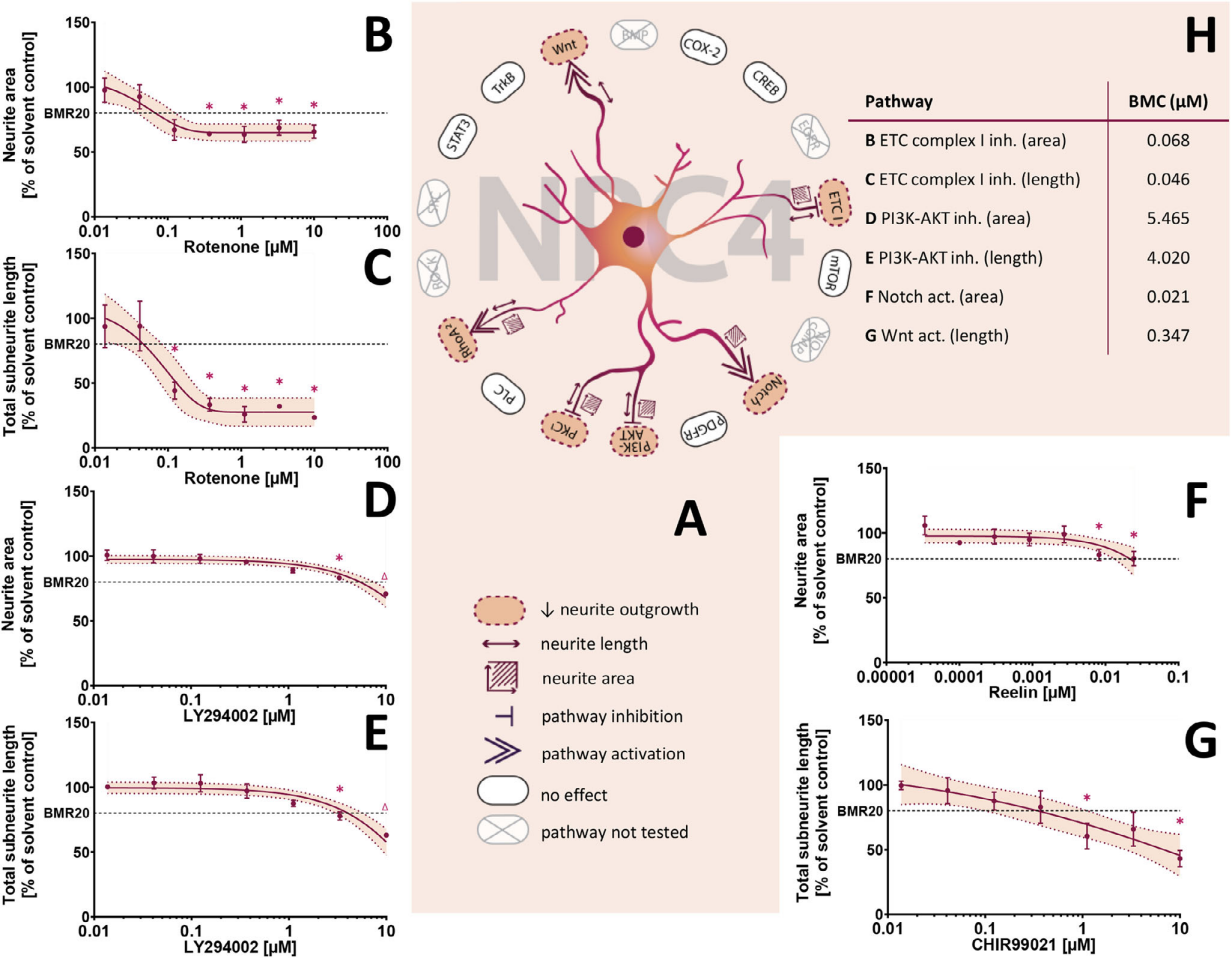
Pathway‐related mechanistic tool compounds regulate neurite outgrowth. The NPC4 assay assesses neurite outgrowth. Primary hNPCs were exposed for 5 days to increasing concentrations of activators and inhibitors of the selected signaling pathways in a differentiation medium without growth factors on PDL‐laminin‐coated plates. Neurite outgrowth was assessed as neurite length (µm) and neurite area (pixel) of all β(III)tubulin‐positive neurons. **A** Six signaling pathways (ETC complex I, Notch, PI3K‐AKT, PKC, RhoA, and Wnt) regulated the neurite outgrowth, five pathways were not assessed (BMP, EGFR, NO‐cGMP, ROCK, and SRC), and seven (COX‐2, CREB, mTOR, PDGFR, PLC, STAT3, and TrkB) had no effect on the endpoint. **B,C** rotenone–ETC complex I inhibitor and **D,E** LY294002–PI3K‐AKT inhibitor dose‐dependently reduced both neurite area (**B,D**) and length (**C,E**) compared to the solvent control. **F** Reelin–Notch activator dose‐dependently decreased neurite area compared to the solvent control. **G** CHIR99021–Wnt activator dose‐dependently reduced neurite length. **H** Benchmark concentrations (BMCs) which caused a 20% reduction in neurite outgrowth according to the benchmark response (BMR20) specific for the NPC4 assay. Data are presented as mean of 3 independent experiments (*N* = 3; all experiments have been conducted in *n* = 5; in exceptional cases *n* = 3–4) ± SEM with dotted lines representing the lower and upper limit confidence intervals. BMCUs (upper) and BMCLs (lower) confidence intervals for all the compounds are listed in Table S2. *significant (*p*‐value ≤ 0.05) compared to the respective solvent control calculated using the step‐down multiple test procedure of Dunnett and Tamhane. No‐effect results are shown in the Supporting Information File. Sources for results obtained in our previous publications for the following pathways: PKC [38], and RhoA [19]. Results from previously published studies are included only in the overview illustration (panel A) for completeness of pathway coverage; concentration–response graphs and BMC analyses shown in the remaining panels correspond exclusively to experiments performed in the present study. **Abbreviations**: act. = activation, inh. = inhibition, BMP = bone morphogenetic protein, COX‐2 = Cyclooxygenase‐2, CREB = cAMP response element‐binding protein, EGFR = epidermal growth factor receptor, ETC I = mitochondrial electron transport chain complex 1, mTOR = mammalian target of rapamycin, NO‐cGMP = Nitric oxide–cyclic guanosine 3′,5′‐monophosphate, PDGF = Platelet‐derived growth factor, PI3K = phosphatidylinositide 3‐kinase, PKC = protein kinase C, PLC = phospholipase C, RhoA = Ras homolog family member A, ROCK = Rho‐associated protein kinase, STAT3 = Signal transducer and activator of transcription 3, TrkB = tropomyosin receptor kinase B.

**FIGURE 7:**
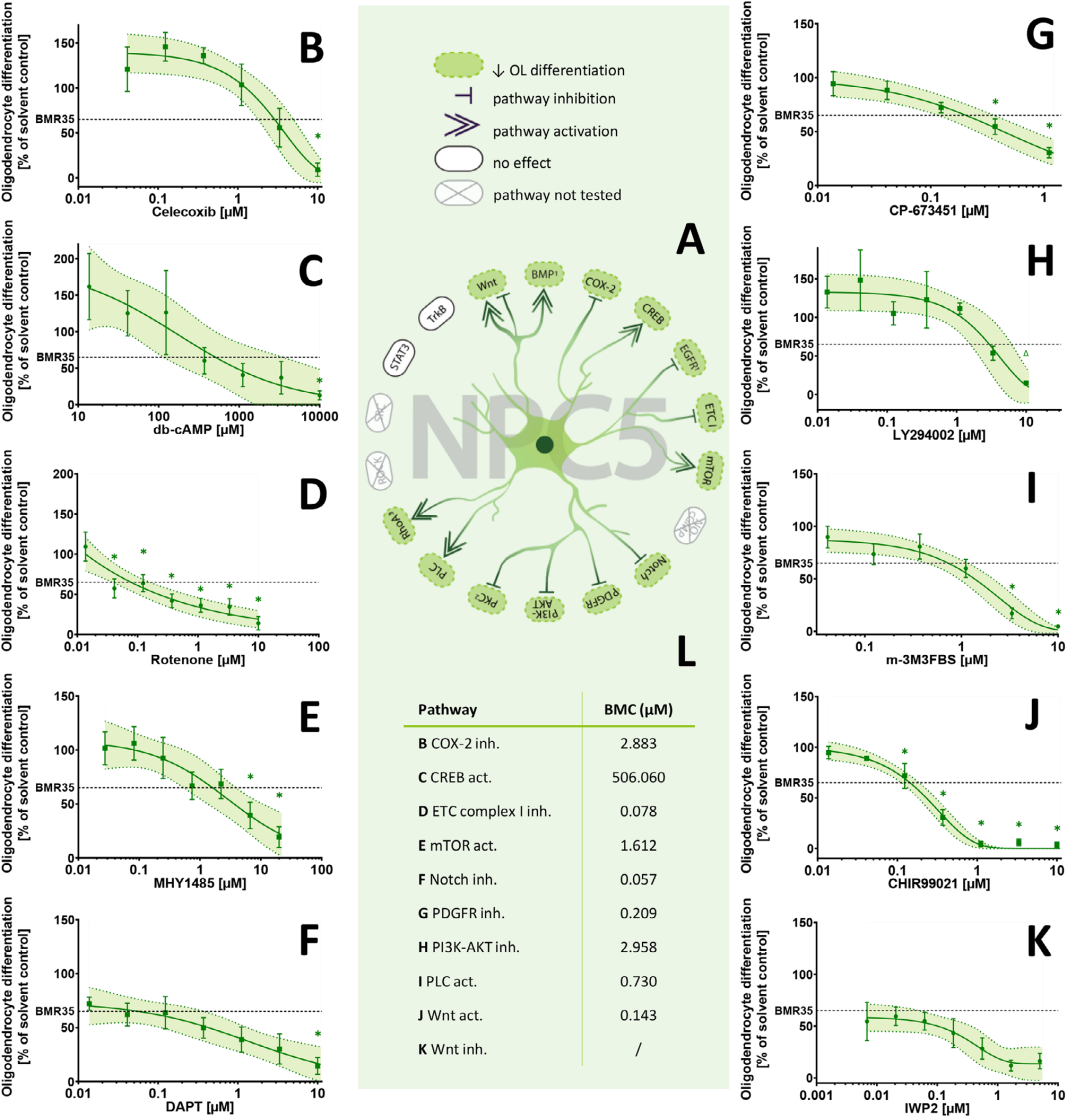
Pathway‐related mechanistic tool compounds regulate oligodendrocyte differentiation (OL). The NPC5 assay assesses OL differentiation. Primary hNPCs were exposed for 5 days to increasing concentrations of activators and inhibitors of the selected signaling pathways in a differentiation medium without growth factors on PDL‐laminin‐coated plates. OL differentiation was assessed as the percentage of O4‐positive OLs compared to the total nuclei count within the migration area. **A** Thirteen signaling pathways (BMP, COX‐2, CREB, EGFR, ETC complex I, mTOR, Notch, PDGFR, PI3K‐AKT, PKC, PLC, RhoA, and Wnt) regulated the OL differentiation, three pathways were not assessed (NO‐cGMP, ROCK, SRC), and two (STAT3, TrkB) did not affect the endpoint. **B** Celecoxib–COX‐2 inhibitor, **C** db‐cAMP–CREB activator, **D** rotenone–ETC complex I inhibitor, **E** MHY1485–mTOR activator, **F** DAPT–Notch inhibitor, **G** CP‐673451–PDGFR inhibitor, **H** LY294002–PI3K‐AKT inhibitor, **I** m‐3M3FBS–PLC activator, **J** CHIR99021–Wnt activator, and **K** IWP2–Wnt inhibitor all dose‐dependently reduced oligodendrocyte differentiation compared to the solvent control. **L** Benchmark concentrations (BMCs) which caused a 35% reduction in OL differentiation according to the benchmark response (BMR35) specific for the NPC5 assay. No BMC is shown for IWP2 (**K**) due to the lowest concentration being below the BMR thus not allowing for BMC calculation. Data are presented as mean of 3–4 independent experiments (*N* = 3 except for *N* = 4 in the case of MHY1485, DAPT, and CP‐673451; all experiments have been conducted in *n* = 5; in exceptional cases *n* = 3–4) ± SEM with dotted lines representing the lower and upper limit confidence intervals. BMCUs (upper) and BMCLs (lower) confidence intervals for all the compounds are listed in Table S2. *Significant (*p*‐value ≤ 0.05) compared to the respective solvent control calculated using the step‐down multiple test procedure of Dunnett and Tamhane. ∆*N* = 2 (**H**). No‐effect results are shown in the Supporting Information File. Sources for results obtained in our previous publication for the following pathways: BMP [37], EGFR [37], PKC [38], RhoA [19]. Results from previously published studies are included only in the overview illustration (panel A) for completeness of pathway coverage; concentration–response graphs and BMC analyses shown in the remaining panels correspond exclusively to experiments performed in the present study. Abbreviations: act. = activation, inh. = inhibition, BMP = bone morphogenetic protein, COX‐2 = Cyclooxygenase‐2, CREB = cAMP response element‐binding protein, EGFR = epidermal growth factor receptor, ETC I = mitochondrial electron transport chain complex 1, mTOR = mammalian target of rapamycin, NO‐cGMP = Nitric oxide–cyclic guanosine 3′,5′‐monophosphate, PDGF = Platelet‐derived growth factor, PI3K = phosphatidylinositide 3‐kinase, PKC = protein kinase C, PLC = phospholipase C, RhoA = Ras homolog family member A, ROCK = Rho‐associated protein kinase, STAT3 = Signal transducer and activator of transcription 3, TrkB = tropomyosin receptor kinase B.

In an effort to map the signaling pathways involved in NDDs to the neurodevelopmental key events of the Neurosphere Assay, we exposed primary fetal hNPCs under proliferating (NPC1 assay) and differentiating (NPC2–5) conditions to increasing concentrations of specific activators and inhibitors of selected 11 signaling pathways complemented by 7 additional pathways from our previous studies. Concentration–response curves of individual substances with effects on studied endpoints are shown in Figures 3, 4, 5, 6, 7, whereas no‐effect results are available in Supporting Information file 1. To ensure that only pathway‐specific results were observed, cytotoxic concentrations were excluded from the analyses. Graphs showing the accompanying viability (mitochondrial activity) and nuclei number results are also presented in Supporting Information file 1. In order to visualize all so far investigated pathways guiding the individual KNDPs of the Neurosphere Assay, we included the results of our previous studies concerning the 7 additional pathways in the overview Figures 3, 4, 5, 6, 7 and Figure S1. Figures 3, 4, 5, 6, 7 provide respective summaries of how each KNDP is affected by mechanistic tool compounds, including concentration–response graphs and tables listing the corresponding BMC values.

Concentration–response data revealed that NPC proliferation increased following treatment with compounds targeting Cyclooxygenase‐2 (COX‐2) activation (^a^) by PGE2 and PDGFR inhibition (^I^) by CP‐673451 and decreased following exposure to compounds associated with inhibition or activation of several pathways, including COX‐2^I^ (celecoxib), db‐cAMP response element‐binding protein (CREB)^I^ (KG‐501), mitochondrial electron transport chain complex 1 (ETC complex I)^I^ (rotenone), mammalian target of rapamycin (mTOR)^a^, (MHY1485), mTOR^i^ (everolimus), Notch^i^ (DAPT), and Platelet‐derived growth factor (PDGFR)^i^ (CP‐673451; Figure 3B‐I). Thereby, PGE2 and CP‐673451 exposure resulted in a non‐monotonic U‐shaped concentration–response curve (Figure 3B‐I). For prostaglandins, U‐shaped responses have been reported previously [93]. (Figure 3B) while, to the best of our knowledge, this is a novel finding for the PDGFR pathway. All other treatments follow monotonic concentration–response relationships. From these curves, BMC_15_ values were calculated, i.e., 0.138 µM (PGE2), 0.299 µM (Celecoxib), 5.036 µM (KG‐501), 0.031 µM (rotenone), 4.317 µM (MHY1485), 0.005 µM (everolimus), 0.551 µM (DAPT), and 2.568 µM (CP‐673451). Uncertainties of the curves were described by confidence bands accompanying the curves. Data on the additional pathways indicated in Figure 3A, i.e., epidermal growth factor receptor (EGFR)^ai^ [24], protein kinase C (PKC)^i^ [38], and Ras homolog family member A (RhoA)^i^ [19] were taken from previous publications.

RG migration was found to be a highly regulated process consistent with the following pathway modulations: CREB^a^ (db‐cAMP), ETC complex I^i^ (rotenone), mTOR^a^ (MHY1485), PDGFR^i^ (CP‐673451), phospholipase C (PLC)^a^ (m‐3M3FBS), and Wnt^a^ (CHIR99021) with BMC_10_ values 52.997 µM, 0.123 µM, 18.097 µM, 0.296 µM, 5.430 µM, and 0.108 µM, respectively (Figure 4B–G), in addition to our previous studies on the pathways EGFR [24], Nitric oxide–cyclic guanosine 3′,5′‐monophosphate (NO‐cGMP) [94], PKC [38], Rho‐associated protein kinase (ROCK) [94], and SRC [24, 95] (Figure 4A). In contrast, neuronal (NPC2b) and oligodendrocyte migration (NPC2c) were the least regulated KNDPs of the Neurosphere Assay with the first not affected by any of the tested molecules (Supporting Information File) and the latter solely inhibited by narciclasine associated with RhoA^i^ [19] (Figure S1).

Neuronal differentiation (BMR 35%) reduction was indicative of the following pathways’ involvement: COX‐2^a^ (PGE2; 19.092 µM) and ETC complex I^i^ (rotenone; 0.037 µM) and induction was associated with modulation of CREB^a^ (db‐cAMP; 44.628 µM), Notch^i^ (DAPT; 0.034 µM), and Wnt^a^ (CHIR99021; 0.068 µM). Previous studies also identified compounds modulating EGFR [37] and RhoA [19] to increase and decrease neuronal differentiation, respectively (Figure 5).

Differentiated neurons in the neurosphere migration area form neurites whose lengths and areas were assessed. This neurite outgrowth was reduced (BMR20) by compounds modulating the ETC complex I^i^ (rotenone; neurite length and neurite area 0.046 µM and 0.068 µM, respectively), Notch^a^ (reelin; neurite area 0.021 µM), phosphatidylinositide 3‐kinase (PI3K)‐AKT^i^ (LY294002; neurite length 4.020 µM, neurite area 5.465 µM), and Wnt^a^ (CHIR99021; neurite length 0.347 µM; Figure 6B–G) adding to the historical neurite outgrowth inhibition of PKC [38], and RhoA [19] (Figure 6A). No pathway modulation was associated with increased neurite outgrowth.

Finally, the most regulated KNDP within the Neurosphere Assay was oligodendrocyte differentiation, whose inhibition (BMR35) was associated with COX‐2^i^ (celecoxib; 2.883 µM), CREB^a^ (db‐cAMP; 506.060 µM), ETC complex I^i^ (rotenone; 0.078 µM), mTOR^a^ (MHY1485; 1.612 µM), Notch^i^ (DAPT; 0.057 µM), PDGFR^i^ (CP‐673451; 0.209 µM), and PI3K‐AKT^i^ (LY294002; 2.958 µM; Figure 7B–K), PLC^a^ (m‐3M3FBS; 0.730 µM), Wnt^a^ (CHIR99021; 0.143 µM), and Wnt^i^ (IWP2; BMC unavailable due to shifted dose–response curve, which most likely resulted from data limitations at lower concentrations). In addition, inhibition of oligodendrocyte differentiation by compounds targeting bone morphogenetic protein (BMP) [37], EGFR [37], PKC [38], and RhoA [19] were reported in our previous studies (Figure 7A).

**FIGURE 8:**
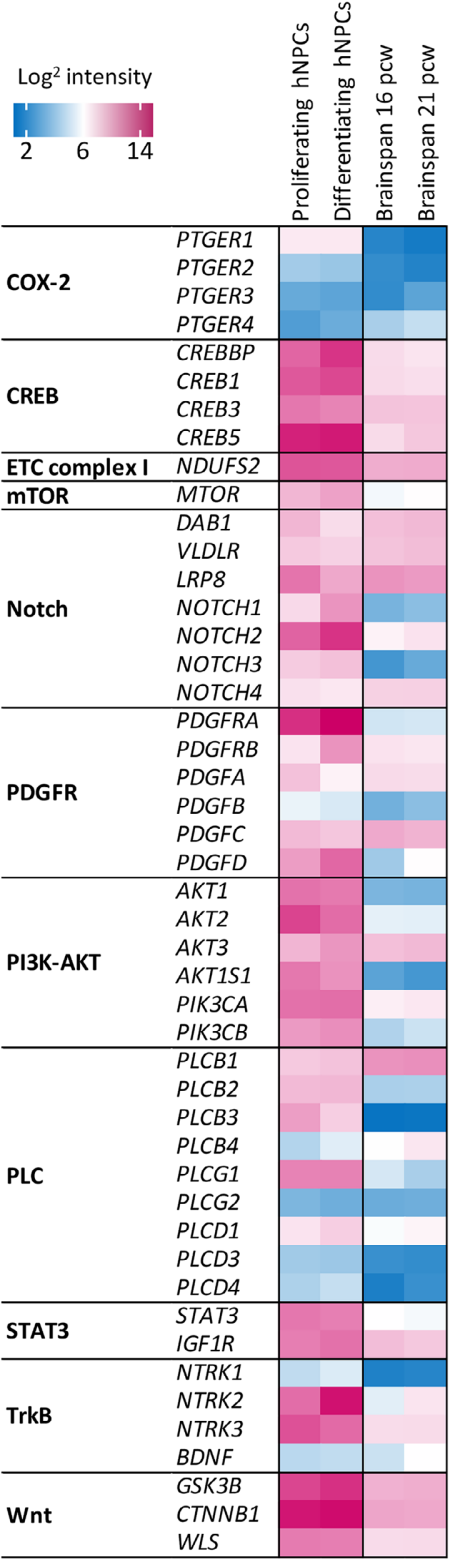
Expression of mRNA coding for proteins involved in the signaling cascades investigated in this manuscript. Transcriptomic profiling using microarray analyses of proliferating and differentiating (60 h) hNPCs was performed in our previously published work [80] and was complemented with microarray results of fetal brain samples (16 and 21 postconceptional weeks) available at the Brainspan database brainspan.org [81]. Genes are defined as likely to be expressed (present) or likely to be not expressed (absent) based on criteria adapted from Kang et al. [203]. Thereby if median log2 intensity value in samples is ≥6 = present (red scale), ≤6 = absent (blue scale). **Abbreviations**: hNPCs = human neuroprogenitor cells; pcw = postconceptional week, COX‐2 = Cyclooxygenase‐2, CREB = cAMP response element‐binding protein, ETC I = mitochondrial electron transport chain complex 1, mTOR = mammalian target of rapamycin, PDGF = Platelet‐derived growth factor, PI3K = phosphatidylinositide 3‐kinase, PLC = phospholipase C, STAT3 = Signal transducer and activator of transcription 3, TrkB = tropomyosin receptor kinase B.

RhoA and ETC complex I pathway mechanistic tool compounds affected the most endpoints, while signal transducer and activator of transcription 3 (STAT3) and tropomyosin receptor kinase B (TrkB) were outside of the applicability domain of the Neurosphere Assay as they did not lead to any endpoint modulation. It is to note that due to the historic study designs, not all previously generated data covered all neurosphere endpoints. Finally, the expression of genes coding for important proteins involved in the assessed signaling pathways is shown in Figure 8. It demonstrates expression analyzed in proliferating and differentiating hNPCs published in our previous study [80] and is complemented with expression measured in fetal (16 and 21 pcw) brain samples publicly available in the BrainSpan database for in vitro‐in vivo comparison. The heatmap displays highly expressed genes in pink and low‐expressed genes in blue. The proliferating and differentiating in vitro cultures show very comparable patterns, with genes staying either lowly or highly expressed regardless of the culturing condition. The same applies to the expression in fetal brain samples, although a few genes (*MTOR*, *NOTCH1‐3*, *PDGFB*,*C*,*D*, *PIK3CB*, *PLCB4*, *PLCD1*, *NTRK2*, and *BDNF*) are slightly more expressed at 21 pcw than at 16 pcw. As expected, the in vitro cultures and fetal brain samples show comparable patterns, highlighting the similarity of the in vitro model with human physiology at a respective developmental stage that the in vitro model represents. The exception is *PTGER1*, *MTOR*, *NOTCH1,3*, *PDGFRA*, *PDGFD*, *AKT1‐2*, *AKT1S1*, *PIK3CB*, *PLCB2‐3*, *PLCG1*, and *STAT3*, which are expressed in the in vitro cultures but not in the fetal brain samples. Most importantly, all genes highly expressed in the fetal brain samples are also expressed in the in vitro cultures, confirming that the in vitro human NPC model preserves the receptor expression profile of human fetal cortex tissue. Finally, the low expression of the *BDNF* gene both in the in vitro cultures and fetal brain samples demonstrates low maturity of the modeled fetal developmental stage and is probably the reason why the TrkB pathway is outside of the biological applicability domain of the Neurosphere Assay, as will be discussed further in the Discussion section.

### 3.2 Physiological Map of Oligodendrocyte Differentiation

**FIGURE 9:**
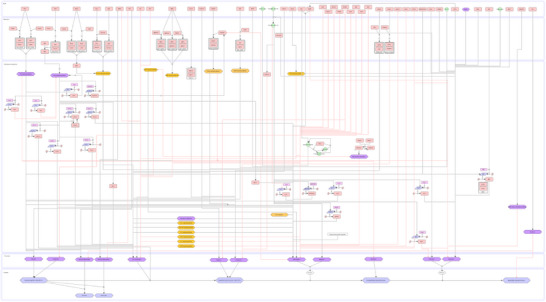
Physiological map of the oligodendrocyte development integrating curated literature knowledge represented using the Systems Biology Graphical Notation. The map is annotated with literature sources (PubMed IDs) and ontological identifiers for each entity. Outcomes from our in vitro test battery are highlighted in orange nodes. Pink diamond shapes represent RNAs, light purple rectangles represent genes, rounded corner rectangles in salmon color represent proteins, light green circles represent simple molecules, dark purple six‐vertex diamond shapes represent biological processes, and light purple six‐vertex diamond shapes represent cell types and their developmental stages. Black edges represent positive influences and red edges represent negative influences. As the figure in the paper is a static representation, we strongly encourage readers to explore the fully interactive version on the MINERVA platform for proper navigation and detailed insights: https://ontox.elixir‐luxembourg.org/minerva/index.html?id=ONTOX_Oligodendrocyte_development.

The oligodendrocyte differentiation physiological map (Figure 9) displays the intricate process of oligodendrocyte development, encompassing crucial stages from NPC specification via oligodendrocyte progenitor cells (OPCs) to the full maturation into myelinating oligodendrocytes. A selection of human‐oriented experimental papers (Excel Table S4) served as the foundation for constructing this 100% human‐data‐based map, ensuring its relevance to human physiology. The central‐bottom part of the map (indicated by the purple differentiation node) specifically focuses on the processes influencing oligodendrocyte differentiation from OPCs to pre‐myelinating oligodendrocytes, KNDP modeled by the NPC5 assay, which play a vital role in the development of the central nervous system and can be perturbed by exogenous chemicals with a subsequent possible role in the onset and progression of NDDs.

**FIGURE 10:**
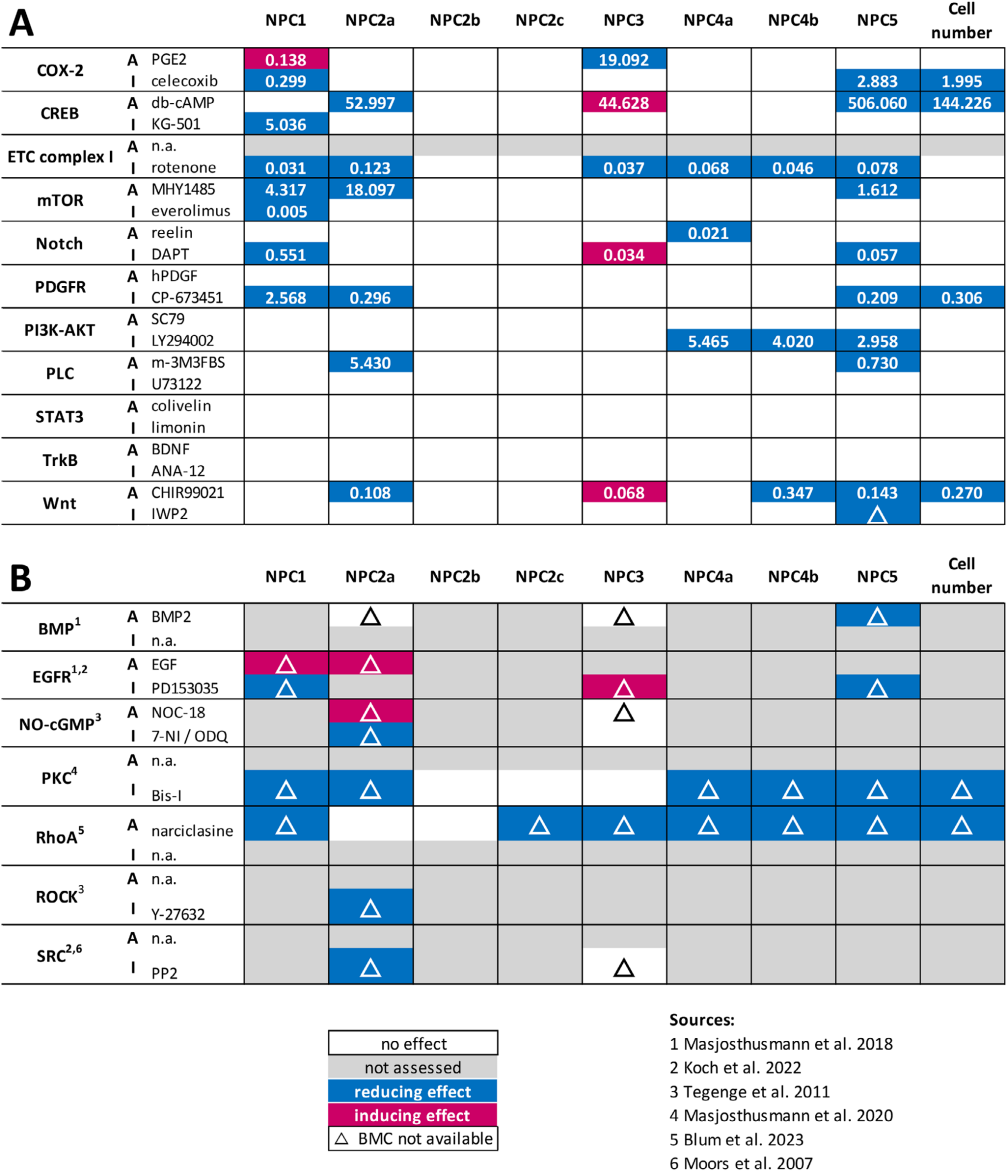
Overview of the signaling pathways regulating KNDPs (NPC1‐5) in the Neurosphere Assay. Neurosphere Assay endpoints enhanced or impaired by the pathway‐related mechanistic tool compounds are shown in pink and blue, respectively, whereas those unaffected are shown in white. Processes that were not assessed are shaded in grey. △ benchmark concentration not available (reasons include, e.g., a different benchmark response used in the previous study, only one concentration tested, shorter exposure time, etc.). Section **A** contains pathways investigated in the present study whereas **B** contains pathways investigated in our previous work. Values are indicated as benchmark concentrations in µM. **Abbreviations**: A = activator, I = inhibitor, BDNF = brain‐derived neurotrophic factor, BMC = benchmark concentration, BMP = bone morphogenetic protein, db‐cAMP = cyclic adenosine monophosphate, COX‐2 = Cyclooxygenase‐2, CREB = cAMP response element‐binding protein, EGFR = epidermal growth factor receptor, ETC I = mitochondrial electron transport chain complex 1, mTOR = mammalian target of rapamycin, NO‐cGMP = Nitric oxide–cyclic guanosine 3′,5′‐monophosphate, NPC1–NPC proliferation, NPC2a–RG migration, NPC2b–neuronal migration, NPC2c–oligodendrocyte migration, NPC3–neuronal differentiation, NPC4a–neurite area; NPC4b–neurite length, NPC5–oligodendrocyte differentiation, PDGF = Platelet‐derived growth factor, PGE2 = prostaglandin E2, PI3K=phosphatidylinositide 3‐kinase, PKC = protein kinase C, PLC = phospholipase C, RhoA = Ras homolog family member A, ROCK = Rho‐associated protein kinase, STAT3 = Signal transducer and activator of transcription 3, TrkB = tropomyosin receptor kinase B. Sources: Masjosthusmann et al. 2018 [37], Koch et al. 2022 [24], Tegenge et al. 2011 [94], Masjosthusmann et al. 2020 [38], Blum et al. 2023 [19], Moors et al. 2007 [95].

To enhance the map's relevance, all statistically significant in vitro outcomes for NPC5 summarized in Figure 10A,B were systematically integrated into the visualization, represented by nodes and connections highlighted in orange. This integration allowed for comparison and alignment of our experimental findings with the existing knowledge about oligodendrocyte differentiation. An interactive version of the map can be found at https://ontox.elixir‐luxembourg.org/minerva/index.xhtml?id=ONTOX_Oligodendrocyte_development and a comprehensive table of annotations is available in Excel Table S4.

## 4 Discussion

Our study explored the pathophysiological applicability domains of DNT IVB assays by mapping human NDD pathways to cellular KNDPs modeled in the human Neurosphere Assay, an integral part of the DNT IVB [1, 3]. KNDPs represent key links between genotypes and NDDs [96], and are due to this pathophysiological relevance perfectly suited for assessing compound effects in vitro. Presence and function of relevant neurodevelopmental disease pathways in DNT NAMs significantly expand knowledge, support interpretation of results in regulatory contexts, and increase confidence in DNT IVB assays for human health protection and hence contribute to ongoing efforts toward regulatory implementation of NAMs such as screening, prioritization, or hazard identification, as outlined in the OECD guidance document on the DNT IVB [1]; however, specific regulatory applications and criteria must ultimately be defined by regulatory authorities. Importantly, the aim of this study was not to model specific NDDs directly, but rather to determine whether signaling pathways implicated in these disorders can be functionally interrogated through KNDPs represented in the Neurosphere Assay. In this context, the pathway–disease associations serve to anchor the observed pathway–KNDP relationships in human‐relevant biology and to define the biological applicability domain of the test system.

Each assay studied evaluates a distinct KNDP, i.e., neural progenitor cell proliferation (NPC1), RG, neuronal, and oligodendrocyte migration (NPC2abc), neurite outgrowth (NPC4ab), as well as neuronal and oligodendrocyte differentiation (NPC3, NPC5, respectively). An overview of the involvements of all 18 studied signaling pathways in KNDPs modeled within the Neurosphere Assay is shown in Figures 1 and 10, whereas the human disease associations of these pathways can be drawn from Table 1. These summaries integrate the majority of data from this study, yet are supplemented by our previously published pathway analyses in the Neurosphere Assay for data comprehensiveness [19, 24, 37, 38, 94, 95]. Here in the main discussion, we focus on a novel pathway–endpoint associations generated in the present study. We will alphabetically discuss the human physiological and pathophysiological relevance of each of these novel pathways, highlighting similarities and differences with corresponding rodent processes. Additional commentaries on the seven pathways that were previously published and discussed are provided in the Supporting Information File 1. To support interpretation of pathway relevance, Figure 8 provides gene expression profiles of key pathway components in our cell model and fetal brain tissue, enabling assessment of target presence and model comparability. Lastly, we combine expert literature curation in a systems biology approach with our in vitro battery results using physiological maps to mechanistically explore pathways involved in oligodendrocyte development (NPC5) and their influence on toxicological endpoints (Figure 9). Given the increasing recognition of oligodendrocytes as critical targets of chemical exposure [24, 74, 80, 97, 98, 99] a deeper understanding of the converging mechanisms of oligodendrocyte toxicity is essential. The physiological map presented here should be interpreted as an expert‐curated, hypothesis‐generating framework intended to contextualize the observed pathway–KNDP associations and illustrate the biological applicability domain of the assay, rather than as a comprehensive or systematically validated model of oligodendrocyte development.

**Cyclooxygenase‐2** is an enzyme that catalyzes the first step in prostaglandin synthesis from arachidonic acid. It regulates neurotransmission, synaptic plasticity, dendritogenesis, and endocannabinoid signaling, subsequently shaping learning and memory functions [100, 101]. The most sensitive endpoint regulated by compounds targeting COX‐2 across KNDPs studied is NPC proliferation (NPC1; Figure 10). Our results obtained with PGE2 (a surrogate endogenous metabolite mimicking COX‐2 activation) and celecoxib (COX‐2 inhibitor) corroborate previous findings on COX‐2‐mediated progenitor proliferation observed in vitro in mouse neuroectodermal stem cells [102], in vivo in zebrafish [103], and in vivo in COX‐2 knock‐out mice [104]. It has been also reported that celecoxib, a COX‐2 inhibitor, might suppress cell proliferation via COX‐2‐independent mechanisms, such as cell cycle arrest, as reported in cancer cell lines [105, 106]. Additionally, COX‐2 is also a key modulator in neuroinflammation, contributing to neurological disorders like neonatal white matter injury and cerebral palsy, particularly in preterm infants after inflammation or hypoxic‐ischemic injury [107]. Oligodendrocytes are especially vulnerable to these injuries, which disrupt their differentiation and myelination [108]. This is mediated via COX‐2‐PGE2 signaling that is activated by reactive astrocytes as seen in human post‐mortem samples [109]. Given COX‐2's role in oligodendrocyte damage, it was unexpected that we did not observe decreased differentiation following PGE2 exposure (Figures 9A and 10A). The effects of COX‐2 are mediated by four PGE2 receptor subtypes (EP1–EP4, encoded by *PTGER1–4*), which have been associated with both neurotoxic [110, 111] and neuroprotective roles depending on receptor subtype, cell type, and developmental context [112]. All four receptors had low expression in our model at levels comparable to fetal brain human tissue at similar developmental stages (Figure 8). EP1, although higher expressed, appears to be less relevant for OPCs; its abundance in OPCs was low in the study from Carlson et al., and it does not seem to contribute to oligodendrocyte excitotoxicity, despite playing a significant role in neuronal excitotoxicity [110]. EP2 is commonly associated with neuroprotective effects against excitotoxicity, while EP3 has been linked to neurotoxicity, potentially through decreased intracellular cAMP or increased calcium signaling [110] as well as EP4 [113]. These findings suggest that receptor‐specific signaling play a context‐dependent role in modulating oligodendrocyte outcomes. The absence of an adverse effect of PGE2 in our system might be also explained by the maturity stage of the oligodendrocytes. The team around Shiow et al. observed that PGE2 decreased rat and mouse OPC maturation but not OPC proliferation and survival in vitro [109]. The OPC maturation KNPD that directly follows the differentiation of NPCs into OPCs is dependent on triiodothyronine (T3) hormone maturation treatment. The T3‐mediated OPC maturation is however not assessed in the NPC5 assay but in the NPC6 assay [24, 80] that is not part of this manuscript and its biological applicability domain warrants further investigation. Finally, the negligible expression of PGE2 receptors and the observed neuronal differentiation‐reducing effects in higher concentrations (19 µM) might indicate receptor‐independent effects for PGE2 comparable to what we observed in our previous study on nuclear hormone receptors [79].

**CREB** belongs to a large group of transcription factors able to dimerize, interact with DNA and regulate gene expression. It is located in the nucleus and can be activated by db‐cAMP, CaMK, and other kinases. As a nuclear transcription factor, CREB initiates transcription of target genes like *BDNF* [114], *FOS* [115], and *TH* [116] further regulating dopamine signaling, neurogenesis, metabolism, proliferation and survival [117]. CREB is crucial during prenatal development and is highly expressed in both hNPCs and fetal brain samples (Figure 8) aligning with the lethal prenatal knock‐out phenotype of this gene [118]. We observed reduced RG migration (NPC2a), concordant with CREB's known regulation of cytoskeletal dynamics [119, 120], as well as oligodendrocyte differentiation (NPC5), and enhanced neuronal differentiation (NPC3) after treatment with compounds leading to CREB activation. Similar neuronal differentiation and also maturation‐enhancing findings were observed in rat primary NPCs [121]. On the other hand, our findings on decreased oligodendrocyte differentiation contrast with previous studies (reviewed in [122]), where db‐cAMP (around 1 mM) generally promotes differentiation in primary rat in vitro cultures [123, 124] and is commonly used in human oligodendrocyte‐maturation media [125]. We observed a significant reduction at 10 mM in oligodendrocyte differentiation, cell number, and mitochondrial activity (Supporting Information File 1). While db‐cAMP activates CREB (as used here), it also signals PKA [126], potentially mediating mitochondrial activity [127]. The dose–response curve (Figure 7C) did not start at the solvent control level (100%), suggesting that effects may already occur at lower, untested concentrations. This raises the possibility of a non‐monotonic dose–response relationship. Human OPCs in our neurospheres may therefore be more sensitive to db‐cAMP exposure, potentially responding with increased differentiation at lower nM concentrations, unlike the mM range reported in rat studies [123, 124] or the µM range used in human iPSC‐derived models [128, 129]. The concentrations tested in our study were selected based on the previously published studies where functional effects of db‐cAMP had been observed, to ensure comparability across models. Additionally, CREB inhibition using KG‐501 was associated with decreased NPC proliferation (NPC1), supporting the hypothesis of Golgi stress‐induced CREB and apoptosis‐mediated microcephaly [130]. Our data might thus contribute to understanding CREB‐related diseases by identifying modulated human KNDPs. Pathogenic variants in the CREB‐binding protein were also identified to cause Rubinstein–Taybi syndrome with ID [131, 132]. Additionally, CREB signaling is crucial in the pathogenesis of schizophrenia, particularly in dopaminergic and dendritic development, interacting with neurotrophins such as BDNF, consistent with the neurodevelopmental hypothesis of this disease [114, 133]. BDNF was also used in our study as a mechanistic tool compound to activate TrkB signaling. In neural progenitor cells, BDNF and CREB are functionally and mechanistically connected: BDNF binds to TrkB, activating intracellular cascades including PLCγ releasing intracellular calcium and activating calcium/calmodulin‐dependent kinase IV (CaMKIV)‐regulated pathway and also Ras‐dependent pathway via extracellular‐regulated kinase (ERK) and ribosomal s6 kinase (RSK) signaling, all of which can converge on CREB phosphorylation [134]. Surprisingly, no significant phenotypic effects were observed following BDNF exposure in any KNDP, as discussed further in the TrkB section. The TrkB pathway is thus outside the applicability domain of the Neurosphere Assay whereas CREB belongs to it. This underscores the advantage of using compounds like db‐cAMP or KG‐501 that directly target key intracellular signaling nodes such as CREB, in contrast with BDNF which may activate CREB only via many intermediate nodes. A good selection of direct mechanistic tool compounds thus allows clearer interpretation of pathway‐specific effects within the current assay context.

Inhibition of **mitochondrial electron transport chain complex 1** by the pesticide rotenone reduced all assessed KNDPs except for neuronal and oligodendrocyte migration (NPC2bc). Rotenone is a potent DNT chemical in human‐based in vitro models [135, 136, 137, 138, 139], serving as a prototypical stressor in adverse outcome pathways linked to complex I inhibition, mitochondrial dysfunction, and dopaminergic neuron degeneration related to Parkinson's disease [140]. Expected decreases in cell number and viability in the nanomolar range (Supporting Information File 1) were observed within the concentration range affecting other endpoints (BMC 0.031–0.123 µM). Interestingly, it was suggested that rotenone‐mediated DNT effects might be also mediated via PPAR signaling [135]. Similarly to our NPC1,3,5 results, mouse neurospheres from *NDUFS2‐*deficient mice exhibited impairment of NPC proliferation and neuronal and oligodendrocyte differentiation [141]. This core unit of complex I (*NDUFS2*) is expressed in our proliferating and differentiating cultures and fetal brain samples (Figure 8). Malfunctional ETC I contributes to neurological diseases through impaired energy production and reactive oxygen species accumulation [142]. Leigh Syndrome, a severe pediatric disorder with intellectual disability (ID), exemplifies this, as patient‐derived NPCs carrying mutations in *NDUFS4* showed failed neuronal morphogenesis [143] and patient‐derived neurons with mitochondrial DNA mutation manifested mitochondrial dysfunction with an impaired calcium homeostasis important for proper neural network function [144]. Additionally, neurite outgrowth has been negatively affected in Leigh Syndrome patient‐derived iPSC models [143], corroborating our findings on the NPC4ab assays (Figure 6B,C).

The evolutionarily conserved Ser/Thr protein kinase **mTOR pathway** is represented by two protein complexes, mTORC1 and mTORC2 with mTORC1 being the rapamycin‐sensitive member [145]. The activity of mTOR is interwoven in cellular growth factors, stressors and nutrient signaling to control cell growth, metabolism and autophagy [146]. It is known to regulate the proliferation of hNPCs and par extension brain size [146]. Mutations in mTOR‐related genes (e.g., RHEB, a canonical activator of mTOR in the mTORC1 complex) were found in patients with ID associated with megalencephaly (brain overgrowth) [147]. Our data indicates that inhibition of the mTOR pathway by everolimus, a rapamycin analogue [148], decreases hNPC proliferation in the low nanomolar range in line with previous knowledge on the rapamycin‐induced reduced proliferation of human iPSC‐derived NSCs [149]. However, activation of mTOR using MHY1485 did not cause increased proliferation as expected in light of the mTORopathies like tuberous sclerosis complex, hemimegalencephaly, and focal cortical dysplasia, which are caused by mTORC1 signaling hyperactivation [150]. One reason might be that proliferating neurospheres are cultivated in presence of 20 ng/mL epidermal growth factor (EGF). mTORC1 activity is linked to the EGF‐receptor (EGFR) via mitogen‐activated protein kinases (reviewed in [146]) and hence the pathway might already be maximally activated by the 20 ng/mL of EGF in the medium in a way that proliferation cannot be triggered any further. This observation highlights a boundary of the biological applicability domain of the NPC1 assay, underscoring that certain signaling pathways may not be functionally responsive in this model. From a regulatory perspective, such defined limitations can help delineate the scope of reliable interpretation, guide chemical selection, and inform complementary assay use within IATA approaches. It would be valuable to explore whether similar limitations apply to other human NPC‐based assays within the DNT IVB, such as the hNP1 proliferation assay developed by the US EPA [17].

The **Notch signaling pathway** is crucial for regulating the neuron‐glia switch during brain development [151, 152]. First, Notch represses neuronal fate while promoting glial fate; second, it promotes the differentiation of astrocytes while inhibiting the differentiation of both neurons and oligodendrocytes [153]. When inhibiting Notch using DAPT, our cell model exhibited increased neuronal differentiation and decreased oligodendrocyte differentiation corresponding to the developmental stage of the first step of the switch. The same DAPT effects on neuronal and OPC differentiation have been observed in human NSCs [154], embryoid bodies [155], and rodents in vivo [156] and in zebrafish larvae [157] and mouse embryos [151], respectively. Surprisingly, neither NPC2b (neuronal migration) nor NPC2a (RG migration) assay was associated with Notch modulation, despite its involvement in disorders involving impaired neuronal migration such as lissencephaly (“smooth brain”), heterotopia, hemimegalencephaly [158], Adams–Oliver syndrome [159], cerebellar hypoplasia [160], and focal cortical dysplasia [161]. These disorders often manifest with ID, developmental delays, seizures, learning difficulties, and poor motor function. Contrary to our results, Keilani and Sugaya [162] observed Notch regulation of the RG extension process in human fetal NPCs. Reelin signaling requires binding to cellular very‐low‐density‐lipoprotein (*VLDLR*) or apolipoprotein E (*LRP8*) receptors, initiating a cascade leading to the Notch intracellular domain, aiding in transcriptional function. Both receptors are expressed in our cultures (Figure 8), indicating our model's capability of the Reelin–Notch crosstalk. However, Reelin might influence neural migration at later developmental stages than our fetal cell model, such as during multipolar neuronal migration and detachment of neurons from RG to undergo terminal somal translocation [163]. Of note, the neuronal migration process assessed in the NPC2b assay was the only endpoint not regulated by any tested signaling pathways. Other pathways not investigated in this project, such as cyclin‐dependent kinase 5 (Cdk5) signaling [164], might, however, regulate this endpoint. Cdk5 regulates cortical folding in vivo in gyrencephalic mammals [165] and its mutations cause lissencephaly with cerebellar hypoplasia in humans [166]. Notably, the NPC2b endpoint is rarely affected by chemical exposure, with only one out of 120 compounds (pesticide deltamethrin) affecting neuronal migration as observed in our EFSA‐supported screening study [19]. This lack of response in certain assays, despite well‐established roles of Notch in NDDs, highlights a defined boundary of the Neurosphere Assay's biological applicability domain. From a regulatory perspective, such negative results are valuable because they inform where the assay may not reliably detect specific pathway perturbations, thereby supporting its appropriate fit‐for‐purpose use within an IATA. It also emphasizes the importance of complementing the assay with additional models to fully cover the mechanistic landscape of DNT.

**PDGF signaling** is mediated through the two receptor tyrosine kinases, PDGF‐receptor (PDGFR)α and β upon binding to PDGF(A–D). PDGFRα with its ligands PDGF(A–C) plays a pivotal role during development regulating growth (reviewed in [167]). In line with this reported function, PDGFRα is higher expressed than PDGFRβ in the human NPCs used in this study. Moreover, its main ligand PDGF‐A is the strongest expressed in proliferating hNPCs, while it is down‐regulated when changing the cellular program from proliferation to differentiation (Figure 8). Inhibition of PDGFR by CP‐673451 causes a reduction of hNPC proliferation in line with the growth‐promoting function of PDGFRα in this as well as in an earlier study in vitro in rat cortical stem cells [168]. It is, however, important to note that hPDGF‐mediated activation of PDGFR did not increase hNPC proliferation in the present study, probably due to the above‐mentioned maximal activation of the EGFR pathway. While disorders such as Alzheimer's and Parkinson's disease, amyotrophic lateral sclerosis, and cerebral ischemia often involve impaired PDGF signaling (reviewed in [169]), it is often associated with the proliferation of pericytes that are crucial for the function of the blood–brain barrier [170, 171] and not hNPC proliferation. Deficient PDGF signaling in proliferating hNPCs nevertheless played a role in Parkinson's mouse model in vivo [172]. Additionally, PDGF signaling is known to regulate OPC differentiation in rodents in vivo [173], corroborating our NPC5 results following PDGF inhibition.

The **PI3K‐AKT signaling pathway** involves phosphatidylinositide 3‐kinase converting membrane phospholipid PIP2 to PIP3, recruiting and activating AKT serine/threonine kinase B to the plasma membrane, which then phosphorylates multiple downstream targets that regulate key neurodevelopmental processes like dendritic branching, synaptic development and plasticity, and neuronal survival [174]. Consistent with its role in dendritic morphogenesis and maturation [175, 176], PI3K‐AKT inhibition by LY294002 suppressed neurite outgrowth, reducing both neurite area (NPC4a) and length (NPC4b). FMRP protein, encoded by *FMR1*, negatively regulates this pathway, and PI3K‐AKT inhibition has been shown to rescue neurodevelopmental deficits in fragile X syndrome (FXS) patient‐derived organoids with *FMR1* mutation [177, 178]. This mutation also suppressed neurite outgrowth in patient‐derived iPSC neurospheres [179]. Additionally, we found that the PI3K‐AKT pathway modulation was associated with oligodendrocyte differentiation (NPC5), consistent with the dysregulated cortical oligodendrocyte lineage development observed in FXS‐associated tremor/ataxia syndrome patients [180]. Finally, impairment of this pathway is also linked to brain overgrowth‐associated disorders [181] such as megalencephaly [174], and malformations of cortical development [182], often associated with epilepsy [183]. However, no modulation of NPC proliferation (NPC1) was observed in our study, despite LY294002's inhibiting effects on NPC proliferation in other studies [184, 185, 186, 187, 188]. This inconsistency suggests that PI3K‐AKT's role in NPC proliferation may involve interactions with other pathways like ERK [189] or CREB [190] (CREB inhibition led to decreased NPC proliferation in this study) or be specific to certain NPC subpopulations or developmental stages [190]. Notably, PI3K‐AKT regulates mTORC1 activity [191]. In this study, mechanistic tool compounds were selected to act upstream (PI3K‐AKT) and downstream (mTOR) to distinguish their effects. The only shared outcome was reduced NPC5 differentiation upon PI3K‐AKT inhibition and mTOR activation which supports the robustness and physiological relevance of this endpoint as a convergent readout of pathway disruption. In contrast, other endpoints such as proliferation (NPC1) or neurite outgrowth (NPC4) may be independently regulated at distinct signaling nodes, reflecting the modular nature of intracellular signaling.

To the best of our knowledge, our study is the first to report that the **PLC pathway** is associated with the differentiation of human oligodendrocytes, a regulation also known from in vivo rodent studies [192]. Interestingly, PLC mediates also other neurodevelopmental endpoints in rodents such as neurite outgrowth and neuronal migration [193], characteristics not observed in the present study with human neurospheres. A recent publication reviewed cryptic *PLC* mutations associated with ASD, bipolar disorder, and other disorders relating this pathway to relevant NDDs [194].

While studying the applicability domain of the Neurosphere Assay, we found that **STAT3** and **TrkB pathways** were outside its scope. **STAT3**, a key transcription factor regulates astrogliogenesis in animal and human models [195, 196, 197, 198, 199] but this process is not assessed in the DNT IVB leaving an uncertainty in the covered KNDPs. STAT3 also mediates neuronal survival and regeneration after injury [200], endpoints beyond the neurodevelopmental focus of the assay. **TrkB**, activated by neurotrophins such as BDNF via PI3K, MAPK, and PLC‐ γ [201] is linked to neurodevelopmental outcomes including learning difficulties, hyperactivity, and autistic traits [202]. However, no phenotypic effects were observed following BDNF exposure in our model. Gene expression analysis showed robust *NTRK2* and *NTRK3* expression (Figure 8) suggesting receptor presence alone is insufficient for functional pathway engagement. The absence of effects may reflect immature receptor activation or incomplete downstream signaling, placing TrkB outside the assay's applicability domain. Neurotrophin signaling is highly stage‐specific. Human Brain Transcriptome data [203] indicate that cortical *BDNF* expression rises late in pregnancy and postnatally, with only a fetal peak in the striatum (Figure S2). Limited BDNF expression in our fetal Neurosphere model likely explains the lack of response. Alternative models, such as iPSC‐derived synaptogenesis assays [204, 205], capture this pathway more effectively. Given TrkB's role in dendritic arborization, synaptic plasticity [206] and neural network formation (NNF) [207], recently developed human NNF assays may complement the current DNT IVB by addressing this uncertainty [139, 208].

The **Wnt pathway** includes non‐canonical (e.g., Wnt‐calcium, planar cell polarity) and canonical pathways that stabilize and transport β‐catenin into the nucleus for gene regulation [209]. GSK3β pharmacological inhibitors like CHIR99021 boost Wnt signaling by preventing β‐catenin phosphorylation and degradation [210, 211]. CHIR99021 exposure caused increased neuronal differentiation, consistent with enhanced differentiation following GSK3β inhibition in rat neurospheres [212]. Impaired Wnt signaling and its effect on neuronal differentiation have been studied in patient‐derived models of Miller–Dieker syndrome [213], where Wnt inhibition increased neuronal differentiation and activating upstream N‐cadherin rescued the defective neurogenesis phenotype. These findings differ from our results and likely stem from variations in the developmental stages modeled in vitro, as their use of neural rosettes specifically mimics the neural tube, a structure appearing early during embryogenesis, in contrast to cells from the fetal period utilized in this study. Additionally, Wnt activation was associated with reduced RG migration (NPC2a), aligning with observed cortical tissue defects caused by defective RG organization and function in human organoids [214]. We also found decreased oligodendrocyte differentiation (NPC5) consistent with both Wnt activation (CHIR99021) and inhibition (IWP2). This underscores that both excessive activation and inhibition of Wnt signaling can impair oligodendrocyte differentiation, highlighting the need for tightly controlled Wnt/β‐catenin activity during development, accompanied by a complex interplay of timing, transcription factors (e.g., Tcf4), inhibitory signals from axons and astrocytes, and epigenetic controls [215]. Interestingly, OPCs require a vascular scaffold for migration, as observed in brain samples at gestational week 14, mediated by Wnt signaling [216]. The absence of blood vessels in our system explains why the Wnt pathway did not regulate oligodendrocyte migration in this study. Increasing model complexity by including blood vessels for example in organoid structures would enhance identification of complex modes‐of‐action like involving organ crosstalk. Due to the multitude of Wnt effects on developing brain cells (Figure 10), Wnt is still part of the DNT IVB applicability domain even if not all biological consequences of Wnt (or other) signaling pathways are covered. Autism spectrum disorder (ASD) is another NDD linked to disrupted Wnt signaling, with both gain and loss of Wnt/β‐catenin function associated with disease symptoms and neurodevelopmental deficits [217]. The gene *CTNNB1* [218], encoding β‐catenin, is highly expressed in our in vitro models (Figure 8) and is associated with NDDs like ASD, ID, schizophrenia, and microcephaly as are the KNDP radial glia migration, neuronal and oligodendrocyte differentiation and neurite outgrowth. Disturbances of different KNDP were previously linked to ASD in addition to epilepsy, ID and schizophrenia [96].

Discussion on the additional pathways, namely BMP, EGFR, NO‐cGMP, PKC, RhoA, ROCK, and SRC can be found in the Supporting Information File 1.

The phenotypic observation highlighting KNDPs consistent with signaling pathways modulation was put into the context of the current human‐oriented experimental literature (in vitro or brain samples) to create a **physiological map**, an overview of pathways and molecular interactions regulating human oligodendrocyte differentiation, serving as a hypothesis‐generating framework. We chose the proof‐of‐principle example of oligodendrocytes because there is increasing awareness for this type of macroglia to be toxicologically relevant targets and compared to neurons they have been understudied [74]. While significant progress has been made in summarizing human oligodendrocyte physiology, uncertainties remain, especially regarding differences between human and animal models commonly used in toxicity assessments. Most current insights into oligodendrocyte differentiation stem from animal studies, particularly rodents, which, despite their extensive use in studying brain development [4], exhibit potential spatiotemporal and species‐specific variations that limit extrapolation to humans [11, 219]. For example, in rodents, the FGF pathway promotes OPC generation from NPCs dependently and independently of SHH pathway regulation [220]. However, as shown in Figure 9, in humans, FGF inhibits oligodendrocyte lineage commitment in OLIG2 progenitors and favors neuronal or astrocytic lineages by suppressing exogenous SHH signaling, which causes reduced *NKX2‐2*, *OLIG2*, and *SOX10* expression, essential transcription factors regulating OPC specification. FGF also reduces endogenous SHH expression and upregulates *GLI2* and *GLI3*, both SHH pathway repressors. Conversely, at later stages, FGF promotes OPC differentiation into pre‐myelinating oligodendrocytes via PDGF pathway interaction [220], highlighting how a pathway's role can change based on the spatiotemporal scenario. This case example underscores our argument regarding the necessity for employing human‐relevant methods in toxicity assessments addressing all relevant developmental stages to better understand and characterize human physiology. Acknowledging interspecies differences is vital for developing precise chemical risk assessments.

Overall, physiological maps are powerful tools for elucidating mechanisms of adverse effects, providing insights into molecular events in oligodendrocyte differentiation, identifying potential molecular initiating events (MIEs), enriching AOP networks, and presenting a graphical form of looking at physiology similar to the early biochemical pathway maps [221]. By mapping key neurodevelopmental pathways, we can better understand how toxicants might disrupt these processes, leading to neurological impairments. Advances in single‐cell transcriptomics and proteomics can provide deeper insight into oligodendrocyte heterogeneity and signaling responses and can be visualized and analyzed on maps. These maps, as well as the relevant AOPs and AOP networks, must remain dynamic and evolve with new scientific findings to enhance accuracy and completeness, serving as essential resources for advancing human health assessments. We aim to expand the oligodendrocyte differentiation map, integrating emerging physiological and NDD‐related data [222, 223] using artificial intelligence (AI)‐assisted text mining [224] to cover all processes and cell types, and to develop predictive mechanistic computational models following the Disease Map approach [45, 225, 226]. This will further advance understanding of human brain development and the DNT IVB's relevance to human health and disease.

### 4.1 Final Remarks and Outlook

The value of NAMs in regulatory toxicology lies not only in increasing human relevance, throughput, and costs (by up to 90% when comparing DNT IVB with a guideline extended one‐generation study) but also in their capacity to generate mechanistic insights that are anchored to human biology. While in vivo DNT studies integrate effects across multiple developmental stages, they rarely allow resolution of stage/KNDP/cell type‐specific mechanisms or identification of the primary developmental window affected by a chemical. This level of specificity enables precise identification of vulnerable endpoints and pathways, facilitating upstream anchoring of AOPs and hypothesis generation. In this context, mechanistic information from NAMs may contribute to fit‐for‐purpose regulatory approaches such as IATAs, defined approaches, or chemical screening and prioritization strategies. These approaches can help focus further in‐depth testing on substances of highest concern and thereby improve the efficiency of risk assessment workflows. Such a tiered testing strategy has been recently proposed at the OECD DNT expert group and are currently being explored in case studies of the European H2020 ONTOX project. Importantly, interpretation of NAM‐derived data requires appropriate physiological context, accounting for developmental stage and cell‐type specificity, particularly in complex systems such as the human brain.

In this context, the present work characterizes the biological applicability domain of the Neurosphere Assay, a central component of the DNT IVB that models seven KNDPs of human brain development. The Neurosphere Assay has been previously used to assess, for example, a set of 120 reference compounds, including 46 performance compounds (DNT‐positive in humans, in vivo, and human DNT‐negative substances) and environmental chemicals, mostly pesticides. The assay demonstrated high specificity and sensitivity in identifying compounds with reported DNT potential in humans and in vivo guideline studies [19]. These results support the assay's relevance for regulatory applications and complement the mechanistic insights generated in the present study. Taken together, the results reveal several higher‐level patterns that help define the biological applicability domain of the Neurosphere Assay. Most investigated signaling pathways produced associated measurable phenotypic responses in at least one KNDP, indicating broad mechanistic coverage of neurodevelopmental processes within this model. Oligodendrocyte differentiation emerged as the most pathway‐sensitive endpoint, being associated with 13 signaling pathways. This confirms the complex regulation of oligodendrocyte development [227, 228] and may explain the high sensitivity of the NPC5 assay in earlier screening studies [19, 97, 98]. Radial glia migration (NPC2a assay), followed by NPC proliferation (NPC1 assay) also responded to multiple pathway perturbations (11 and 9, respectively), whereas neuronal migration remained largely unaffected by the tested signaling modulators. Mitochondrial ETC I pathway modulation was strongly associated with multiple endpoints, consistent with Leigh Syndrome pathophysiology [141, 142, 143, 144]. Namely, we observed that the Neurosphere Assay can detect chemicals interfering with most tested pathways except for STAT3 and TrkB, suggesting that these pathways fall outside of its applicability domain. Future work should explore additional signaling pathways (e.g., MAPK [229], CDK5 [230], JNK [231], ROR‐α [232, 233], and glycosylation [234]).

While this study functionally links pathway modulation to changes in KNDPs using the Neurosphere Assay, it is important to note that we did not independently verify the activation status or specificity of the targeted pathways through molecular readouts such as transcriptomic profiling or phosphoprotein analysis. Pathway‐KNDP associations presented here are based on the phenotypic effects of well‐studied mechanistic tool compounds for selected signaling pathways and should be regarded as hypothesis‐generating. Possible off‐target effects (e.g., for db‐cAMP, rotenone, reelin, U73122, colivelin, limonin, and CHIR, see Table S3) and uncertainties in actual bioavailable concentrations, due to factors such as adsorption to plastics, protein/lipid binding, volatility, or degradation, must be considered [235]. Although analytical verification of bioavailable concentrations was not feasible here, ongoing ONTOX efforts aim to systematically characterize Neurosphere assay parameters relevant for in silico modeling of compound distribution and bioavailability, including the protein and lipid content of the cellular system, the composition of the culture medium, and the material properties of the well plates, to enable future in silico modeling of chemical distribution and bioavailability [43, 235]. These limitations should be kept in mind when interpreting concentration–response relationships presented in the current study.

In conclusion, we strengthened the mechanistic interpretability of the Neurosphere Assay by further defining its biological applicability domain and linking KNDPs to human physiology and disease. We show that oligodendrocyte differentiation is among the most tightly regulated KNDPs, while STAT3 and TrkB pathways are outside the assay's scope. Interpretation should remain context‐specific, as pathway effects are inherently stage‐ and cell type‐dependent, and in vitro results cannot be directly extrapolated to in vivo scenarios without careful consideration. Within these boundaries, the physiological map and functional profiling presented here serve as valuable tools to refine and mechanistically anchor DNT NAMs, improve confidence in their human relevance, and support their integration into regulatory hazard assessment frameworks.

## Author Contributions

E.K.—Validation, Formal Analysis, Investigation, Data Curation, Writing – Original Draft, Writing – Review and Editing, Visualization, Project Administration, Supervision. K.B.—Data Curation, Investigation, Formal Analysis, Supervision, Writing – Original Draft, Writing – Review and Editing. G.R.—Investigation. M.S.—Investigation. L.L.—Investigation, Data Curation, Visualization, Writing – Original Draft, Writing – Review and Editing. A.D.—Methodology, Software, Writing – Review and Editing. J.K.—Investigation. N.G.—Investigation. D.P.—Investigation. L.S.—Investigation. F.B.—Investigation. B.S.–Investigation, Data Curation, Writing – Review and Editing. L.G.—Investigation, Data Curation. K.K.—Writing – Review and Editing, Supervision. E.F.—Conceptualization, Writing – Review and Editing, Supervision, Project administration, Funding acquisition.

## Funding

This work was performed in the context of the ONTOX project (https://ontox‐project.eu/) and the European Partnership for the Assessment of Risks from Chemicals (PARC) that has received funding from the European Union's Horizon 2020 Research and Innovation programme under grant agreement No. 963845 and European Union’s Horizon Europe research and innovation programme under Grant Agreement No. 101057014, respectively. ONTOX is part of the ASPIS project cluster (https://aspiscluster.eu/). We also gratefully acknowledge that portions of this research were supported by the Division of Translational Toxicology, National Institute of Environmental Health Sciences, National Institutes of Health, Department of Health and Human Services ZIA ES103387‐02.

## Conflicts of Interest

E.K., G.R., M.S., L.L., N.G., D.P., L.S., B.S., F.B., J.K., and L.G. declare no conflict of interest. A.D., E.F., K.B., and K.K. are shareholders of the company DNTOX which provides DNT IVB assay services, and all declare no potential conflicts of interest with respect to the research in this article.

## Supporting information




**Supporting File 1**: advs75183‐sup‐0001‐SuppMat.docx.

**Supporting File 2**: advs75183‐sup‐0002‐DataFile.xlsx.

## Data Availability

The data that support the findings of this study are openly available in BioStudies at https://www.ebi.ac.uk/biostudies/ONTOX/studies, reference number starting at S‐ONTX^37^ until S‐ONTX57. The physiological map and its related files are available at our GitHub organization (https://github.com/ontox‐maps/paper_data_OligDev/), and at the ONTOX MINERVA platform (https://ontox.elixir‐luxembourg.org/minerva/), under the License Creative Commons Attribution 4.0 International (CC BY 4.0) License (https://creativecommons.org/licenses/by/4.0/).
