# Supplementary material for: Functional Mapping of Neurodevelopmental Disease Pathways to Key Neurodevelopmental Processes Represented in the Developmental Neurotoxicity In Vitro Testing Battery
Source: Adv Sci (Weinh). 2026 Apr 14:e19889. Online ahead of print. doi: 10.1002/advs.202519889 (PMC13334671; doi:10.1002/advs.202519889)
Supplement: Supplementary file 1 — Supporting File 1: advs75183‐sup‐0001‐SuppMat.docx. [file ADVS-9999-e19889-s002.docx]

Functional Mapping of Neurodevelopmental Disease Pathways to Key Neurodevelopmental Processes Represented in the Developmental Neurotoxicity In Vitro Testing Battery

Eliska Kuchovska^1^*, Kristina Bartmann^1,2^*, Georgea Raad^1^, Mats Schade^1^, Luiz Ladeira^3^, Arif Dönmez^1,2^, Jördis Klose^1,2^, Nicolai Görts^1^, Denis Polozij^1^, Lynn-Christin Saborowski^1^, Farina Bendt^1,2^, Bernard Staumont^3^, Liesbet Geris^3,4,5^ , Katharina Koch^1,2#^, Ellen Fritsche^1,2,6^

^1^IUF - Leibniz Research Institute for Environmental Medicine, Düsseldorf, Germany; ^2^DNTOX GmbH, Düsseldorf, Germany; ^3^GIGA Molecular and Computational Biology, University of Liège, Belgium; ^4^Skeletal Biology and Engineering Research Center, KU Leuven, Belgium; ^5^Biomechanics Section, KU Leuven, Belgium; ^6^SCAHT - Swiss Centre for Applied Human Toxicology & Department of Pharmaceutical Sciences, University of Basel, Switzerland;

*Authors contributed equally ^#^corresponding author

**Supplemental word file**

**Table of contents**

[1. Additional discussion 2](#_Toc204872783)

[2. Figure S1 4](#_Toc204872784)

[3. Figure S2 5](#_Toc204872785)

[4. Table S1: List of chemicals used to modulate the investigated pathways in our previously published works. 6](#_Toc204872786)

[5. Table S2: Summary of obtained BMCs, BMCLs, and BMCUs of the affected specific endpoints. 7](#_Toc204872787)

[6. Table S3: Mechanistic tool compounds targeting the signaling pathways: reasons for selection and off-target effects, and physiologically relevant systemic levels of endogenous chemicals 8](#_Toc204872788)

[7. References 9](#_Toc204872789)

[8. No-effect results of specific endpoints 13](#_Toc204872790)

[a) Figure S3 - NPC1 14](#_Toc204872791)

[b) NPC2a 16](#_Toc204872792)

[c) NPC2b 18](#_Toc204872793)

[d) NPC2c 21](#_Toc204872794)

[e) NPC3 24](#_Toc204872795)

[f) NPC4 26](#_Toc204872796)

[g) NPC5 30](#_Toc204872797)

[9. Non-specific endpoints 32](#_Toc204872798)

[a) VIABILITY 72 h 32](#_Toc204872799)

[b) VIABILITY 120 h 35](#_Toc204872800)

[c) CELL NUMBER 120 h 38](#_Toc204872801)

[10. Endpoint-specific positive controls 41](#_Toc204872802)

# Additional discussion

This section includes an additional discussion of pathways investigated in our previous work (BMP, EGFR, NO-cGMP, PKC, RhoA, ROCK, and SRC) to complement the biological applicability domain of the Neurosphere Assay.

The **bone morphogenetic protein (BMP)** signaling pathway regulated oligodendrocyte differentiation (**Figure 10**). BMPs are morphogens from the transforming growth factor beta (TGFβ) ligand family binding to BMP receptors that mediate further downstream signaling.^[1]^ The dysregulation of the BMP pathway was observed in human postmortem samples of multiple sclerosis lesions; BMPs regulate inflammation but are also upregulated in demyelinating conditions which are directly linked to the impairment of oligodendrocyte development.^[2]^ Moreover, BMP pathway inhibition modulated rescued neurite outgrowth impairment in Fragile X Syndrome human embryonic stem cells that were induced to mimic early neurodevelopment.^[3]^ Concordantly with our NPC5 results, BMP pathway is known to inhibit oligodendrocyte differentiation in rodents^[4–6]^ but also to modulate the radial glia development^[7]^ as observed in our NPC2a assay.^[8]^ Moreover, BMP7 protein is used as a positive control inhibiting oligodendrocyte differentiation in the NPC5 assay. Interestingly, we observed species differences in BMP regulation of neuronal differentiation.^[8]^ This advocates for using human NAMs using *in vitro* model from a developmental stage of interest for human risk assessment.

The **epidermal growth factor receptor (EGFR)** belongs to the receptor tyrosine kinase family and can be activated by several ligands including the epidermal growth factor (EGF) **.** After its activation, EGFR further triggers downstream cascades regulating among others the neural stem cell pool and proliferation of neural progenitors, cell survival, astrocyte differentiation and maturation, oligodendrogenesis, and neurite outgrowth.^[9]^ We previously showed and discussed that the EGFR pathway regulated key neurodevelopmental process (KNDP) of human neural progenitor cells (NPC) proliferation (NPC1), radial glia migration (NPC2a), and neuronal and oligodendrocyte differentiation (NPC3,5).^[8,10]^ We used the activator EGF and the inhibitor PD153035 to increase and suppress NPC proliferation, respectively.^[10]^ EGFR-mediated regulation of this KNDP has been shown previously in *in vitro* murine neural stem cells and progenitors,^[11–13]^ and *in vivo* in rodent brain and spinal cord.^[14,15]^ Similarly to our NPC2a results, migration of neural progenitors has been enhanced by EGF exposure in *in vitro* mouse cultures^[16]^ and in rodent explant cultures.^[17]^ Furthermore, the EGFR pathway regulated neuronal differentiation and oligodendrocyte development in human fetal neurospheres^[18]^ and in mice *in vitro*,^[19]^ respectively, corroborating our NPC3 and NPC5 findings. These findings show that hNPCs recapitulate physiological EGFR functions *in vitro*. Importantly, our findings regarding the regulation of hNPC proliferation (NPC1) are consistent with the well-described involvement of the EGFR pathway in microcephaly manifesting by decreased proliferation *in vivo*.^[20,21]^

**NO-cGMP pathway** was studied in our previous publication.^[22]^ Nitric oxide (NO) activates the synthesis of cyclic guanosine monophosphate (cGMP) and is subsequently hydrolyzed by phosphodiesterases (PDEs). Genetic variations in PDEs, including single-nucleotide polymorphisms, are associated with neurodevelopmental disorders (NDDs) such as autism spectrum disorder (ASD), Rett syndrome, and intellectual disability.^[23]^ The link between NO-cGMP and ASD was recently further strengthened by a study using iPSC-derived cortical neurons from patients with *SHANK3* mutations, clinical plasma samples, and *in vivo* models with ASD phenotypes reversed using a neuronal NO synthase inhibitor.^[24]^ NO performs a neurotransmitter-like role and is involved in neurogenesis, neuronal differentiation, neuroprotection, synaptic plasticity, and learning and memory formation.^[25,26]^ In our previous publication, activation and inhibition of the **NO-cGMP pathway**, led to increased and decreased radial glia migration.^[22]^ This regulation has been previously described *in vivo* in *Xenopus laevis* tadpoles mediated by neuronal NMDA receptor activation-dependent NO release^[27]^ and further reviewed in the work of Contestabile and team.^[28]^

We further investigated the **protein kinase C (PKC) pathway** whose activity is high in brain tissue and is expressed in both neuronal and glial cells.^[29]^ PKC family is composed of several intracellular kinases that can be triggered among others by Ca^2+^ and diacylglycerol, subsequently affecting different signal transduction pathways like the ERK-MAPK cascade, making PKC involved in myriad cellular functions^[30]^ such as neurite development and cell migration as shown further. PKC activation at the synapses phosphorylates MARCKS disrupting dendritic spine morphology^[31]^ while PKC-ε inhibition by bisindolylmaleimide I reduces neurite outgrowth.^[32,33]^ Bisindolylmaleimide I, a C3 domain-binding and ATP-competitive PKC inhibitor targets the conventional PKC isoforms.^[34,35]^ We show that this inhibitor reduced both neurite length (NPC4a) and area (NPC4b),^[10,36]^ corroborating earlier findings in another human-based *in vitro* model.^[37]^ We additionally observed reducing effects on radial glia migration (NPC2a) after PKC inhibition which corresponds with previously reported PKC role in apical-basal polarity.^[38]^ In the mentioned study, the authors show that the knockout of one of the isoforms of aPKC, aPKC-λ, in neuroepithelial cells and radial glial cells resulted in the loss of apical processes that cause disordered layering of the cortex.^[38]^ **Table 1** further highlights the involvement of PKC in NDDs. For example, mutations in *PKC* genes are linked to disorders such as spinocerebellar ataxia, a progressive disorder with degeneration and dysfunction of the cerebellum, in which abnormal mGluR1-PKCγ signaling impairs dendritic development of Purkinje cells (reviewed in ^[39]^).

**RhoA**, a small GTPase protein in the Rho family of GTPases, is crucial for actomyosin structure regulation, junction assembly, stability, and function.^[40,41]^ The RhoA pathway regulated almost all KNDPs measured in the Neurosphere Assay except for radial glia and neuronal migration. Most noticeably, the RhoA pathway was the only one of the pathways studied here that affected oligodendrocyte migration (NPC2c assay, **Supplemental Figure 1**). Additionally, our findings of decreased oligodendrocyte migration mediated via the RhoA pathway activation align with *in vivo* rodent studies^[42]^ and represent, to the best of our knowledge, the first such findings in a human model. In humans, RhoA misregulation is linked to ID.^[43]^ The RhoA pathway can also be activated by genes involved in NPC proliferation such as *ARHGEF39* which has been linked to developmental language disorder.^[44]^ Pathophysiological mechanisms involving another group of Rho GTPases, the Rac subfamily, cause neuro-RACopathies affecting intellectual development.^[45]^

**Rho-associated coiled-coil containing kinases (ROCK)** have a critical role in regulating the cytoskeleton dynamics responsible for cell adhesion, proliferation, motility, and contraction.^[46]^ Correspondingly, we found previously that inhibition of ROCK caused decreased radial glia migration.^[22]^ The KNDP assessed in NPC2a has been the only KNDP assessed in relation to this pathway. It was, however, additionally reported that ROCK induced human oligodendrocyte precursor cell differentiation.^[47]^ Future work should therefore focus on extending the biological applicability domain of the Neurosphere Assay in this (NPC5) direction. The ROCK pathway has been linked to several NDDs (**Table 1**). For example, it was found to be hyperactivated in patients with intellectual and learning disabilities as a consequence of the oligophrenin gene (*OPHN1*) loss-of-function mutation.^[48]^ This mutation was linked to neurodevelopmental phenotypes of abnormal dendritic morphology, neurogenesis, and maturation defects in a differentiating iPSC patient-derived cell model with subsequently rescued effects using a ROCK inhibitor^[49]^ as well as impaired astrocyte migration.^[50]^

Lastly, **SRC** is one of the members of the Src family kinases (SFK) which are a group of non-receptor tyrosine kinases playing a crucial role in many cellular responses such as the regulation of synaptic transmission^[51]^ and microglia homeostasis and activation.^[52,53]^ We observed decreased radial glia migration (NPC2a) following inhibition of SRC using a selective inhibitor of Src family kinases PP2.^[54]^ SRC inhibition rescued the Fragile X syndrome phenotype in a mouse model^[55]^ which should be further confirmed using patient-derived iPSC model for human relevancy (this pathway-NDD pair is therefore not shown in **Table 1** which contains only human data-based publications). SRC impairment may also be linked to schizophrenia via NMDAR hypofunction^[56]^ and to Noonan syndrome manifesting perturbed cortical layer identity and neuronal connectivity.^[57]^

# Figure S1


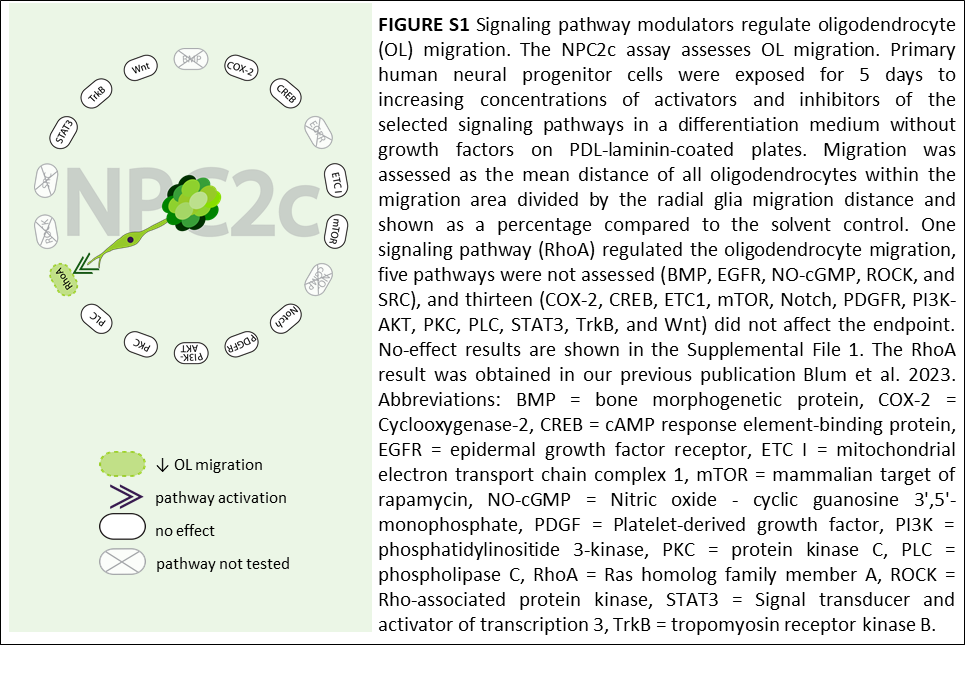


# Figure S2


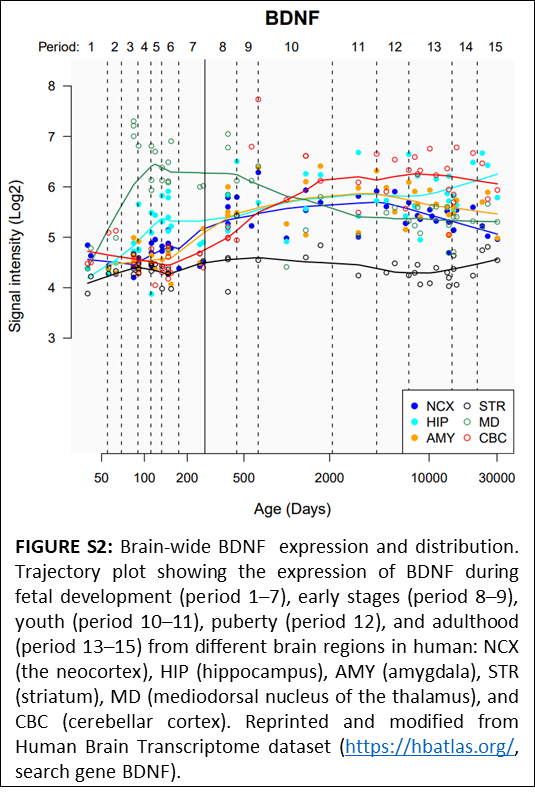


# Table S1: List of chemicals used to modulate the investigated pathways in our previously published works.

| **Reagent** | **Pathway** | **Source** | **Catalog number** | **Solvent** | **Purity** | **Tested range of concentrations** | **Source publication** |
| --- | --- | --- | --- | --- | --- | --- | --- |
| BMP2 | BMP activator | R&D Systems | 355-BM | B27 medium | / | 0.01-100 ng/mL | ^[8]^ |
| EGF | EGFR activator | Life Technologies | PHG0313 | DPBS +/+ | / | 20 ng/ml | ^[10]^ |
| PD153035 | EGFR inhibitor | Sigma-Aldrich | SML0564 | DMSO | / | 0.5-10 µM (NPC2-5) | ^[10]^ |
| Rotenone | ETC complex I inhibitor | Rotenone Santa Cruz | / | DMSO | / | Rotenone 0.01 - 2.22 µM | ^[10,58]^ |
| NOC-18 | NO-cGMP activator | Calbiochem | / | 10 mM NaOH solution | / | 1-100 µM | ^[22]^ |
| 7-nitroindazole (7NI) / ODQ (1H-[1,2,4]-oxadiazolo[4,3-a]quinoxalin-1- one) | NO-cGMP inhibitor | Sigma | / | DMSO | / | 7NI 100-500 µM; ODQ 10-50 µM | ^[22]^ |
| Bis-I | PKC inhibitor | Merck | 203290 | DMSO | / | 0.03-20 µM | ^[10,58]^ |
| Narciclasine | RhoA activator | Cayman Chemicals | 20361 | DMSO | / | 0.0001 - 0.1 µM | ^[10,58]^ |
| Y-27632 | ROCK inhibitor | Sigma | / | Differentiation medium | / | 50 µM | ^[22]^ |
| PP2 | SRC inhibitor | Calbiochem | / | / | / | 5-10 µM | ^[54]^ |

# Table S2: Summary of obtained BMCs, BMCLs, and BMCUs of the affected specific endpoints.

**Abbreviations:** BC.4 = Brain-Cousens (hormesis) with lower limit at 0, BC.5 = Brain-Cousens (hormesis), BMC = benchmark concentration, BMCL = Lower BMC confidence interval, BMCU = Upper BMC confidence interval, BMR = benchmark response, Dec. = decrease., EXD.2 = Exponential decay with lower limit at 0, EXD.3 = Shifted exponential decay, Inc. = increase, LL2.3 = Log-logistic (log(ED50) as parameter) with lower limit at 0, lm = linear; L3 = Logistic (ED50 as parameter) with lower limit fixed at 0, W1.3 = Weibull (type 1) with lower limit at 0, W2.3 = Weibull (type 2) with lower limit at 0.

| **Compound** | **Pathway** | **Activator/ Inhibitor** | **Assay** | **BMC** | **BMCL** | **BMCU** | **BMR** | **Effect** | **Model abbreviation** |
| --- | --- | --- | --- | --- | --- | --- | --- | --- | --- |
| PGE2 | COX-2 | Activator | NPC1 | **0.138** | **0.608** | **5.262** | -15 | Inc. | BC.4 |
| PGE2 | COX-2 | Activator | NPC3 | **19.092** | **13.785** | **NA** | 35 | Dec. | lm |
| celecoxib | COX-2 | Inhibitor | NPC1 | **0.299** | **NA** | **1.893** | 15 | Dec. | W2.3 |
| celecoxib | COX-2 | Inhibitor | NPC5 | **2.883** | **1.793** | **2.883** | 35 | Dec. | EXD.2 |
| celecoxib | COX-2 | Inhibitor | Cell n. | **1.995** | **1.258** | **3.150** | 25 | Dec. | EXD.3 |
| db-cAMP | CREB | Activator | NPC2a | **52.997** | **26.102** | **94.007** | 10 | Dec. | EXD.3 |
| db-cAMP | CREB | Activator | NPC3 | **44.628** | **128.727** | **5468.671** | -35 | Inc. | BC.5 |
| db-cAMP | CREB | Activator | NPC5 | **506.060** | **115.510** | **3451.901** | 35 | Dec. | LL2.3 |
| db-cAMP | CREB | Activator | Cell n. | **144.226** | **99.307** | **220.533** | 25 | Dec. | EXD.3 |
| KG-501 | CREB | Inhibitor | NPC1 | **5.036** | **1.993** | **7.851** | 15 | Dec. | lm |
| rotenone | ETC c. I | Inhibitor | NPC1 | **0.031** | **0.016** | **0.057** | 15 | Dec. | EXD.3 |
| rotenone | ETC c.I | Inhibitor | NPC2a | **0.123** | **0.047** | **0.257** | 10 | Dec. | LL2.3 |
| rotenone | ETC c.I | Inhibitor | NPC3 | **0.037** | **0.023** | **0.060** | 35 | Dec. | LL2.3 |
| rotenone | ETC c.I | Inhibitor | NPC4a | **0.046** | **0.019** | **0.083** | 20 | Dec. | EXD.3 |
| rotenone | ETC c.I | Inhibitor | NPC4b | **0.068** | **0.038** | **0.127** | 20 | Dec. | EXD.3 |
| rotenone | ETC c.I | Inhibitor | NPC5 | **0.078** | **0.038** | **0.161** | 35 | Dec. | LL2.3 |
| MHY1485 | mTOR | Activator | NPC1 | **4.317** | **2.475** | **7.317** | 15 | Dec. | EXD.2 |
| MHY1485 | mTOR | Activator | NPC2a | **18.097** | **12.730** | **NA** | 10 | Dec. | lm |
| MHY1485 | mTOR | Activator | NPC5 | **1.612** | **0.539** | **3.900** | 35 | Dec. | LL2.3 |
| everolimus | mTOR | Inhibitor | NPC1 | **0.005** | **0.003** | **0.008** | 15 | Dec. | EXD.3 |
| reelin | Notch | Activator | NPC4b | **0.021** | **0.013** | **NA** | 20 | Dec. | EXD.2 |
| DAPT | Notch | Inhibitor | NPC1 | **0.551** | **0.073** | **4.924** | 15 | Dec. | LL2.3 |
| DAPT | Notch | Inhibitor | NPC3 | **0.034** | **NA** | **0.208** | -35 | Inc. | LL2.3 |
| DAPT | Notch | Inhibitor | NPC5 | **0.057** | **NA** | **0.378** | 35 | Dec. | LL2.3 |
| CP-673451 | PDGFR | Inhibitor | NPC1 | **2.568** | **1.847** | **3.460** | 15 | Dec. | BC.5 |
| CP-673451 | PDGFR | Inhibitor | NPC2a | **0.296** | **NA** | **0.631** | 10 | Dec. | EXD.2 |
| CP-673451 | PDGFR | Inhibitor | NPC5 | **0.209** | **0.107** | **0.361** | 35 | Dec. | W1.3 |
| CP-673451 | PDGFR | Inhibitor | Cell n. | **0.306** | **0.142** | **0.516** | 25 | Dec. | EXD.2 |
| LY294002 | PI3K-AKT | Inhibitor | NPC4a | **4.020** | **3.012** | **5.715** | 20 | Dec. | EXD.2 |
| LY294002 | PI3K-AKT | Inhibitor | NPC4b | **5.465** | **4.205** | **7.590** | 20 | Dec. | EXD.2 |
| LY294002 | PI3K-AKT | Inhibitor | NPC5 | **2.958** | **1.572** | **6.172** | 35 | Dec. | EXD.2 |
| m-3M3FBS | PLC | Activator | NPC2a | **5.430** | **4.608** | **6.494** | 10 | Dec. | lm |
| m-3M3FBS | PLC | Activator | NPC5 | **0.730** | **0.434** | **1.164** | 35 | Dec. | EXD.2 |
| CHIR99021 | Wnt | Activator | NPC2a | **0.108** | **NA** | **0.317** | 10 | Dec. | EXD.3 |
| CHIR99021 | Wnt | Activator | NPC3 | **0.068** | **NA** | **0.393** | -35 | Inc. | L.3 |
| CHIR99021 | Wnt | Activator | NPC4a | **0.347** | **0.100** | **1.120** | 20 | Dec. | W1.3 |
| CHIR99021 | Wnt | Activator | NPC5 | **0.143** | **0.112** | **0.187** | 35 | Dec. | EXD.2 |
| CHIR99021 | Wnt | Activator | Cell n. | **0.270** | **0.082** | **0.778** | 25 | Dec. | EXD.3 |

# Table S3: Mechanistic tool compounds targeting the signaling pathways: reasons for selection and off-target effects, and physiologically relevant systemic levels of endogenous chemicals in the developing human brain.

It is important to note that while the physiological and clinical relevance of tested concentrations is an important consideration in toxicological studies, the goal of the present study was not to establish human exposure-relevant thresholds, but rather to explore the mechanistic responsiveness of the DNT IVB endpoints to modulation of neurodevelopmental disease-related pathways. Therefore, concentrations were selected based on their ability to modulate the respective pathway without inducing cytotoxicity and not to reflect the physiological levels (in the few cases were applicable) of the tested chemicals.

| **Reagent** | **Pathway** | **Act./Inh.** | **Physiological levels of endogenous chemicals** | **Reason for selection** | **Off-target effects** |
| --- | --- | --- | --- | --- | --- |
| **PGE2** | COX-2 | Act. | < 10 nM in CNS^[59]^ | Surrogate endogenous metabolite mimicking COX-2 activation. PGE2 is produced by COX-2 enzyme.^[60]^ | **No off-target effects found.** PGE2 is the compound that is produced when COX-2 is activated; thus, no off-target effects are relevant. |
| **Celecoxib** | COX-2 | Inh. | / | Known specific COX-2 inhibitor synthesized for this purpose.^[61]^ | **No off-target effects found.** |
| **db-cAMP** | CREB | Act. | nM range baseline to µM when stimulated in CNS^[[1]](#footnote-1)[62]^ | Db-cAMP is a synthetic derivative of cAMP, which is unstable for experimental use. It binds to PKA, which phosphorylates CREB.^[63]^ | cAMP acts through one intermediate node – protein kinase A (PKA), which can phosphorylate also other substrates than CREB. Independent of PKA, cAMP can bind to Epac, which activates ERK1/2 signaling, which subsequently phosphorylates CREB.^[63]^ PKA has been also found to phosphorylate Rap1 leading to extracellular signal-regulated kinase (ERK) cascade.^[64]^ |
| **KG-501** | CREB | Inh. | / | A known specific inhibitor of CREB synthesized for this purpose.^[65]^ | **No off-target effects found.** |
| **Rotenone** | ETC I | Inh. | / | Potent inhibitor of the mitochondrial complex 1.^[66]^ | Rotenone is a potent DNT chemical. Its primarily mode of action (MoA) is the inhibition of complex I of the mitochondrial chain (see AOP n.3 on the AOP wiki)^[67]^ but it may also depolymerize microtubules via RhoA/ROCK pathway^[68]^ and activate ROS-generating enzymes via different MoAs than via ECT I.^[69]^ |
| **MHY1485** | mTOR | Act. | / | Potent synthetic activator of mTOR pathway.^[70]^ | **No off-target effects found.** Inhibitory effect on autophagy as a downstream effect of the mTOR activation.^[70]^ |
| **Everolimus** | mTOR | Inh. | / | Rapamycin analog. It allosterically inhibits mTOR, preventing the assembly of the mTOR complex 1 (mTORC1).^[71]^ | **No off-target effects found.** Other signaling pathways may be influenced by mTOR inhibition such as NF-kB/IL-6 signaling but these are downstream of everolimus mTOR inhibition and have not been reported systematically.^[72]^ |
| **Reelin** | Notch | Act. | unknown | Reelin binds to cellular very-low-density-lipoprotein (VLDLR) or apolipoprotein E (LRP8) receptors, initiating a cascade including Disabled-1 (Dab1) tyrosine phosphorylation leading to the Notch intracellular domain (NICD). This interaction is crucial for neurodevelopment.^[73]^ | The Dab1 phosphorylation leads at the same time to activation of other downstream signaling pathways than Notch such as Crk/CrkL-Rap1 cascade and inhibition of GSK3β (reviewed in Fig.1 Bock & May 2016).^[74]^ The involved downstream pathways also depend on the maturation stage of the system.^[75]^ |
| **DAPT** | Notch | Inh. | / | DAPT is an inhibitor of the γ-secretase complex. Notch is a key target of γ-secretase, therefore, DAPT indirectly inhibits the Notch pathway and is routinely used for this purpose in literature.^[76–78]^ | DAPT can impact cleavage of other γ-secretase targets, such as amyloid precursor protein (APP), involved in Alzheimer's disease (not relevant for neurodevelopment).^[79]^ |
| **hPDGF** | PDGFR | Act. | unknown | Platelet-derived growth factor (PDGF) binds to its cognate PDGF receptor activating PDGF signaling.^[80]^ | **No off-target effects found.** PDGF receptor activates other downstream signaling pathways like GSK3β, PLC, ERK, but these are downstream pathways mediated through the PDGFR. PDGF is thus a direct activator of PDGFR without any intermediate nodes (reviewed in fig.2 Li et al. 2022).^[81]^ |
| **CP-673451** | PDGFR | Inh. | / | CP-673451 is a potent inhibitor of both PDGFR-β and PDGFR-α kinases and is >200-fold selective versus a variety of other kinases.^[82]^ | **No off-target effects found.** |
| **SC79** | PI3K-Akt | Act. | / | SC79 acts directly and specifically on Akt, binding its PH domain.^[83,84]^ | **No off-target effects found.** |
| **LY294002** | PI3K-Akt | Inh. | / | LY294002 acts as a reversible, ATP-competitive inhibitor binding the catalytic site of PI3K subsequently inhibiting PI3K-Akt signaling pathway.^[85]^ | One study indicated that LY294002 may inhibit mTOR of the same signaling cascade PI3K-Akt-mTOR^[86]^ and casein kinase 2 (CK2), another protein kinase.^[87]^ |
| **m-3M3FBS** | PLC | Act. | / | m-3M3FBS directly activates PLC and subsequently enhances the superoxide-generating activity and intracellular calcium release.^[88]^ | **No off-target effects found.** However, one study suggested that the calcium release is independent of the PLC activity via another, unknown, mode of action.^[89]^ |
| **U73122** | PLC | Inh. | / | U73122 is widely used as an inhibitor of the PLC pathway. ^[90,91]^ It inhibits the receptor-coupled generation of inositol 1,4,5-trisphosphate and intracellular calcium.^[92]^ | Several studies contradicted the specificity of U73122 and attributed its effects on intracellular calcium and PLC to off-target effects.^[93,94]^ |
| **Colivelin** | STAT3 | Act. | / | Colivelin is a potent synthetic peptide, a derivative of humanin, indirect activator of STAT3. It is composed of a part of humanin fused with activity-dependent neurotrophic factor (ADNF).^[95]^ | Colivelin activates STAT3 via the humanin component but it was also reported to activate Ca2+/calmodulin-dependent protein kinase IV (CaMKIV) pathway mediated via the ADNF component.^[96]^ |
| **Limonin** | STAT3 | Inh. | / | Limonin indirectly inhibits STAT3 via inhibition of receptor tyrosine kinase signaling upstream of STAT3 phosphorylation.^[97–99]^ | Limonin has been found to have off-target effects in skin (mTOR inhibition),^[100]^ heart tissue (rno-miR-10a-5p-IGLON5/LMX1A axis via miRNA-mRNA network),^[101]^ and liver (NLRP3/Gasdermin D Signaling Pathway).^[102]^ |
| **BDNF** | TrkB | Act. | Unknown in CNS, ± 35 ng/mL in amniotic fluid (2^nd^ trimester) | BDNF directly activates the tropomyosin receptor kinase B (TrkB) receptor.^[103]^ | **No off-target effects found.** TrkB activation by BDNF happens directly without any intermediate nodes and subsequently leads to activation of other downstream signaling pathways such as PLCγ, PI3K, and MAPK/ERK.^[103]^ Additionally, pro-BDNF (immature BDNF) can bind the low-affinity p75 neurotrophin receptor (p75NTR), irrelevant to this study. |
| **ANA-12** | TrkB | Inh. | / | ANA-12 is a selective, small-molecule non-competitive antagonist of the TrkB receptor and directly inhibits TrkB activation by BDNF without affecting TrkA or TrkC receptors.^[104]^ | **No off-target effects found.** |
| **CHIR** | WNT | Act. | / | CHIR (CHIR-99021) is a “gold standard”, potent inhibitor with high selectivity against GSK-3α and GSK3β. After this inhibition, β-catenin phosphorylation is prevented, leading to stabilization and accumulation of β-catenin in the cytoplasm. The β-catenin then translocates to the nucleus to initiate transcription of Wnt target genes.^[105]^ | CHIR is specific to GSK in low nanomolar concentrations. Higher micromolar concentrations might have off-target effects by activation of other kinases.^[105]^ |
| **IWP2** | WNT | Inh. | / | IWP-2 is a well-known small molecule inhibitor that specifically targets the enzyme Porcupine (Porcn), a membrane-bound O-acyltransferase essential for the palmitoylation and subsequent activation of Wnt proteins.^[106]^ | **No off-target effects found.** IWPs have been shown to block several Wnt-dependent biochemical changes including phosphorylation of the Lrp6 receptor and Dvl2 and β-catenin accumulation.^[106]^ Additionally, they inhibit the activity of CK1δ which phosphorylate axin and dishevelled (DVL) thereby causing a conformational change in the β-catenin destruction complex, which prevents β-catenin from being degraded.^[107]^ |

**Abbreviations:** Act. = activator, BDNF = brain-derived neurotrophic factor, BMP = bone morphogenetic protein, db-cAMP = dibutyryl cyclic adenosine monophosphate, CNS = central nervous system, COX-2 = Cyclooxygenase-2, CREB = cAMP response element-binding protein, EGFR = epidermal growth factor receptor, ETC I = mitochondrial electron transport chain complex 1, Inh. = inhibitor, mTOR = mammalian target of rapamycin, NO-cGMP = Nitric oxide - cyclic guanosine 3',5'-monophosphate, PDGF = Platelet-derived growth factor, PGE2 = prostaglandin E2, PI3K = phosphatidylinositide 3-kinase, PKC = protein kinase C, PLC = phospholipase C, RhoA = Ras homolog family member A, ROCK = Rho-associated protein kinase, R, STAT3 = Signal transducer and activator of transcription 3, TrkB = tropomyosin receptor kinase B.

# References

1. Bond AM, Bhalala OG, Kessler JA. The dynamic role of bone morphogenetic proteins in neural stem cell fate and maturation. *Dev Neurobiol*. 2012;72(7):1068-1084. doi:10.1002/dneu.22022

2. Costa C, Eixarch H, Martínez-Sáez E, et al. Expression of Bone Morphogenetic Proteins in Multiple Sclerosis Lesions. *Am J Pathol*. 2019;189(3):665-676. doi:10.1016/j.ajpath.2018.11.007

3. Kuznitsov-Yanovsky L, Shapira G, Gildin L, Shomron N, Ben-Yosef D. Transcriptomic Analysis of Human Fragile X Syndrome Neurons Reveals Neurite Outgrowth Modulation by the TGFβ/BMP Pathway. *Int J Mol Sci*. 2022;23(16):9278. doi:10.3390/ijms23169278

4. Mabie PC, Mehler MF, Marmur R, Papavasiliou A, Song Q, Kessler JA. Bone Morphogenetic Proteins Induce Astroglial Differentiation of Oligodendroglial–Astroglial Progenitor Cells. *J Neurosci*. 1997;17(11):4112-4120. doi:10.1523/JNEUROSCI.17-11-04112.1997

5. Grinspan JB, Edell E, Carpio DF, et al. Stage-specific effects of bone morphogenetic proteins on the oligodendrocyte lineage. *J Neurobiol*. 2000;43(1):1-17. doi:10.1002/(SICI)1097-4695(200004)43:1<1::AID-NEU1>3.0.CO;2-0

6. Petersen MA, Ryu JK, Chang KJ, et al. Fibrinogen activates BMP signaling in oligodendrocyte progenitor cells and inhibits remyelination after vascular damage. *Neuron*. 2017;96(5):1003. doi:10.1016/J.NEURON.2017.10.008

7. Li H, Grumet M. BMP and LIF signaling coordinately regulate lineage restriction of radial glia in the developing forebrain. *Glia*. 2007;55(1):24-35. doi:10.1002/glia.20434

8. Masjosthusmann S, Becker D, Petzuch B, et al. A transcriptome comparison of time-matched developing human, mouse and rat neural progenitor cells reveals human uniqueness. *Toxicol Appl Pharmacol*. 2018;354:40-55. doi:10.1016/j.taap.2018.05.009

9. Romano R, Bucci C. Role of EGFR in the Nervous System. *Cells*. 2020;9(8):1887. doi:10.3390/CELLS9081887

10. Koch K, Bartmann K, Hartmann J, et al. Scientific Validation of Human Neurosphere Assays for Developmental Neurotoxicity Evaluation. *Front Toxicol*. 2022;4:7. doi:10.3389/ftox.2022.816370

11. Tropepe V, Sibilia M, Ciruna BG, Rossant J, Wagner EF, Van Der Kooy D. Distinct neural stem cells proliferate in response to EGF and FGF in the developing mouse telencephalon. *Dev Biol*. 1999;208(1):166-188. doi:10.1006/dbio.1998.9192

12. Ayuso-Sacido A, Moliterno JA, Kratovac S, et al. Activated EGFR signaling increases proliferation, survival, and migration and blocks neuronal differentiation in post-natal neural stem cells. *J Neurooncol*. 2010;97(3):323–337. doi:10.1007/s11060-009-0035-x

13. Sun Y, Goderie SK, Temple S. Asymmetric Distribution of EGFR Receptor during Mitosis Generates Diverse CNS Progenitor Cells. *Neuron*. 2005;45(6):873-886. doi:10.1016/J.NEURON.2005.01.045

14. Martens DJ, Seaberg RM, van der Kooy D. In vivo infusions of exogenous growth factors into the fourth ventricle of the adult mouse brain increase the proliferation of neural progenitors around the fourth ventricle and the central canal of the spinal cord. *Eur J Neurosci*. 2002;16(6):1045-1057. doi:10.1046/J.1460-9568.2002.02181.X

15. Kojima A, Tator CH. Epidermal Growth Factor and Fibroblast Growth Factor 2 Cause Proliferation of Ependymal Precursor Cells in the Adult Rat Spinal Cord In Vivo. *J Neuropathol Exp Neurol*. 2000;59(8):687-697. doi:10.1093/JNEN/59.8.687

16. Ciccolini F, Mandl C, Hölzl-Wenig G, Kehlenbach A, Hellwig A. Prospective isolation of late development multipotent precursors whose migration is promoted by EGFR. *Dev Biol*. 2005;284(1):112-125. doi:10.1016/j.ydbio.2005.05.007

17. Caric D, Raphael H, Viti J, Feathers A, Wancio D, Lillien L. EGFRs mediate chemotactic migration in the developing telencephalon. *Development*. 2001;128(21):4203-4216. doi:10.1242/dev.128.21.4203

18. Ostenfeld T, Svendsen CN. Requirement for Neurogenesis to Proceed through the Division of Neuronal Progenitors following Differentiation of Epidermal Growth Factor and Fibroblast Growth Factor‐2–Responsive Human Neural Stem Cells. *Stem Cells*. 2004;22(5):798-811. doi:10.1634/stemcells.22-5-798

19. Palazuelos J, Crawford HC, Klingener M, et al. TACE/ADAM17 Is Essential for Oligodendrocyte Development and CNS Myelination. *J Neurosci*. 2014;34(36):11884-11896. doi:10.1523/JNEUROSCI.1220-14.2014

20. Sabino C, Bender D, Herrlein ML, Hildt E. The Epidermal Growth Factor Receptor Is a Relevant Host Factor in the Early Stages of The Zika Virus Life Cycle In Vitro. *J Virol*. 2021;95(20). doi:10.1128/JVI.01195-21

21. Carpentieri JA, Di Cicco A, Lampic M, et al. Endosomal trafficking defects alter neural progenitor proliferation and cause microcephaly. *Nat Commun*. 2022;13(1):16. doi:10.1038/s41467-021-27705-7

22. Tegenge MA, Rockel TD, Fritsche E, Bicker G. Nitric oxide stimulates human neural progenitor cell migration via cGMP-mediated signal transduction. *Cell Mol Life Sci*. 2011;68(12):2089-2099. doi:10.1007/S00018-010-0554-9

23. Delhaye S, Bardoni B. Role of phosphodiesterases in the pathophysiology of neurodevelopmental disorders. *Mol Psychiatry 2021 269*. 2021;26(9):4570-4582. doi:10.1038/s41380-020-00997-9

24. Tripathi MK, Ojha SK, Kartawy M, et al. The NO Answer for Autism Spectrum Disorder. *Adv Sci*. 2023;10(22):2205783. doi:10.1002/advs.202205783

25. Garthwaite J. Concepts of neural nitric oxide‐mediated transmission. *Eur J Neurosci*. 2008;27(11):2783-2802. doi:10.1111/j.1460-9568.2008.06285.x

26. Hollas MA, Ben Aissa M, Lee SH, Gordon-Blake JM, Thatcher GRJ. Pharmacological manipulation of cGMP and NO/cGMP in CNS drug discovery. *Nitric Oxide*. 2019;82:59-74. doi:10.1016/j.niox.2018.10.006

27. Tremblay M, Fugère V, Tsui J, et al. Regulation of Radial Glial Motility by Visual Experience. *J Neurosci*. 2009;29(45):14066-14076. doi:10.1523/JNEUROSCI.3542-09.2009

28. Contestabile A, Monti B, Polazzi E. Neuronal-glial Interactions Define the Role of Nitric Oxide in Neural Functional Processes. *Curr Neuropharmacol*. 2012;10(4):303-310. doi:10.2174/157015912804143522

29. Uhlén M, Fagerberg L, Hallström BM, et al. Tissue-based map of the human proteome. *Science (80- )*. 2015;347(6220). doi:10.1126/SCIENCE.1260419/SUPPL_FILE/1260419_UHLEN.SM.PDF

30. Singh RK, Kumar S, Gautam PK, et al. Protein kinase C-α and the regulation of diverse cell responses. *Biomol Concepts*. 2017;8(3-4):143-153. doi:10.1515/BMC-2017-0005/ASSET/GRAPHIC/J_BMC-2017-0005_FIG_005.JPG

31. Calabrese B, Halpain S. Essential Role for the PKC Target MARCKS in Maintaining Dendritic Spine Morphology. *Neuron*. 2005;48(1):77-90. doi:10.1016/j.neuron.2005.08.027

32. Hundle B, McMahon T, Dadgar J, Messing RO. Overexpression of ∊-Protein Kinase C Enhances Nerve Growth Factor-induced Phosphorylation of Mitogen-activated Protein Kinases and Neurite Outgrowth. *Cell Biol Metab*. 1995;270(50):30134-30140. doi:10.1074/jbc.270.50.30134

33. Tsuji M, Inanami O, Kuwabara M. Induction of Neurite Outgrowth in PC12 Cells by α-Phenyl-N-tert-butylnitron through Activation of Protein Kinase C and the Ras-Extracellular Signal-regulated Kinase Pathway. *J Biol Chem*. 2001;276(35):32779-32785. doi:10.1074/jbc.M101403200

34. Kawano T, Inokuchi J, Eto M, Murata M, Kang JH. Activators and Inhibitors of Protein Kinase C (PKC): Their Applications in Clinical Trials. *Pharmaceutics*. 2021;13(11):1748. doi:10.3390/pharmaceutics13111748

35. Toullec D, Pianetti P, Coste H, et al. The bisindolylmaleimide GF 109203X is a potent and selective inhibitor of protein kinase C. *J Biol Chem*. 1991;266(24):15771-15781. doi:10.1016/S0021-9258(18)98476-0

36. Masjosthusmann S, Blum J, Bartmann K, et al. *Establishment of an a Priori Protocol for the Implementation and Interpretation of an In‐vitro Testing Battery for the Assessment of Developmental Neurotoxicity*.; 2020. doi:10.2903/sp.efsa.2020.en-1938

37. Stiegler N V., Krug AK, Matt F, Leist M. Assessment of Chemical-Induced Impairment of Human Neurite Outgrowth by Multiparametric Live Cell Imaging in High-Density Cultures. *Toxicol Sci*. 2011;121(1):73-87. doi:10.1093/toxsci/kfr034

38. Imai F, Hirai SI, Akimoto K, et al. Inactivation of aPKCλ results in the loss of adherens junctions in neuroepithelial cells without affecting neurogenesis in mouse neocortex. *Development*. 2006;133(9):1855-1855. doi:10.1242/dev.02389

39. Wu QW, Kapfhammer JP. The Emerging Key Role of the mGluR1-PKCγ Signaling Pathway in the Pathogenesis of Spinocerebellar Ataxias: A Neurodevelopmental Viewpoint. *Int J Mol Sci*. 2022;23(16):9169. doi:10.3390/ijms23169169

40. Harris TJC, Tepass U. Adherens junctions: from molecules to morphogenesis. *Nat Rev Mol Cell Biol*. 2010;11(7):502-514. doi:10.1038/nrm2927

41. Arnold TR, Stephenson RE, Miller AL. Rho GTPases and Actomyosin: partners in regulating epithelial cell-cell junction structure and function. *Exp Cell Res*. 2017;358(1):20. doi:10.1016/J.YEXCR.2017.03.053

42. Binamé F, Sakry D, Dimou L, Jolivel V, Trotter J. NG2 regulates directional migration of oligodendrocyte precursor cells via Rho GTPases and polarity complex proteins. *J Neurosci*. 2013;33(26):10858-10874. doi:10.1523/JNEUROSCI.5010-12.2013

43. Zamboni V, Jones R, Umbach A, et al. Rho GTPases in Intellectual Disability: From Genetics to Therapeutic Opportunities. *Int J Mol Sci*. 2018;19(6):1821. doi:10.3390/ijms19061821

44. Anijs M, Devanna P, Vernes SC. ARHGEF39, a Gene Implicated in Developmental Language Disorder, Activates RHOA and Is Involved in Cell De-Adhesion and Neural Progenitor Cell Proliferation. *Front Mol Neurosci*. 2022;15:39. doi:10.3389/fnmol.2022.941494

45. Scala M, Nishikawa M, Nagata K ichi, Striano P. Pathophysiological Mechanisms in Neurodevelopmental Disorders Caused by Rac GTPases Dysregulation: What’s behind Neuro-RACopathies. *Cells*. 2021;10(12):3395. doi:10.3390/cells10123395

46. Benarroch E. What Is the Role of the Rho-ROCK Pathway in Neurologic Disorders? *Neurology*. 2023;101(12):536-543. doi:10.1212/WNL.0000000000207779

47. Pedraza CE, Taylor C, Pereira A, et al. Induction of Oligodendrocyte Differentiation and In Vitro Myelination by Inhibition of Rho-Associated Kinase. *ASN Neuro*. 2014;6(4):175909141453813. doi:10.1177/1759091414538134

48. Nuovo S, Brankovic V, Caputi C, et al. Novel unconventional variants expand the allelic spectrum of <scp> *OPHN1* </scp> gene. *Am J Med Genet Part A*. 2021;185(5):1575-1581. doi:10.1002/ajmg.a.62144

49. Compagnucci C, Barresi S, Petrini S, et al. Rho Kinase Inhibition Is Essential During In Vitro Neurogenesis and Promotes Phenotypic Rescue of Human Induced Pluripotent Stem Cell-Derived Neurons With Oligophrenin-1 Loss of Function. *Stem Cells Transl Med*. 2016;5(7):860-869. doi:10.5966/sctm.2015-0303

50. Pillet LE, Cresto N, Saillour Y, et al. The intellectual disability protein Oligophrenin-1 controls astrocyte morphology and migration. *Glia*. 2020;68(9):1729-1742. doi:10.1002/glia.23801

51. Ohnishi H, Murata Y, Okazawa H, Matozaki T. Src family kinases: modulators of neurotransmitter receptor function and behavior. *Trends Neurosci*. 2011;34(12):629-637. doi:10.1016/j.tins.2011.09.005

52. Socodato R, Portugal CC, Domith I, et al. c‐Src function is necessary and sufficient for triggering microglial cell activation. *Glia*. 2015;63(3):497-511. doi:10.1002/glia.22767

53. Portugal CC, Almeida TO, Socodato R, Relvas JB. Src family kinases (SFKs): critical regulators of microglial homeostatic functions and neurodegeneration in Parkinson’s and Alzheimer’s diseases. *FEBS J*. Published online December 1, 2021. doi:10.1111/FEBS.16197

54. Moors M, Cline JE, Abel J, Fritsche E. ERK-dependent and -independent pathways trigger human neural progenitor cell migration. *Toxicol Appl Pharmacol*. 2007;221(1):57-67. doi:10.1016/j.taap.2007.02.018

55. Stamenkovic V, Lautz JD, Harsh FM, Smith SEP. SRC family kinase inhibition rescues molecular and behavioral phenotypes, but not protein interaction network dynamics, in a mouse model of Fragile X syndrome. *Mol Psychiatry*. 2024;29(5):1392-1405. doi:10.1038/s41380-024-02418-7

56. Snyder MA, Gao WJ. NMDA hypofunction as a convergence point for progression and symptoms of schizophrenia. *Front Cell Neurosci*. 2013;7(MAR):41853. doi:10.3389/fncel.2013.00031

57. Kim B, Koh Y, Do H, et al. Aberrant Cortical Layer Development of Brain Organoids Derived from Noonan Syndrome-iPSCs. *Int J Mol Sci*. 2022;23(22):13861. doi:10.3390/ijms232213861

58. Blum J, Masjosthusmann S, Bartmann K, et al. Establishment of a human cell-based in vitro battery to assess developmental neurotoxicity hazard of chemicals. *Chemosphere*. 2023;311(July 2022):137035. doi:10.1016/j.chemosphere.2022.137035

59. Chen SH, Sung YF, Oyarzabal EA, et al. Physiological Concentration of Prostaglandin E2 Exerts Anti-inflammatory Effects by Inhibiting Microglial Production of Superoxide Through a Novel Pathway. *Mol Neurobiol*. 2018;55(10):8001-8013. doi:10.1007/s12035-018-0965-4

60. Kamal DAM, Abidin SZ, Saudi WSW, Kumar J, Bellato A. Roles of Prostaglandins and Cyclooxygenases in Autism Spectrum Disorder: A Comprehensive Review. *Curr Behav Neurosci Reports*. 2025;12(1):2. doi:10.1007/s40473-024-00294-7

61. Goldenberg MM. Celecoxib, a selective cyclooxygenase-2 inhibitor for the treatment of rheumatoid arthritis and osteoarthritis. *Clin Ther*. 1999;21(9):1497-1513. doi:10.1016/S0149-2918(00)80005-3

62. Mironov S, Skorova E, Taschenberger G, et al. Imaging cytoplasmic cAMP in mouse brainstem neurons. *BMC Neurosci*. 2009;10(1):29. doi:10.1186/1471-2202-10-29

63. Wang H, Xu J, Lazarovici P, Quirion R, Zheng W. cAMP Response Element-Binding Protein (CREB): A Possible Signaling Molecule Link in the Pathophysiology of Schizophrenia. *Front Mol Neurosci*. 2018;11:255. doi:10.3389/fnmol.2018.00255

64. Takahashi M, Li Y, Dillon TJ, Stork PJS. Phosphorylation of Rap1 by cAMP-dependent Protein Kinase (PKA) Creates a Binding Site for KSR to Sustain ERK Activation by cAMP. *J Biol Chem*. 2017;292(4):1449-1461. doi:10.1074/jbc.M116.768986

65. Steven A, Friedrich M, Jank P, et al. What turns CREB on? And off? And why does it matter? *Cell Mol Life Sci*. 2020;77(20):4049-4067. doi:10.1007/s00018-020-03525-8

66. Heinz S, Freyberger A, Lawrenz B, Schladt L, Schmuck G, Ellinger-Ziegelbauer H. Mechanistic Investigations of the Mitochondrial Complex I Inhibitor Rotenone in the Context of Pharmacological and Safety Evaluation. *Sci Rep*. 2017;7(1):45465. doi:10.1038/srep45465

67. Terron A, Bal-Price A, Paini A, et al. An adverse outcome pathway for parkinsonian motor deficits associated with mitochondrial complex I inhibition. *Arch Toxicol*. 2018;92(1):41-82. doi:10.1007/s00204-017-2133-4

68. Bisbal M, Remedi M, Quassollo G, Cáceres A, Sanchez M. Rotenone inhibits axonogenesis via an Lfc/RhoA/ROCK pathway in cultured hippocampal neurons. *J Neurochem*. 2018;146(5):570-584. doi:10.1111/jnc.14547

69. Bisbal M, Sanchez M. Neurotoxicity of the pesticide rotenone on neuronal polarization: a mechanistic approach. *Neural Regen Res*. 2019;14(5):762. doi:10.4103/1673-5374.249847

70. Choi YJ, Park YJ, Park JY, et al. Inhibitory Effect of mTOR Activator MHY1485 on Autophagy: Suppression of Lysosomal Fusion. Tajmir-Riahi HA, ed. *PLoS One*. 2012;7(8):e43418. doi:10.1371/journal.pone.0043418

71. Beck JT, Ismail A, Tolomeo C. Targeting the phosphatidylinositol 3-kinase (PI3K)/AKT/mammalian target of rapamycin (mTOR) pathway: An emerging treatment strategy for squamous cell lung carcinoma. *Cancer Treat Rev*. 2014;40(8):980-989. doi:10.1016/j.ctrv.2014.06.006

72. Huang X yi, Hu Q peng, Shi H yun, Zheng Y yu, Hu R rong, Guo Q. Everolimus inhibits PI3K/Akt/mTOR and NF-kB/IL-6 signaling and protects seizure-induced brain injury in rats. *J Chem Neuroanat*. 2021;114:101960. doi:10.1016/j.jchemneu.2021.101960

73. Hashimoto-Torii K, Torii M, Sarkisian MR, et al. Interaction between Reelin and Notch Signaling Regulates Neuronal Migration in the Cerebral Cortex. *Neuron*. 2008;60(2):273-284. doi:10.1016/j.neuron.2008.09.026

74. Bock HH, May P. Canonical and Non-canonical Reelin Signaling. *Front Cell Neurosci*. 2016;10(Jun):195731. doi:10.3389/fncel.2016.00166

75. Jossin Y. Reelin Functions, Mechanisms of Action and Signaling Pathways During Brain Development and Maturation. *Biomolecules*. 2020;10(6):964. doi:10.3390/biom10060964

76. Zhang HM, Liu P, Jiang C, et al. Notch signaling inhibitor DAPT provides protection against acute craniocerebral injury. Boltze J, ed. *PLoS One*. 2018;13(2):e0193037. doi:10.1371/journal.pone.0193037

77. Hans CP, Sharma N, Dev R, Blain JM, Tonniges J, Agarwal G. DAPT, a potent Notch inhibitor regresses actively growing abdominal aortic aneurysm via divergent pathways. *Clin Sci*. 2020;134(12):1555-1572. doi:10.1042/CS20200456

78. Dong Z, Huo J, Liang A, Chen J, Chen G, Liu D. Gamma-Secretase Inhibitor (DAPT), a potential therapeutic target drug, caused neurotoxicity in planarian regeneration by inhibiting Notch signaling pathway. *Sci Total Environ*. 2021;781:146735. doi:10.1016/j.scitotenv.2021.146735

79. Hur JY. γ-Secretase in Alzheimer’s disease. *Exp Mol Med*. 2022;54(4):433-446. doi:10.1038/s12276-022-00754-8

80. Sil S, Periyasamy P, Thangaraj A, Chivero ET, Buch S. PDGF/PDGFR axis in the neural systems. *Mol Aspects Med*. 2018;62:63-74. doi:10.1016/j.mam.2018.01.006

81. Li D, Huang LT, Zhang C pu, Li Q, Wang JH. Insights Into the Role of Platelet-Derived Growth Factors: Implications for Parkinson’s Disease Pathogenesis and Treatment. *Front Aging Neurosci*. 2022;14:890509. doi:10.3389/fnagi.2022.890509

82. Roberts WG, Whalen PM, Soderstrom E, et al. Antiangiogenic and Antitumor Activity of a Selective PDGFR Tyrosine Kinase Inhibitor, CP-673,451. *Cancer Res*. 2005;65(3):957-966. doi:10.1158/0008-5472.957.65.3

83. Jo H, Mondal S, Tan D, et al. Small molecule-induced cytosolic activation of protein kinase Akt rescues ischemia-elicited neuronal death. *Proc Natl Acad Sci*. 2012;109(26):10581-10586. doi:10.1073/pnas.1202810109

84. Zhu J liang, Wu Y ying, Wu D, Luo WF, Zhang Z qing, Liu C feng. SC79, a novel Akt activator, protects dopaminergic neuronal cells from MPP+ and rotenone. *Mol Cell Biochem*. 2019;461(1-2):81-89. doi:10.1007/s11010-019-03592-x

85. Gharbi SI, Zvelebil MJ, Shuttleworth SJ, et al. Exploring the specificity of the PI3K family inhibitor LY294002. *Biochem J*. 2007;404(1):15-21. doi:10.1042/BJ20061489

86. Brunn GJ, Williams J, Sabers C, Wiederrecht G, Lawrence JC, Abraham RT. Direct inhibition of the signaling functions of the mammalian target of rapamycin by the phosphoinositide 3-kinase inhibitors, wortmannin and LY294002. *EMBO J*. 1996;15(19):5256-5267. doi:10.1002/j.1460-2075.1996.tb00911.x

87. Davies SP, Reddy H, Caivano M, Cohen P. Specificity and mechanism of action of some commonly used protein kinase inhibitors. *Biochem J*. 2000;351(1):95. doi:10.1042/0264-6021:3510095

88. Bae YS, Lee TG, Park JC, et al. Identification of a compound that directly stimulates phospholipase C activity. *Mol Pharmacol*. 2003;63(5):1043-1050. doi:10.1124/mol.63.5.1043

89. Krjukova J, Holmqvist T, Danis AS, Åkerman KEO, Kukkonen JP. Phospholipase C activator m ‐3M3FBS affects Ca 2+ homeostasis independently of phospholipase C activation. *Br J Pharmacol*. 2004;143(1):3-7. doi:10.1038/sj.bjp.0705911

90. Bleasdale JE, Thakur NR, Gremban RS, et al. Selective inhibition of receptor-coupled phospholipase C-dependent processes in human platelets and polymorphonuclear neutrophils. *J Pharmacol Exp Ther*. 1990;255(2):756-768. doi:10.1016/S0022-3565(25)22967-6

91. Bala GA, Thakur NR, Bleasdale JE. Characterization of the Major Phosphoinositide-specific Phospholipase C of Human Amnion. *Biol Reprod*. 1990;43(4):704-711. doi:10.1095/biolreprod43.4.704

92. Bleasdale JE, Fisher SK. Use of U-73122 as an Inhibitor of Phospholipase C-Dependent Processes. *Neuroprotocols*. 1993;3(2):125-133. doi:10.1006/ncmn.1993.1046

93. Huang W, Barrett M, Hajicek N, et al. Small Molecule Inhibitors of Phospholipase C from a Novel High-throughput Screen. *J Biol Chem*. 2013;288(8):5840-5848. doi:10.1074/jbc.M112.422501

94. Klein RR, Bourdon DM, Costales CL, et al. Direct Activation of Human Phospholipase C by Its Well Known Inhibitor U73122. *J Biol Chem*. 2011;286(14):12407-12416. doi:10.1074/jbc.M110.191783

95. Chiba T, Yamada M, Hashimoto Y, et al. Development of a Femtomolar-Acting Humanin Derivative Named Colivelin by Attaching Activity-Dependent Neurotrophic Factor to Its N Terminus: Characterization of Colivelin-Mediated Neuroprotection against Alzheimer’s Disease-Relevant Insults In Vitro and I. *J Neurosci*. 2005;25(44):10252-10261. doi:10.1523/JNEUROSCI.3348-05.2005

96. Chiba T, Nishimoto I, Aiso S, Matsuoka M. Neuroprotection against neurodegenerative diseases. *Mol Neurobiol*. 2007;35(1):55-84. doi:10.1007/BF02700624

97. Liu S, Zhang S, Lv X, et al. Limonin ameliorates ulcerative colitis by regulating STAT3/miR-214 signaling pathway. *Int Immunopharmacol*. 2019;75:105768. doi:10.1016/j.intimp.2019.105768

98. Chen J, Liu BX, Shen Q, et al. Limonin inhibits angiogenesis and metastasis of human breast cancer cells by suppressing the VEGFR2/IGFR1-mediated STAT3 signaling pathway. *Transl Cancer Res*. 2020;9(11):6820-6832. doi:10.21037/tcr-20-1992

99. Zhang WF, Ruan CW, Wu JB, Wu GL, Wang XG, Chen HJ. Limonin inhibits the stemness of cancer stem-like cells derived from colorectal carcinoma cells potentially via blocking STAT3 signaling. *World J Clin Oncol*. 2024;15(2):317-328. doi:10.5306/wjco.v15.i2.317

100. Lee ST, Lee JY, Kim HE, Park JY, Choi JK. Limonin Exhibits Anti-Inflammatory Effects by Inhibiting mTORC1 and Mitochondrial Reactive Oxygen Species in Psoriatic-like Skin Inflammation. *Antioxidants*. 2024;13(12):1541. doi:10.3390/antiox13121541

101. Xiong W, Feng S, Wang H, et al. Identification of candidate genes and pathways in limonin-mediated cardiac repair after myocardial infarction. *Biomed Pharmacother*. 2021;142:112088. doi:10.1016/j.biopha.2021.112088

102. Yang R, Yu H, Chen J, et al. Limonin Attenuates LPS-Induced Hepatotoxicity by Inhibiting Pyroptosis via NLRP3/Gasdermin D Signaling Pathway. *J Agric Food Chem*. 2021;69(3):982-991. doi:10.1021/acs.jafc.0c06775

103. Wang Y, Liang J, Xu B, Yang J, Wu Z, Cheng L. TrkB/BDNF signaling pathway and its small molecular agonists in CNS injury. *Life Sci*. 2024;336:122282. doi:10.1016/j.lfs.2023.122282

104. Cazorla M, Prémont J, Mann A, Girard N, Kellendonk C, Rognan D. Identification of a low–molecular weight TrkB antagonist with anxiolytic and antidepressant activity in mice. *J Clin Invest*. 2011;121(5):1846-1857. doi:10.1172/JCI43992

105. Law SM, Zheng JJ. Premise and peril of Wnt signaling activation through GSK-3β inhibition. *iScience*. 2022;25(4):104159. doi:10.1016/j.isci.2022.104159

106. Chen B, Dodge ME, Tang W, et al. Small molecule–mediated disruption of Wnt-dependent signaling in tissue regeneration and cancer. *Nat Chem Biol*. 2009;5(2):100-107. doi:10.1038/nchembio.137

107. García-Reyes B, Witt L, Jansen B, et al. Discovery of Inhibitor of Wnt Production 2 (IWP-2) and Related Compounds As Selective ATP-Competitive Inhibitors of Casein Kinase 1 (CK1) δ/ε. *J Med Chem*. 2018;61(9):4087-4102. doi:10.1021/acs.jmedchem.8b00095

# No-effect results of specific endpoints

**Table S4 List of chemicals used to modulate the investigated pathways.**

Detailed information can be found in the main publication in Table 3.

| **Reagent** | **Pathway** |
| --- | --- |
| **PGE2** | COX-2 activator |
| **Celecoxib** | COX-2 inhibitor |
| **cAMP** | CREB activator |
| **KG-501** | CREB inhibitor |
| **Rotenone** | ETC I inhibitor |
| **MHY1485** | mTOR activator |
| **Everolimus** | mTOR inhibitor |
| **Reelin** | Notch activator |
| **DAPT** | Notch inhibitor |
| **hPDGF** | PDGFR activator |
| **CP-673451** | PDGFR inhibitor |
| **SC79** | PI3K-Akt activator |
| **LY294002** | PI3K-Akt inhibitor |
| **m-3M3FBS** | PLC activator |
| **U73122** | PLC inhibitor |
| **Colivelin** | STAT3 activator |
| **Limonin** | STAT3 inhibitor |
| **BDNF** | TrkB activator |
| **ANA-12** | TrkB inhibitor |
| **CHIR** | WNT activator |
| **IWP2** | WNT inhibitor |

The results of the ANA-12 modulator are not shown. As explained in the main manuscript file in the discussion section, BDNF is not expressed in our cultures, thus the natural TrkB activation did not take place which made the inhibition of an activated pathway using ANA-12 impossible.

Cytotoxic concentrations assessed using the LDH assay were excluded from the analysis and therefore these results are not plotted into figures. The raw experimental data are available at BioStudies ONTOX collection under accession numbers starting at S-ONTX37 until S-ONTX57 available at <https://www.ebi.ac.uk/biostudies/ONTOX/studies>.

## Figure S3 - NPC1

The NPC1 assay assesses NPC proliferation. Primary hNPCs were exposed for 3 days to increasing concentrations of activators and inhibitors of the selected signaling pathways in a proliferation medium containing growth factors EGF and FGF. Proliferation was assessed by BrdU incorporation into the DNA and shown as a percentage compared to the solvent control. Data are presented as mean ± SEM with dotted lines representing the lower and upper limit confidence intervals. Only no-effect results are shown in these supplemental files, see Figure 3 of the main manuscript for the effect results.

## NPC2a

The NPC2a assay assesses RG migration. Primary hNPCs were exposed for 5 days to increasing concentrations of activators and inhibitors of the selected signaling pathways in a differentiation medium without growth factors on PDL-laminin-coated plates. RG migration was assessed by defining the area of Hoechst33258-stained nuclei of the radially migrated cells out of the sphere core as a percentage compared to the solvent control migration area. Data are presented as mean ± SEM with dotted lines representing the lower and upper limit confidence intervals. Only no-effect results are shown in these supplemental files, see Figure 4 of the main manuscript for the effect results.


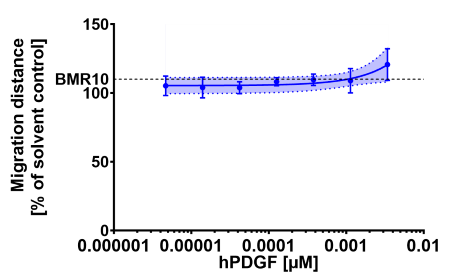

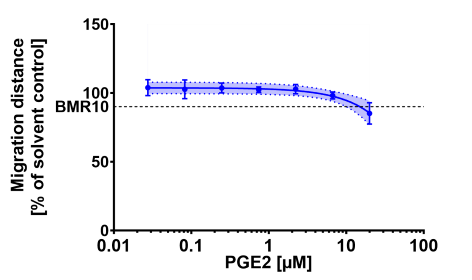

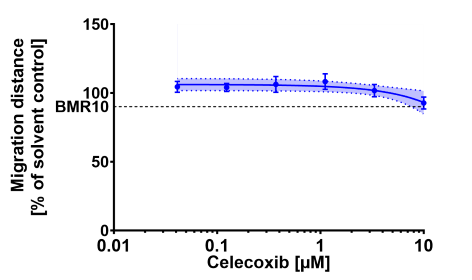


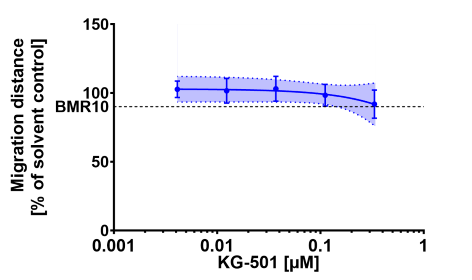


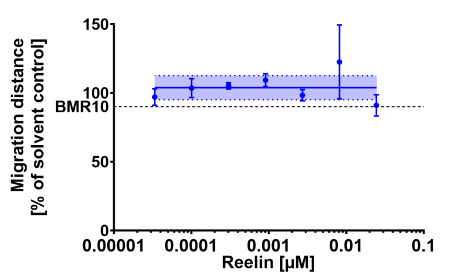

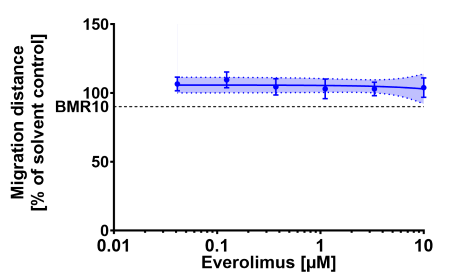


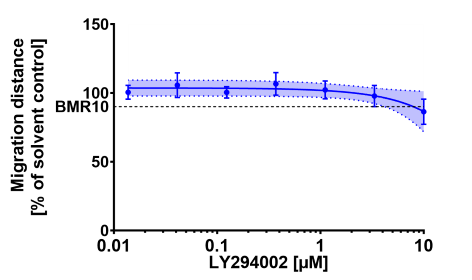

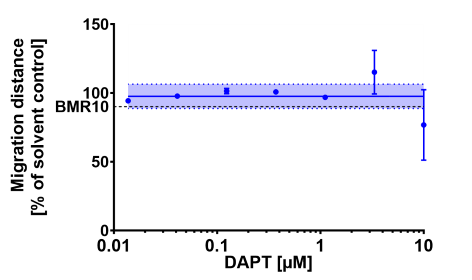


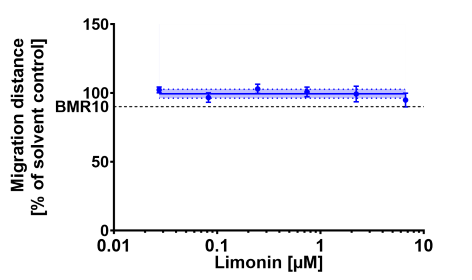

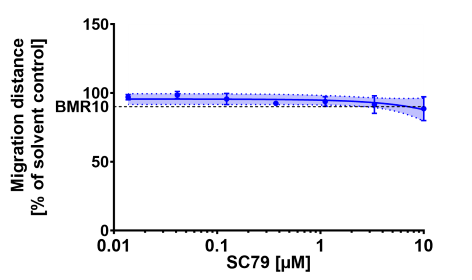


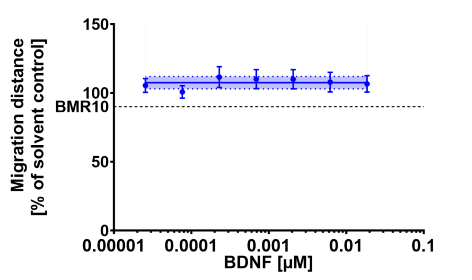

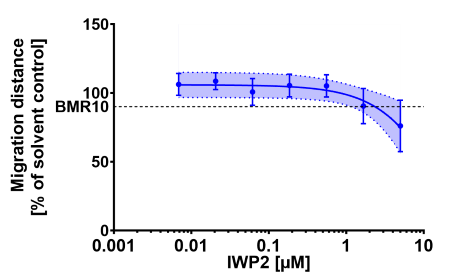

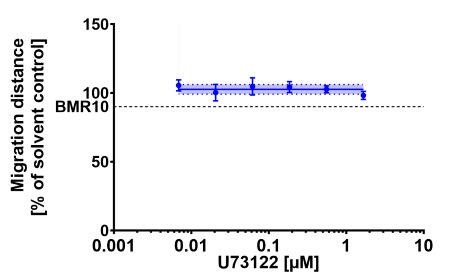


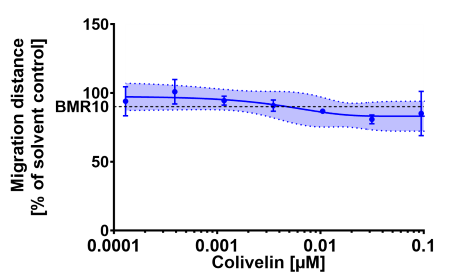


## NPC2b

The NPC2b assay assesses neuronal migration. Primary hNPCs were exposed for 5 days to increasing concentrations of activators and inhibitors of the selected signaling pathways in a differentiation medium without growth factors on PDL-laminin-coated plates. Migration was assessed as the mean distance of all neurons within the migration area divided by the radial glia migration distance and shown as a percentage compared to the solvent control. Data are presented as mean ± SEM with dotted lines representing the lower and upper limit confidence intervals. Neuronal migration was not affected by any of the tested pathway modulators.


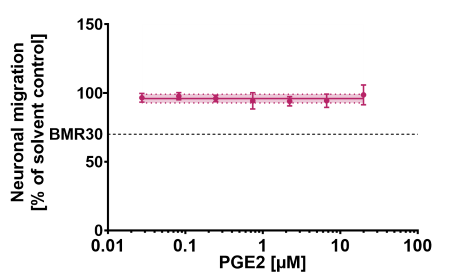

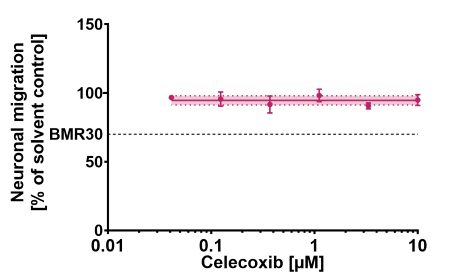


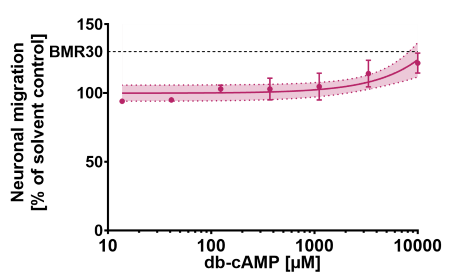

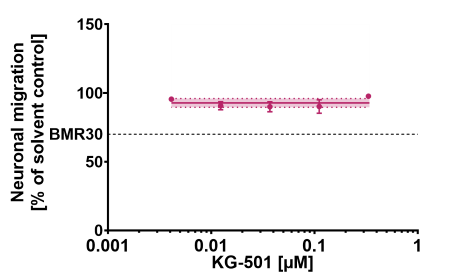


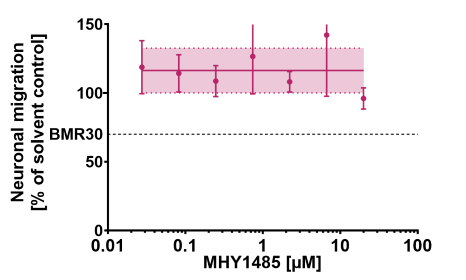

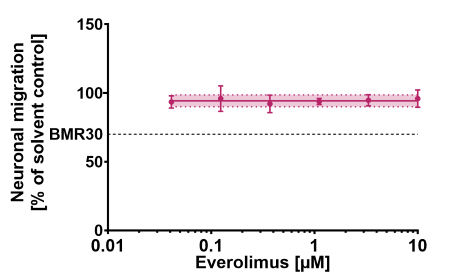


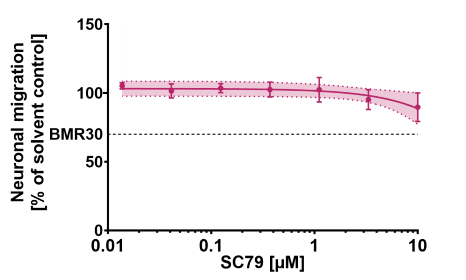

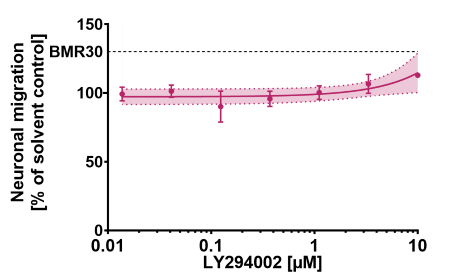


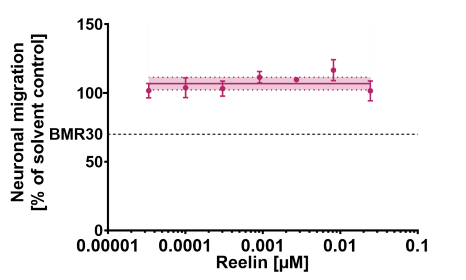

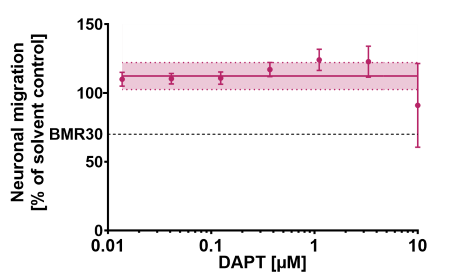


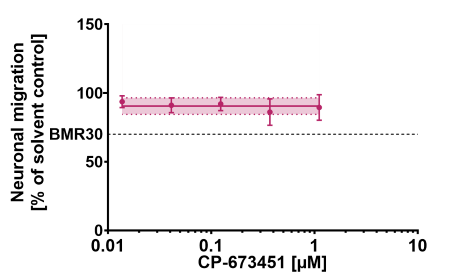

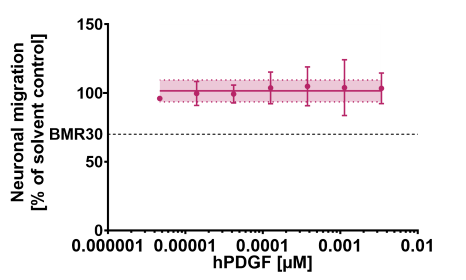


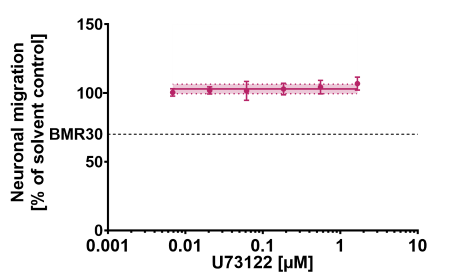

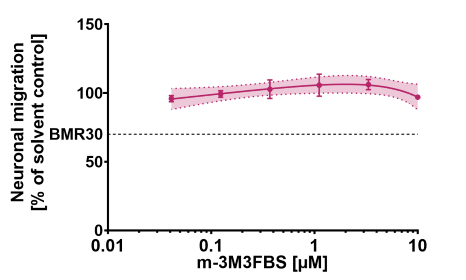


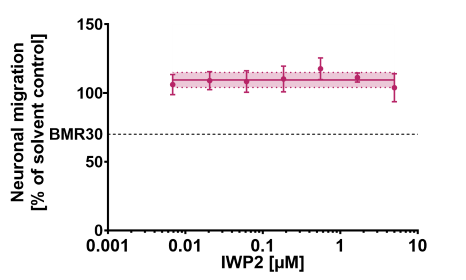


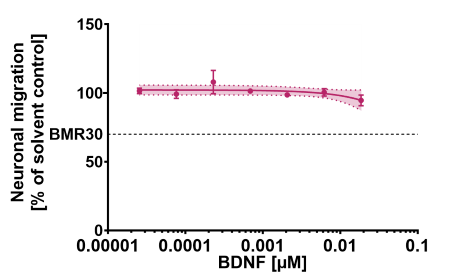

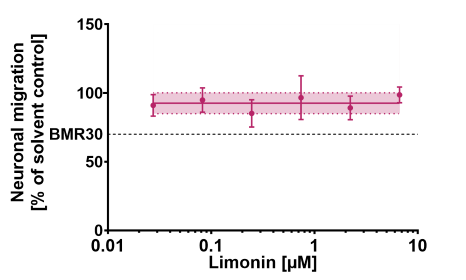


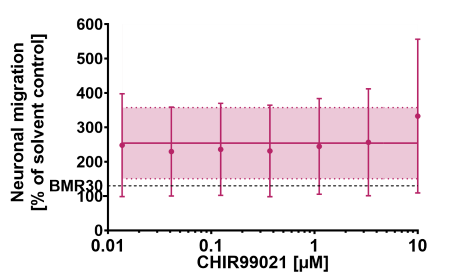


## NPC2c

The NPC2c assay assesses oligodendrocyte migration. Primary human neural progenitor cells were exposed for 5 days to increasing concentrations of activators and inhibitors of the selected signaling pathways in a differentiation medium without growth factors on PDL-laminin-coated plates. Migration was assessed as the mean distance of all oligodendrocytes within the migration area divided by the radial glia migration distance and shown as a percentage compared to the solvent control. Data are presented as mean ± SEM with dotted lines representing the lower and upper limit confidence intervals. Only no-effect results are shown in these supplemental files, see Supplemental Figure S1 for the effect results.


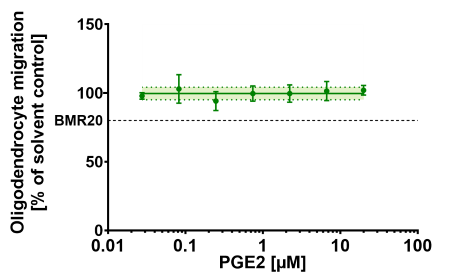

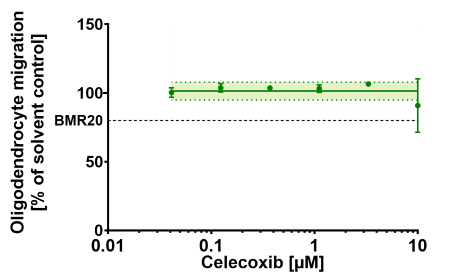


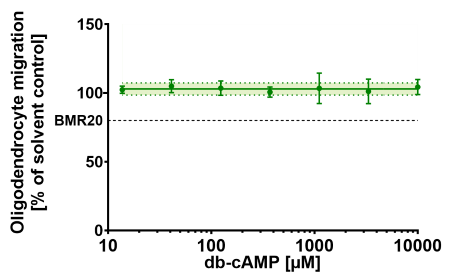

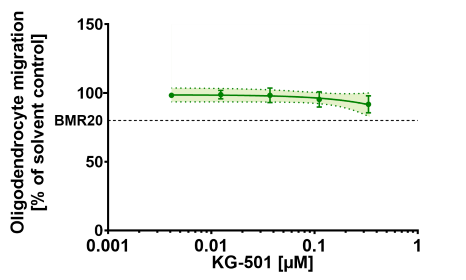


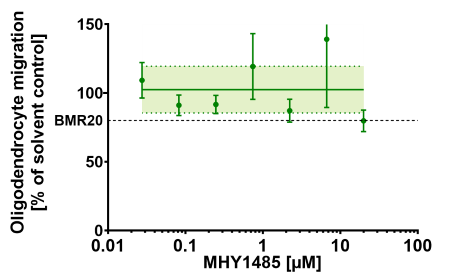

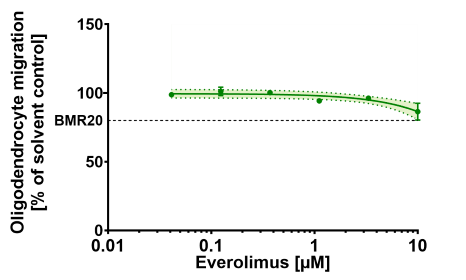


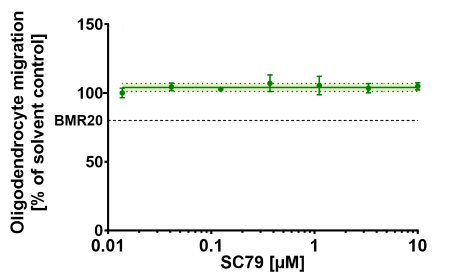

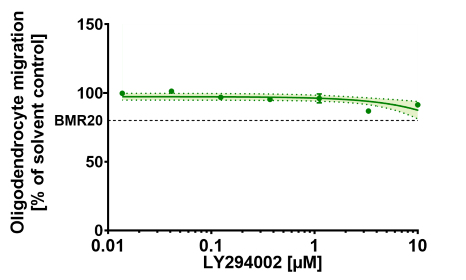


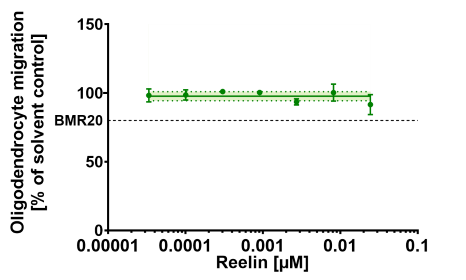

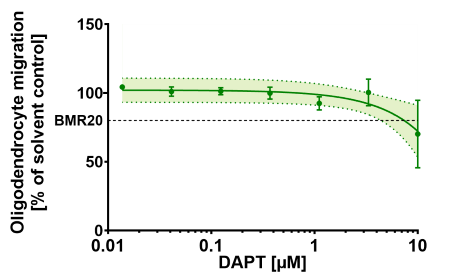


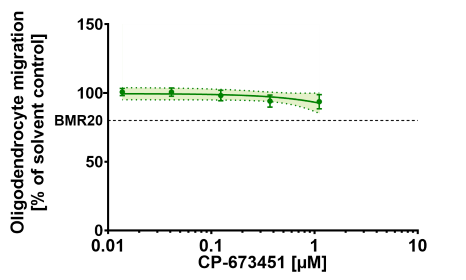

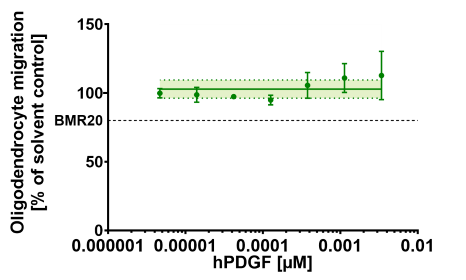


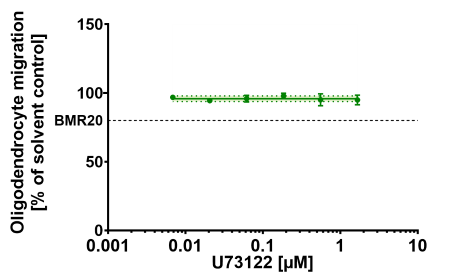

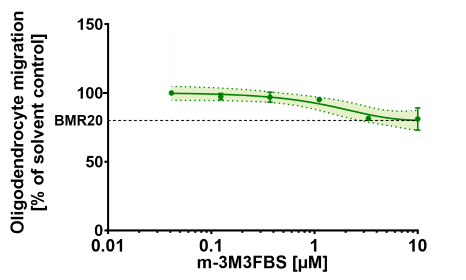


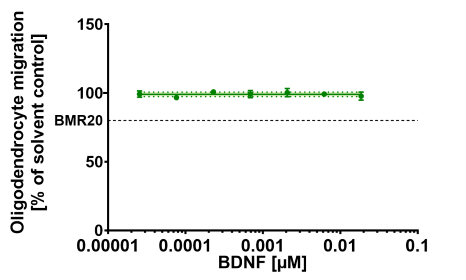

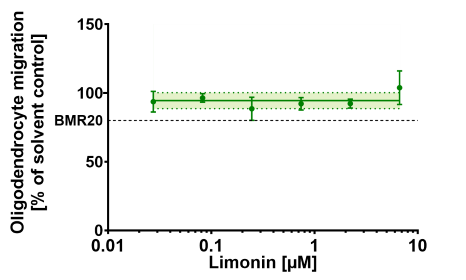

## NPC3

The NPC3 assay assesses neuronal differentiation. Primary hNPCs were exposed for 5 days to increasing concentrations of activators and inhibitors of the selected signaling pathways in a differentiation medium without growth factors on PDL-laminin-coated plates. Neuronal differentiation was assessed as the percentage of β(III)tubulin-positive neurons compared to the total nuclei count within the migration area. Data are presented as mean ± SEM with dotted lines representing the lower and upper limit confidence intervals. Only no-effect results are shown in these supplemental files, see Figure 5 of the main manuscript for the effect results.


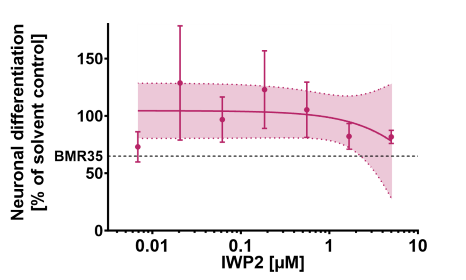

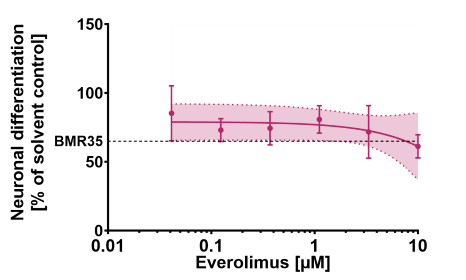


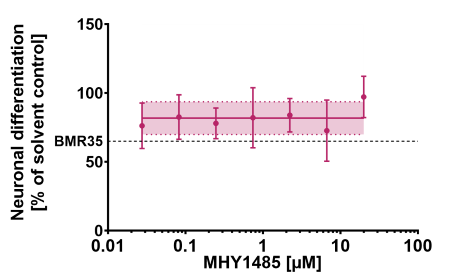

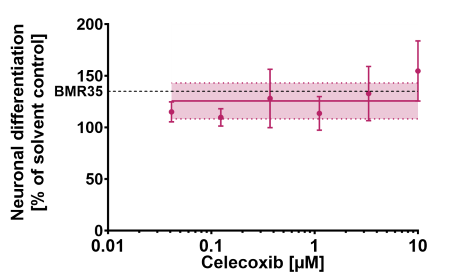


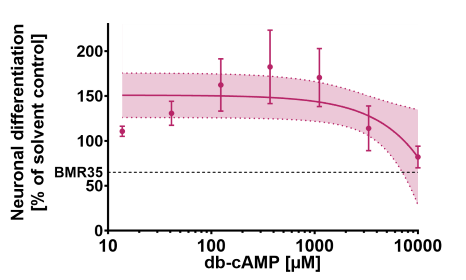

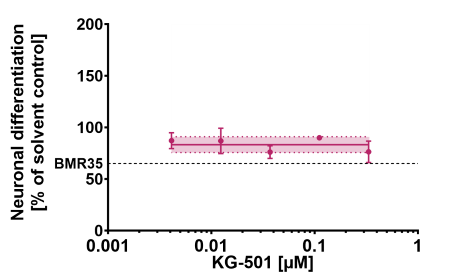

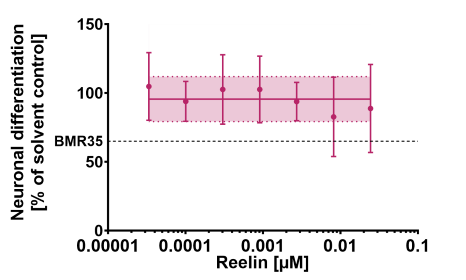

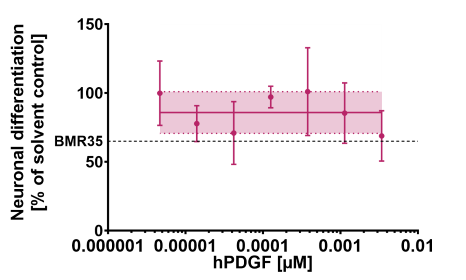


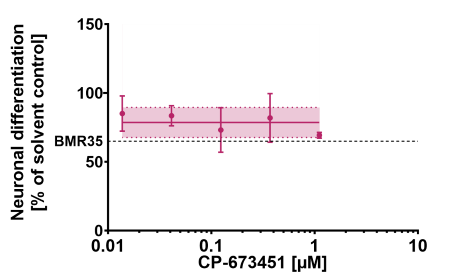

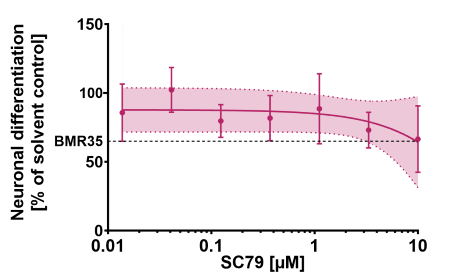

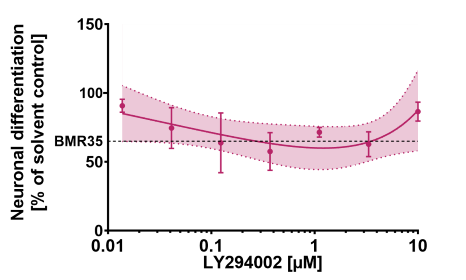

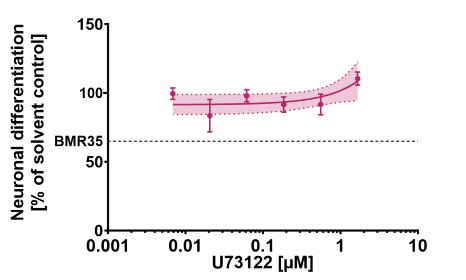

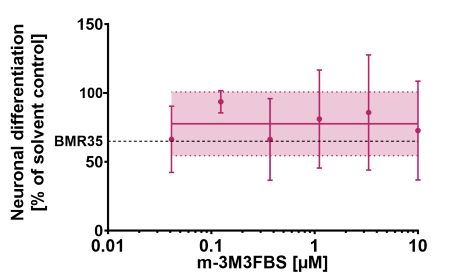

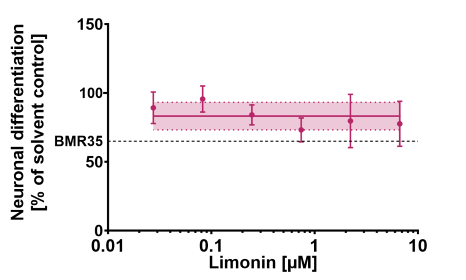

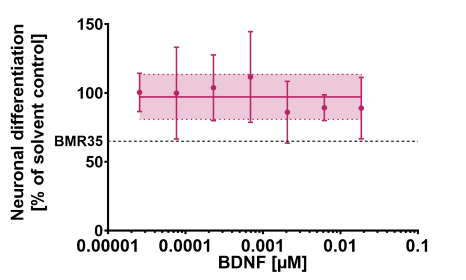


## NPC4

Primary hNPCs were exposed for 5 days to increasing concentrations of activators and inhibitors of the selected signaling pathways in a differentiation medium without growth factors on PDL-laminin-coated plates. Neurite outgrowth was assessed as neurite length (µm) and neurite area (pixel) of all β(III)tubulin-positive neurons. Data are presented as mean ± SEM with dotted lines representing the lower and upper limit confidence intervals. Only no-effect results are shown in these supplemental files, see Figure 6 of the main manuscript for the effect results.


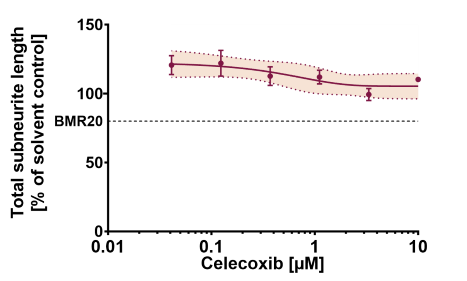

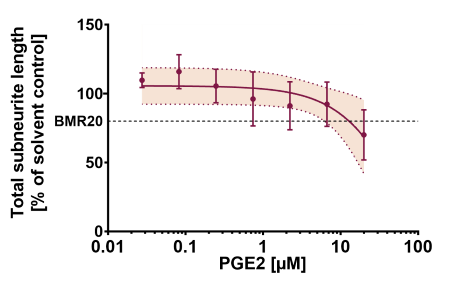


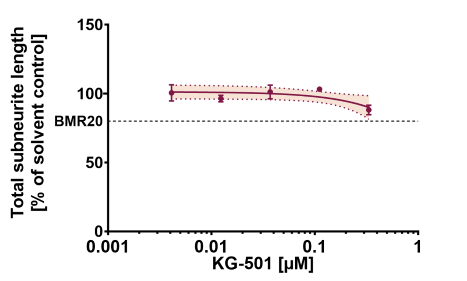


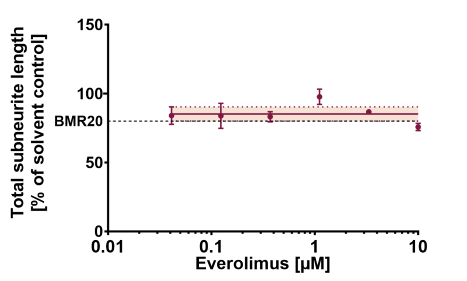

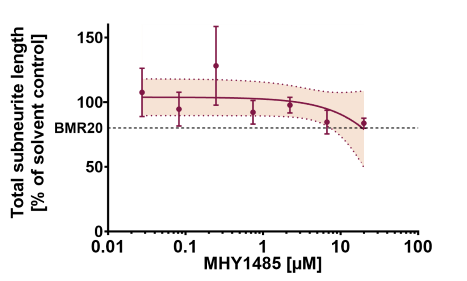


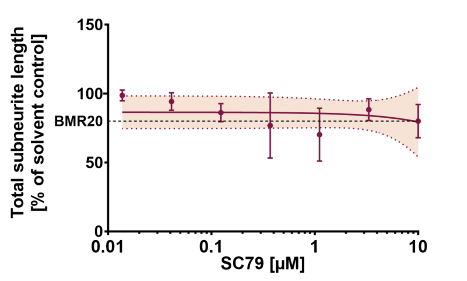

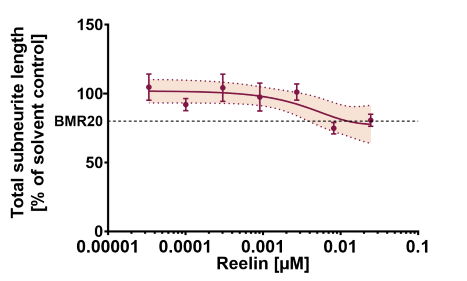


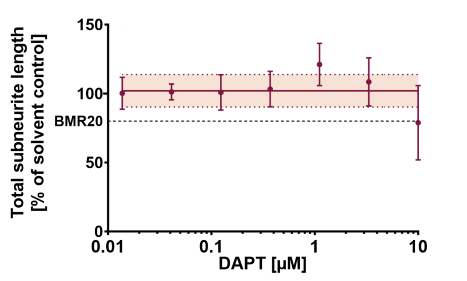

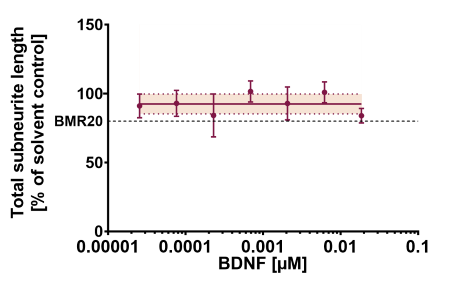


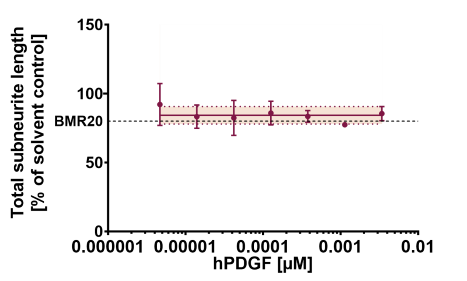

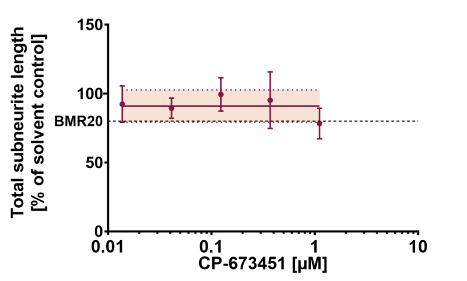


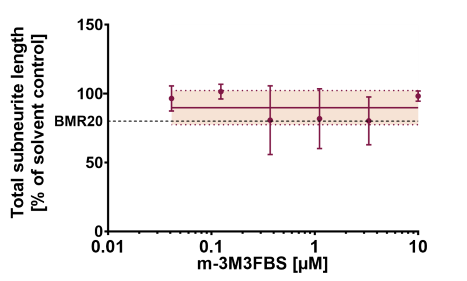


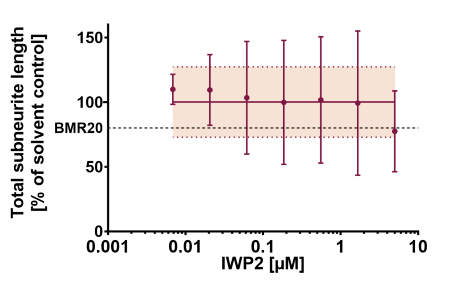


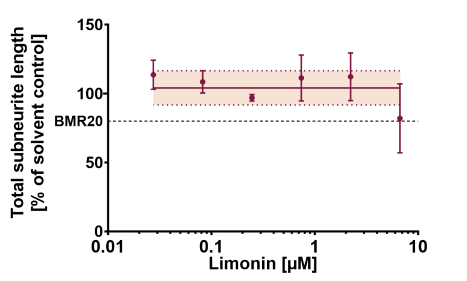


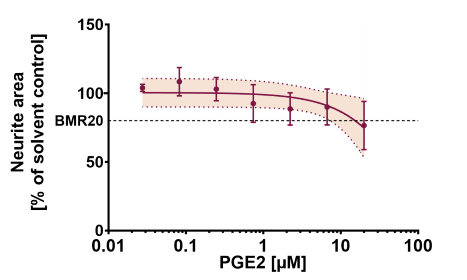


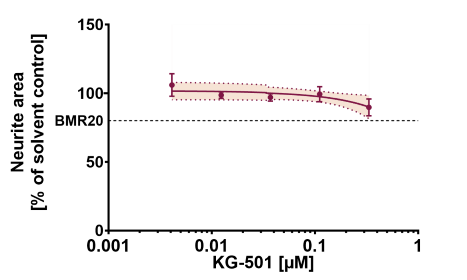


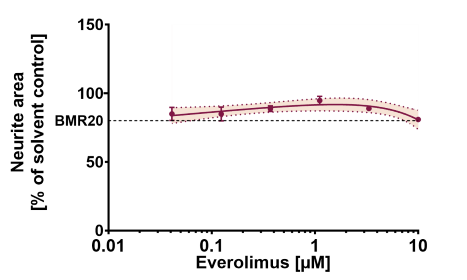

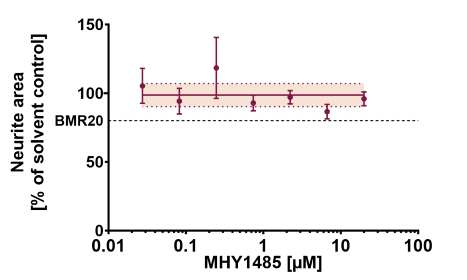


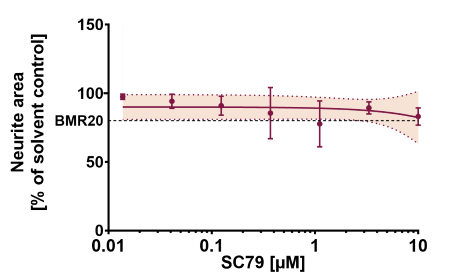

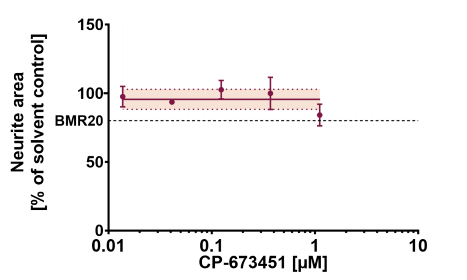


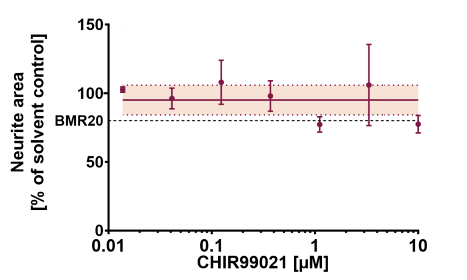

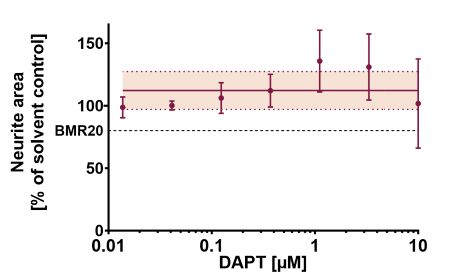


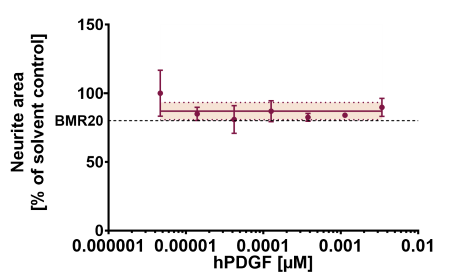

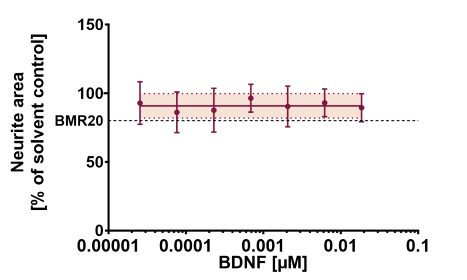


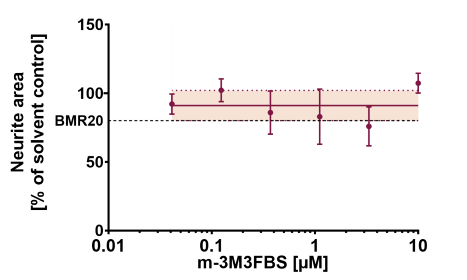


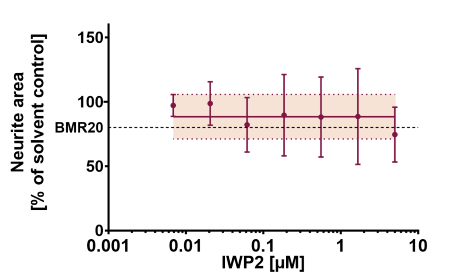


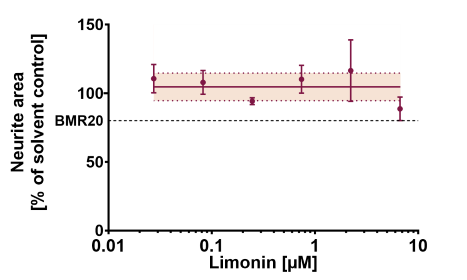


## NPC5

The NPC5 assay assesses OL differentiation. Primary hNPCs were exposed for 5 days to increasing concentrations of activators and inhibitors of the selected signaling pathways in a differentiation medium without growth factors on PDL-laminin-coated plates. OL differentiation was assessed as the percentage of O4-positive OLs compared to the total nuclei count within the migration area. Data are presented as mean ± SEM with dotted lines representing the lower and upper limit confidence intervals. Only no-effect results are shown in these supplemental files, see Figure 7 of the main manuscript for the effect results.


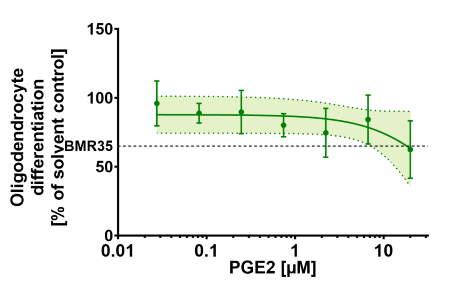

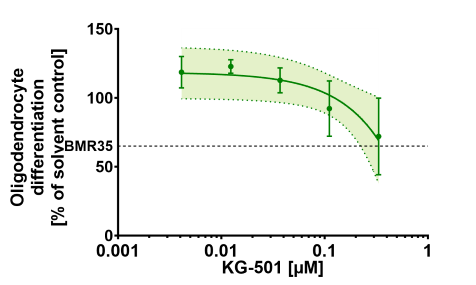


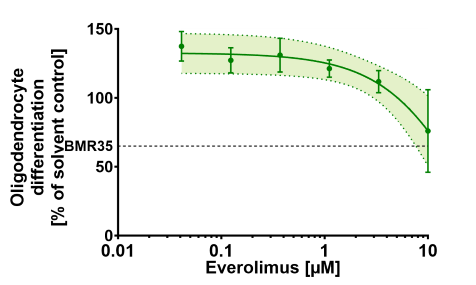

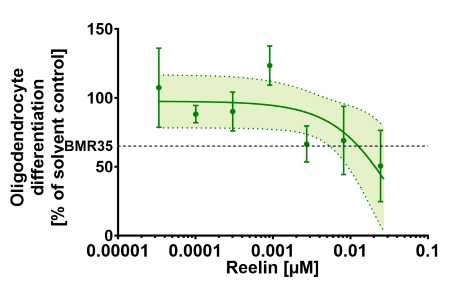


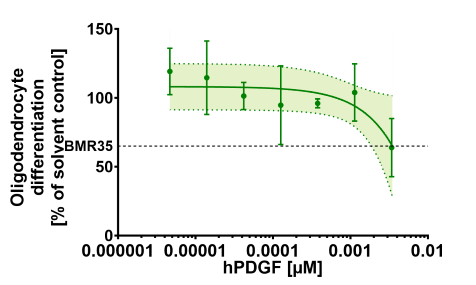

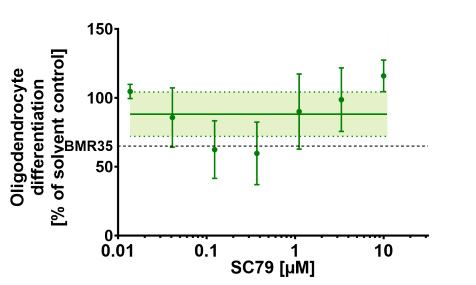


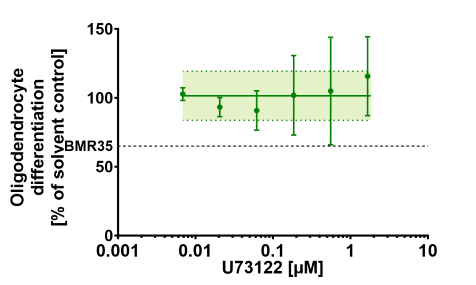


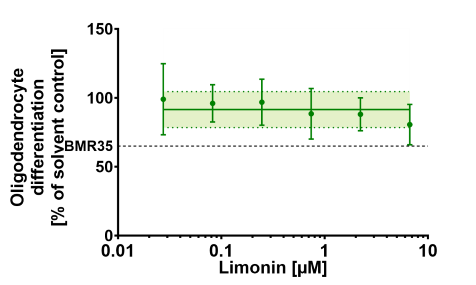


# Non-specific endpoints

Viability after 72 h of exposure (pages 21-23)

Viability after 120 h of exposure (pages 24-26)

Number of nuclei after 120 h of exposure (pages 27-29)

## VIABILITY 72 h

**Viability** (mitochondrial activity) was assessed using the CTB assay in hNPCs exposed for 3 days to increasing concentrations of activators and inhibitors of the selected signaling pathways in a proliferation medium containing growth factors EGF and FGF. The results are shown as a percentage of mitochondrial activity compared to the solvent control. Benchmark concentrations (BMCs) which caused a 10% reduction or induction in NPC viability according to the benchmark response (BMR10) specific to the viability assay are shown where applicable. Data are presented as mean ± SEM with dotted lines representing the lower and upper limit confidence intervals. *significant (p-value ≤ 0.05) compared to the respective solvent control calculated using the step-down multiple test procedure of Dunnett & Tamhane.

**Available BMCs:**

Rotenone – 0.039 µM

m-3M3FBS – 8.797 µM

## VIABILITY 120 h

**Viability** (mitochondrial activity) was assessed using the CTB assay in hNPCs exposed for 5 days to increasing concentrations of activators and inhibitors of the selected signaling pathways in a differentiation medium without growth factors on PDL-laminin-coated plates. The results are shown as a percentage of mitochondrial activity compared to the solvent control. Benchmark concentrations (BMCs) which caused a 10% reduction or induction in NPC viability according to the benchmark response (BMR10) specific to the viability assay are shown where applicable. Data are presented as mean ± SEM with dotted lines representing the lower and upper limit confidence intervals. *significant (p-value ≤ 0.05) compared to the respective solvent control calculated using the step-down multiple test procedure of Dunnett & Tamhane.

**Available BMCs:**

Rotenone – 0.036 µM

Celecoxib – 0.352 µM

db-cAMP – 2726.043 µM

m-3M3FBS – 3.81 µM

DAPT – 0.029 µM

## CELL NUMBER 120 h

**Cell number** was assessed as the total number of Hoechst33258-stained nuclei in hNPCs exposed for 5 days to increasing concentrations of activators and inhibitors of the selected signaling pathways in a differentiation medium without growth factors on PDL-laminin-coated plates. The results are shown as a percentage compared to the solvent control. Benchmark concentrations (BMCs) which caused a 25% reduction or induction in NPC viability according to the benchmark response (BMR25) specific to the nuclei number assessment are shown below. Data are presented as mean ± SEM with dotted lines representing the lower and upper limit confidence intervals. *significant (p-value ≤ 0.05) compared to the respective solvent control calculated using the step-down multiple test procedure of Dunnett & Tamhane.

**Available BMCs:**

Celecoxib – 1.995 µM

db-cAMP – 144.226 µM

cp-673451 – 0.306 µM

# Endpoint-specific positive controls

**NPC1**

Proliferation medium without growth factors was used as an endpoint-specific positive control and the data are available in the raw data sheets at the Biostudies repository under accession numbers starting at S-ONTX37 until S-ONTX57.

**NPC2-5**

Assay performance of the NPC2–NPC5 endpoints was verified using a set of endpoint-specific positive controls applied on dedicated validation plates: 10 µM PP2 for radial glia migration (NPC2a), 20 ng/mL EGF for inhibition of neuronal differentiation (NPC3), and 100 ng/mL BMP7 for inhibition of oligodendrocyte differentiation (NPC5). One validation plate per experimental day has been prepared. The experiments in this manuscript were conducted at the time of our EFSA-funded project, and thus the positive control plates for NPC2-5 have already been published together with the EFSA dataset – see figure 3 in Blum et al. 2023.

1. Shown level is of cAMP, the endogenous chemical and not of db-cAMP, its synthetic analogue. [↑](#footnote-ref-1)
